# Chrysosporazines Revisited: Regioisomeric Phenylpropanoid Piperazine P-Glycoprotein Inhibitors from Australian Marine Fish-Derived Fungi

**DOI:** 10.3390/molecules27103172

**Published:** 2022-05-16

**Authors:** Amila Agampodi Dewa, Zeinab G. Khalil, Ahmed H. Elbanna, Robert J. Capon

**Affiliations:** Institute for Molecular Bioscience, The University of Queensland, St. Lucia, QLD 4072, Australia; a.agampodidewa@imb.uq.edu.au (A.A.D.); z.khalil@uq.edu.au (Z.G.K.); ahmed.elbanna@pharma.cu.edu.eg (A.H.E.)

**Keywords:** chrysosporazine, brasiliamide, phenylpropanoid piperazine, P-glycoprotein inhibitor, fungal natural product, *Chrysosporium*, *Aspergillus*, *Spiromastix*, biodiscovery

## Abstract

A library of fungi previously recovered from the gastrointestinal tract (GIT) of several fresh, commercially sourced Australian mullet fish was re-profiled for production of a rare class of phenylpropanoid piperazine alkaloids (chrysosporazines) using an integrated platform of; (i) miniaturized 24-well plate cultivation profiling (MATRIX), (ii) UPLC-DAD and UPLC-QTOF-MS/MS (GNPS) chemical profiling, and; (iii) precursor directed biosynthesis to manipulate in situ biosynthetic performance and outputs; to detect two new fungal producers of chrysosporazines. Chemical analysis of an optimized PDA solid phase cultivation of *Aspergillus* sp. CMB-F661 yielded the new regioisomeric chrysosporazine T (**1**) and U (**2**), while precursor directed cultivation amplified production and yielded the very minor new natural products azachrysosporazine T1 (**3**) and U1 (**4**), and the new unnatural analogues neochrysosporazine R (**5**) and S (**6**). Likewise, chemical analysis of an optimized M1 solid phase cultivation of *Spiromastix* sp. CMB-F455 lead to the GNPS detection of multiple chrysosporazines and brasiliamides, and the isolation and structure elucidation of chrysosporazine D (**7**) and brasiliamide A (**8**). Access to new chrysosporazine regioisomers facilitated structure activity relationship investigations to better define the chrysosporazine P-glycoprotein (P-gp) inhibitory pharmacophore, which is exceptionally potent at reversing doxorubrin resistance in P-gp over expressing colon carcinoma cells (SW600 Ad300).

## 1. Introduction

As part of our ongoing investigations into the chemistry of Australian marine derived fungi, we assembled a library of fungal isolates from the gastrointestinal tract (GIT) of samples of three fresh, locally caught and commercially sourced mullet fish. This library of ~500 fungal isolates each with a unique chemical (UPLC-DAD) profile proved remarkably rich in new and unusual natural products. We have recently reported on studies into a handful of these isolates, including; (i) a 2019 account of rare lipodepsipeptide scopularides from *Scopulariopsis* spp. CMB-F458 and CMB-F115, and *Beauveria* sp. CMB-F585 [1]; (ii) a 2018 account of Schiff base prolinimines and a 2021 account of the unprecedented cryptic hydrazine *N*-amino-l-proline methyl ester from *Evlachovaea* sp. CMB-F563 [2], the discovery of the latter challenging commonly held perceptions on what it is to be a natural product [3]; a 2022 account of PKS-NRPS macrolide metarhizides and aromatic glycoside metarhizosides from *Metarhizium* sp. CMB-F624 [4]; and (iii) a 2019 account of structurally diverse *N*-benzoyl phenylpropanoid piperazine chrysosporazines *Chrysosporium* sp. CMB-F214 [5], and a 2020 account of *N*-cinnamoyl chrysosporazines from *Chrysosporium* sp. CMB-F294 [6]. The chrysosporazines were particular noteworthy, being non-cytotoxic to bacterial and fungal pathogens, and human carcinoma cells in vitro, but exhibiting promising inhibitory activity against the multi-drug resistance ABC transporter efflux pump P-glycoprotein (P-gp). It is well-known that the upregulation of ABC transporters is a key determinant in efflux-mediated multidrug resistance (MDR) across multiple pathogens and cancers, making such transporters an attractive target for therapeutic intervention. With efforts to develop clinically useful inhibitors proving elusive to date, discovery of the chrysosporazine P-gp inhibitory pharmacophore was noteworthy and deserving of continued study. In order to put our latest chrysosporazine contribution in context, it is important to briefly review chrysosporazine chemistry and biology.

**Figure 1:**
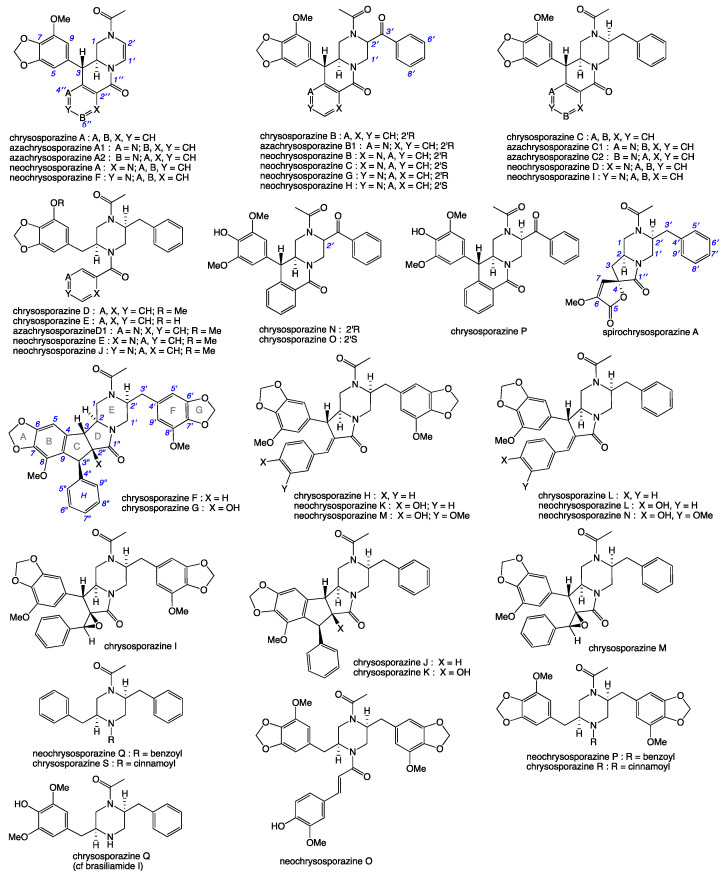
Previously reported chrysosporazines, azachrysosporazines, spirochrysosporazine and neochrysosporazines.

**Figure 2:**
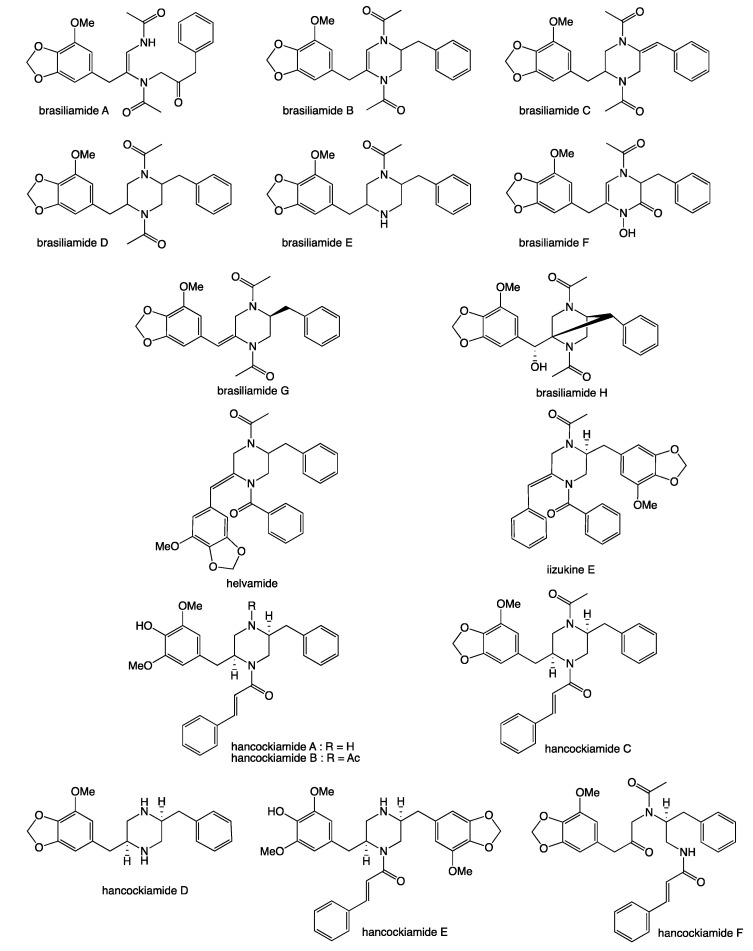
Previously reported brasiliamides, hancockiamides, iizukine E and helvamide.

Our 2019 account of chrysosporazines A–E from rice grain cultivations of *Chrysosporium* sp. CMB-F214 [5], and a subsequent 2020 account of chrysosporazines F–M from solid phase YES cultivations of *Chrysosporium* sp. CMB-F294 [6], presented some unusual challenges, not least being the structure elucidation of equilibrating mixtures of acetamide rotamers. Having successfully confirmed structures inclusive of the absolute configuration we established that while the chrysosporazines were not cytotoxic (i.e., did not inhibit the growth of bacterial, fungal or human carcinoma cells,) selected examples were very effective at reversing doxorubrin resistance in P-gp over expressing colon carcinoma cells (SW600 Ad300). Indeed, chrysosporazine B exhibited an impressive 3.2- fold increase (FI) in inhibitory potency compared to the positive control verapamil, as measured by the ratio of the respective gain in sensitivity (GS) values, where GS is the ratio of increased sensitivity (IC_50_) of SW600 Ad300 cells treated with doxorubicin, with versus without analyte supplementation (i.e., 2.5 μM chrysosporazine B or verapamil). Buoyed by this discovery, and keen to further advance the structure activity relationship (SAR) investigations, in 2021 we reported a successful detection, production, isolation, characterisation, identification and evaluation of the P-gp inhibitory properties of an array of new chrysosporazines, including minor azachrysosporazines [7], and followed this with a 2022 account of precursor directed unnatural neochrysosporazines [8]. These latter two investigations were assisted by; (i) cultivation profiling using a miniaturized 24-well plate microbioreactor format (known in lab as the MATRIX), which allowed for solid as well as static and shaken broth culture formats, and a wide array of media compositions, including the addition of natural and unnatural biosynthetic precursors; (ii) a rapid UPLC-QTOF-MS/MS and global natural products social (GNPS) molecular networking analysis to rapidly detect and prioritize new from known chrysosporazines in complex extracts and fractions; (iii) precursor directed biosynthesis using cultivation supplementation with natural substrates to amplify the production of exceptionally minor natural aza analogues (i.e., azachrysosporazines); and (iv) precursor directed biosynthesis using cultivation supplementation with unnatural substrates to repurpose the biosynthetic machinery to produce new analogues (i.e., neochrysosporazines). Collectively, these approaches proved highly effective at inducing *Chrysosporium* sp. CMB-F214 and CMB-F294 to produce a wide array of structurally diverse chrysosporazines. For example, solid phase M1 cultures of CMB-F214 supplemented with sodium nicotinate upregulated and facilitated access to the minor co-metabolite azachrysosporazines A1–A2, B1, C1–C2 and D1, chrysosporazines N–P and spirochrysosporazine [7], while supplementation with sodium picolinate yielded neochrysosporazines A–E, and with sodium isonicotinate yielded neochrysosporazines F–J [8]. By contrast, solid phase M2 cultures of CMB-F214 without precursor supplementation yielded the new chrysosporazine Q [7]. Building on this, solid phase YES cultures of CMB-F294 supplemented with sodium coumarate yielded the new neochrysosporazines K–L and chrysosporazines R–S, while sodium ferulate yielded the new neochrysosporazines M–O, and sodium benzoate yielded the new chrysosporazines R–S, neochrysosporazines P–Q [8], and the previously reported hancockiamide C [9]. In this context, the chrysosporazine and azachrysosporazine trivial nomenclature refers to biosynthetically-related natural products, and neochrysosporazine refers to precursor-derived unnatural products. To guide discussion we apply the collective term *chrysosporazines* to encompass all known members of this extended structure class, including chrysosporazines [5,6], azachrysosporazines [7], and spirochrysosporazine [7] and neochrysosporazines [8] (Figure 1), as well as the biosynthetically related hancockiamides [9], brasiliamides [10,11,12,13,14,15,16,17,18], iizukine E [19] and helvamide [20] (Figure 2).

**Figure 3:**
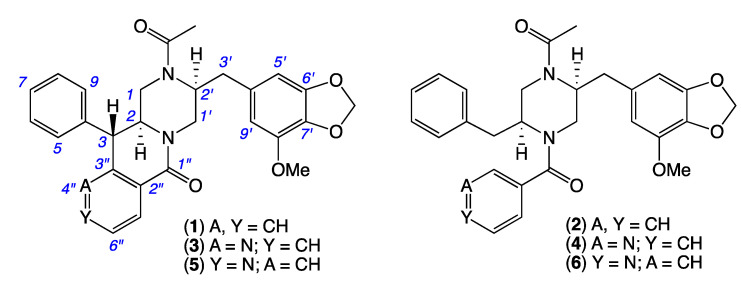
New *chrysosporazines* **1**–**6** from *Aspergillus* sp. CMB-F661.

Armed with over 50 new natural and unnatural *chrysosporazines* produced by the manipulation of *Chrysosporium* spp. CMB-F214 and CMB-F294 culture conditions, our 2022 report described a structure activity relationship (SAR) analysis that defined critical elements of the chrysosporazine P-gp inhibitory pharmacophore [8]. Notwithstanding this achievement, we were keen to access new and structurally diverse chrysosporazines to further advance SAR investigations. This current report describes just such an extension, informed by the discovery of the new chrysosporazines T (**1**) and U (**2**), azachrysosporazines T1 (**3**) and U1 (**4**), and neochrysosporazines R (**5**) and S (**6**) from *Aspergillus* sp. CMB-F661, and the known chrysosporazine D (**7**) and brasiliamide A (**8**) from *Spiromastix* sp. CMB-F455 (Figure 3).

## 2. Results and Discussion

**Figure 4:**
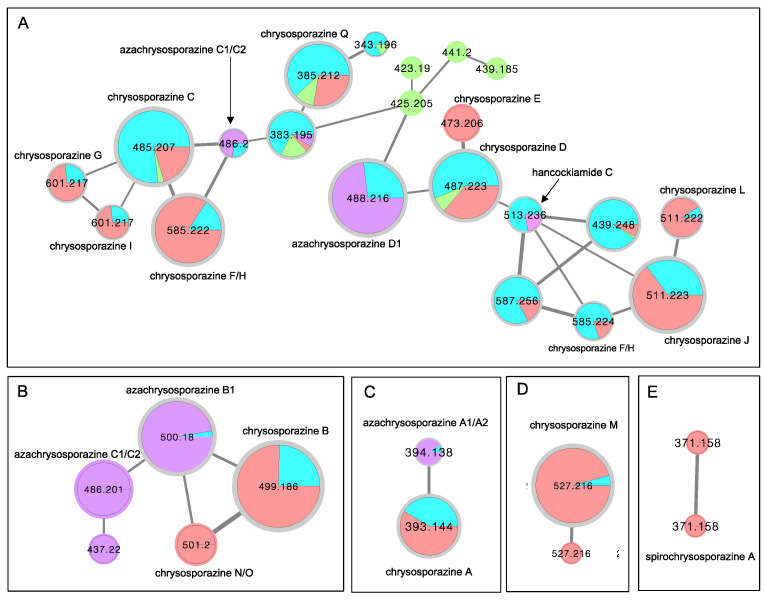
Selected molecular network families (**A**–**E**) from a GNPS analysis of PDA solid phase cultivations of *chrysosporazine* producing fungi CMB-F214 and CMB-F294 (blue), mapped against authentic standards of chrysosporazines (red), azachrysosporazines (purple) and hancockiamide C (pink), and ×100 other marine fish GIT-derived co-isolated fungi (green). (**A**) chrysosporazines C-J and L, azachrysosporazines C1/C2 and D1, and hancockiamide C; (**B**) chrysosporazines B and N/O, and azachrysosporazines B1 and C1/C2; (**C**) chrysosporazines A, azachrysosporazines A1 and A2; (**D**) chrysosporazines M; (**E**) spirochrysosporazine A.

As a first step in furthering our investigations into *chrysosporazines* we set out to determine if Global Natural Products Social (GNPS) molecular networking [21,22] could be used to detect *chrysosporazines* in unfractionated cultivation extracts. A GNPS molecular network analysis of PDA solid phase cultivations of the known *chrysosporazine* producing fungi CMB-F214 and CMB-F294 (Figure 4 blue highlights), was mapped against authentic standards of chrysosporazines (Figure 4 red highlights), azachrysosporazines (Figure 4 purple highlights), hancockiamide C (Figure 4 pink highlight), as well as ×100 other marine fish GIT-derived co-isolated fungi. This ×families (Figure 4A–E). Family A featured nodes attributed to chrysosporazines C–J and L, azachrysosporazines C1/C2 and D1, and hancockiamide C; Family B to chrysosporazines B and N/O, and azachrysosporazines B1 and C1/C2; Family C to chrysosporazine A and azachrysosporazines A1 and A2; Family D to chrysosporazine M; and Family E to spirochrysosporazine A. Significantly, when compared to the GNPS profiles from ×100 other marine fish GIT-derived co-isolated fungi, *Aspergillus* sp. CMB-F661 and *Spiromastix* sp. CMB-F455 were observed to produce metabolites that co-clustered with Family A (Figure 4 green highlights). What follows is an account of chemical investigations into CMB-F661 and CMB-F455.

To guide chemical investigations into CMB-F661, a media MATRIX cultivation profiling approach was applied to better understand and optimize conditions for the production of *chrysosporazines*. This entailed multiple cultivations of CMB-F661 in a 24-well microbioreactor format using ×11 different culture media (Table S1, Figure S4), across both static and shaken broths (1.5 mL) and solid agar (1 g). Each microbioreactor well cultivation was in turn extracted in situ with EtOAc (2 mL) to provide extracts (×33) that were subjected to UPLC-DAD (210 nm) analysis to reveal variable levels of *chrysosporazine* production (Figure S5). These observations were further confirmed and quantified by GNPS analysis of all media MATRIX extracts (Figure S6). Based on these analyses, a PDA solid phase was selected for scale up cultivation of CMB-F661, as it was deemed capable of producing two new major chrysosporazines **1**–**2** and potentially two new minor azachrysosporazines **3**–**4**. While subsequent chemical fractionation of a scaled up PDA cultivation of CMB-F661 using a combination of solvent extraction, partitioning and trituration and reversed phase chromatography (Scheme S1) yielded pure samples of **1–2**, the level of production of **3**–**4** were too low for successful isolation. Based on our earlier investigations into azachrysosporazines from *Chrysosporium* sp. CMB-F214 [7], we speculated that **3**–**4** differed from **1**–**2** by the incorporation of a nicotinamide rather than a benzamide moiety, and that supplementation of CMB-F661 cultivations with sodium nicotinate would enhance production levels to the point where isolation, characterisation and structure elucidation was feasible. As predicted, comparable fractionation of PDA cultivations of CMB-F661 supplemented with sodium nicotinate yielded two new minor azachrysosporazines **3**–**4**. Building on this success, fractionation of PDA cultivations of CMB-F661 supplemented with sodium isonicotinate yielded two new neochrysosporazines **5**–**6**. An account of the structure elucidation of **1**–**6** (Figure 3) is summarised below.

**Figure 5:**
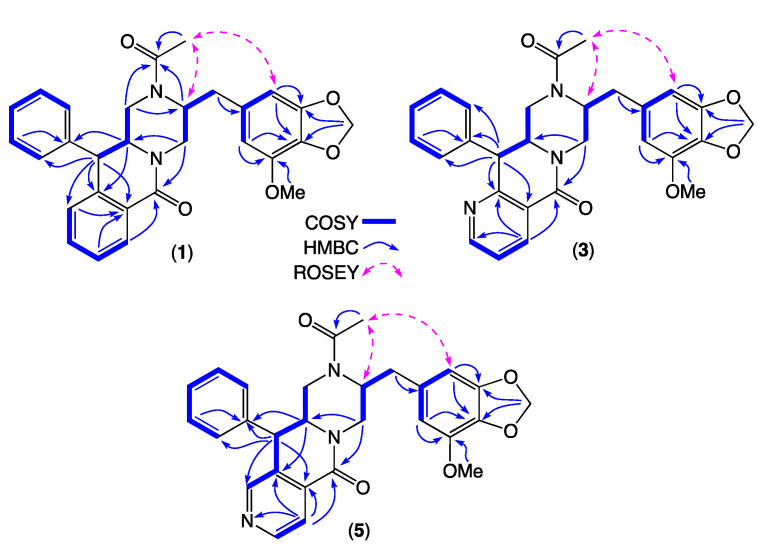
Diagnostic 2D NMR (DMSO-*d_6_*) correlations for **1**, **3** and **5**.

**Figure 6:**
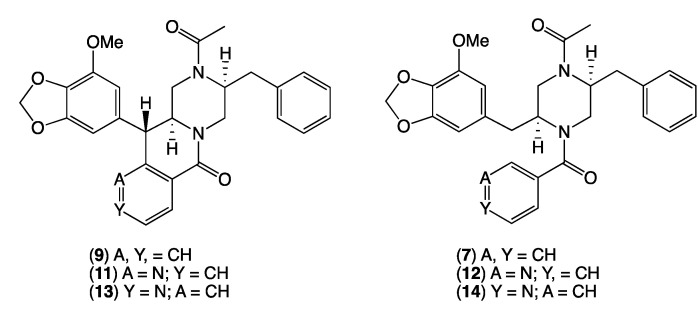
Known chrysosporazine C (**9**), azachrysosporazine C1 (**11**) and neochrysosporazine I (**13**), and *seco* analogues, chrysosporazine D (**7**), azachrysosporazine D1 (**12**) and neochrysosporazine J (**14**).

HRESI(+)MS analysis of 1 revealed a molecular formula (C29H28N2O5) isomeric with chrysosporazine C (9) [5] (Figure 3, Figure 5 and Figure 6). Comparison of the NMR (DMSO-*d*_6_) data for **1** (Table 1, Table 2 and Table S3, Figures S14–S21) with **9** revealed characteristic major and minor acetamide rotamers (3:1 ratio), respectively, with the major rotamer in **1** assigned an *E* configuration based on ROESY correlations between the acetamide methyl and H-2′. Similarly, a ROESY correlation between the acetamide methyl and H-5′ confirmed an alternate aromatic regiochemistry in **1** compared to **9**. The full planar structure for **1** was confirmed by diagnostic 2D NMR correlations (Figure 5), while comparisons of ^1^H NMR data for methines associated with the three chiral centres in **1** (H-2 δ_H_ 3.84, ddd, *J* 10.6, 10.6, 3.9 Hz; H-3 δ_H_ 4.47, d, *J* 10.6 Hz; H_a_-3′ δ_H_ 2.90, dd, *J* 13.5, 8.1 Hz; H_b_-3′ δ_H_ 2.86, dd, *J* 13.5, 6.6 Hz) and **9** (H-2 δ_H_ 3.85, ddd, *J* 11.0, 11.0, 3.9 Hz; H-3 δ_H_ 4.38, d, *J* 11.0 Hz; H_a_-3′ δ_H_ 3.04, dd, *J* 13.3, 8.9 Hz; H_b_-3′ δ_H_ 2.92, dd, *J* 13.1, 6.1 Hz) supported a common relative configuration.

**Figure 7:**
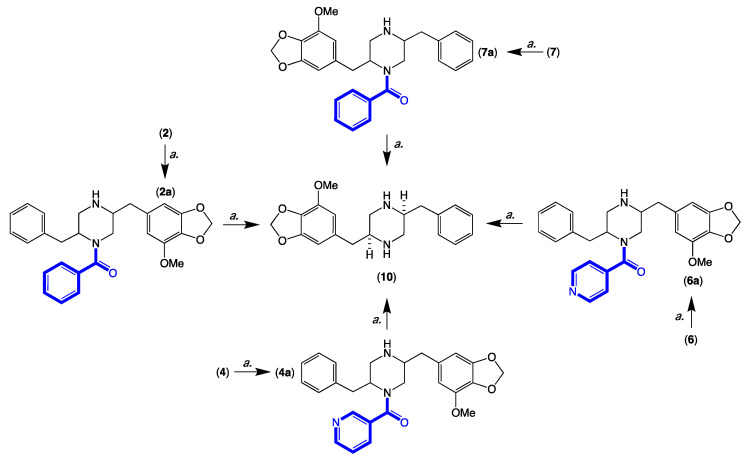
Acid hydrolysis (*a.* 1 M HCl, 36 h, 100 °C) and correlation of **2**, **4**, **6** and **7**, to **10**.

HRESI(+)MS analysis of **2** revealed a molecular formula (C_29_H_30_N_2_O_5_) in common with chrysosporazine D (**7**) [5] (Figure 3 and Figure 6). As with **7**, the NMR (DMSO-*d*_6_) data for **2** (Figures S22 and S23) was extremely broad, consistent with equilibration between multiple acetamide and benzamide rotamers. To confirm this hypothesis, samples of **2** and **7** were subjected to partial acid hydrolysis with HPLC-MS analysis detecting peaks attributed to deacetylated **2a** (from **2**) (*m*/*z* 445, M + H, C_26_H_28_N_2_O_4_) and **7a** (from **7**) (*m*/*z* 445, M + H, C_26_H_28_N_2_O_4_), which did not co-elute during UPLC-DAD analysis (Figure 7, Figures S45 and S48), confirming that **2** and **7** were indeed isomers. Acid hydrolysis of **2** and **7** also yielded the fully hydrolysed **10** (*m*/*z* 341, M + H, C_20_H_24_N_2_O_3_), confirming a common core phenylpropanoid piperazine scaffold.

HRESI(+)MS analysis of **3** and **5** returned molecular formulae (C_28_H_27_N_3_O_5_) isomeric with azachrysosporazine C1 (**11**) [7] and neochrysosporazine I (**13**) [8] (Figure 3 and Figure 6). Comparison of the NMR (DMSO-*d*_6_) data for **3** (Table 1, Table 2 and Table S4, Figures S24–S30) and **5** (Table 1, Table 2 and Table S5, Figures S33–S39) with **11** and **13** revealed many similarities, including the presence of major (*E*) and minor (*Z*) acetamide rotamers, and a cyclized *N*-nicotinamide in **3** and *N*-isonicotinamide in **5**. These observations were confirmed by diagnostic 2D NMR correlations (Figure 5) together with chemical shifts and multiplicities for chiral centre methines in **3** (H-2 δ_H_ 3.87, ddd, *J* 11.2, 8.8, 3.8 Hz; H-3 δ_H_ 4.56, d, *J* 8.8 Hz; H_a_-3′ δ_H_ 2.90, dd, *J* 13.5, 8.3 Hz; H_b_-3′ δ_H_ 2.86, dd, *J* 13.5, 6.3 Hz), and **5** (H-2 δ_H_ 3.92, ddd, *J* 14.5, 10.3, 3.7 Hz; H-3 δ_H_ 4.56, d, *J* 10.3 Hz; H_a_-3′ δ_H_ 2.90, dd, *J* 13.4, 8.6 Hz; H_b_-3′ δ_H_ 2.85, dd, *J* 13.4, 6.4 Hz), which together with biosynthetic considerations permitted assignment of the full structure for azachrysosporazine T1 (**3**) and neochrysosporazine R (**5**), as shown.

HRESI(+)MS analysis of **4** and **6** revealed molecular formulae (C_28_H_29_N_3_O_5_) in common with azachrysosporazine D1 (**12**) [7] and neochrysosporazine J (**14**) [8] (Figure 3 and Figure 6). As reported for **12** and **14**, the NMR (DMSO-*d*_6_) data for **4** (Figures S31–S32) and **6** (Figures S40 and S41) were extremely broad and precluded structure elucidation through direct spectroscopic analysis. To overcome this limitation, both **4** and **6** were subjected to partial acid hydrolysis with HPLC-DAD-MS analysis detecting peaks attributed to the deacetylated products **4a** (from **4**) and **6a** (from **6**) (*m/z* 446, M + H, C_26_H_27_N_3_O_4_), along with fully hydrolysed **10** (from both **4** and **6**) (Figure 7 and Figures S46–S48), confirming a common core phenylpropanoid piperazine scaffold with **7** (and **12** and **14**). Significantly, unique UPLC retention times for **4**, **6**, **12** and **14**, and/or their deacetylated hydrolysis products **4a**, **6a**, **12a** and **14a** (Figure S48), confirmed that **4** and **6** were isomeric (and not identical) with **12** and **14**, respectively.

Assignment of absolute configurations to **1**–**6** were made on the basis of [α]_D_ comparisons with regioisomers of known absolute configuration. For example, chrysosporazine T (**1)** (−10.4) was compared to chrysosporazine C (**9**) (−9.7) [5]; chrysosporazine U (**2**) (+19.6) to chrysosporazine D (**7**) (+19.7) [5]; azachrysosporazine T1 (**3**) (−12.9) to azachrysosporazine C1 (**11**) (−29.6) [7]; azachrysosporazine U1 (**4**) (+5.5) to azachrysosporazine D1 (**12**) (+19.3) [7]; neochrysosporazine R (**5**) (−21.7) to neochrysosporazine I (**13**) (−27.7) [8]; and neochrysosporazine S (**6**) (+2.9) to neochrysosporazine J (**14**) (+27.6) [8]. These proposed assignments confirm a common absolute configuration across all known chrysosporazines, azachrysosporazines and neochrysosporazines, consistent with their common biosynthetic origins.

Following the strategy outlined above for CMB-F661, a media MATRIX cultivation profiling and GNPS analysis of *Spiromastix* sp. CMB-F455 (Figures S9–S12 and Table S2) detected an array of *chrysosporazines*, with molecular formulae consistent with brasiliamides A/H (C_24_H_26_N_2_O_6_), B/C/G (C_24_H_26_N_2_O_5_), D (C_24_H_28_N_2_O_5_) and E (C_22_H_26_N_2_O_4_), and chrysosporazines C (C_29_H_28_N_2_O_5_), D (C_29_H_30_N_2_O_5_) and Q (C_22_H_28_N_2_O_4_). Subsequent fractionation of a scaled up M1 agar cultivation of CMB-F455 yielded the two known natural products chrysosporazine D (**7**) [5] and brasiliamide A (**8**) [10], with structures assigned on the basis of detailed spectroscopic analysis and comparison to literature data (Table S6 and Figures S42–S44). Efforts at precursor directed biosynthesis, with M1 media cultivations of CMB-F455 supplemented with the sodium salts of either nicotinic, isonicotinic, benzoic, picolinic, cinnamic, caffeic and coumaric acids, failed to induce the production of new *chrysosporazines* (Figure S13). This is the first account of *chrysosporazines* from the genus *Spiromastix* and is the first reported occurrence of chrysosporazines as co-metabolites with brasiliamides, supporting the proposition of a shared biosynthetic origin.

**Figure 8:**
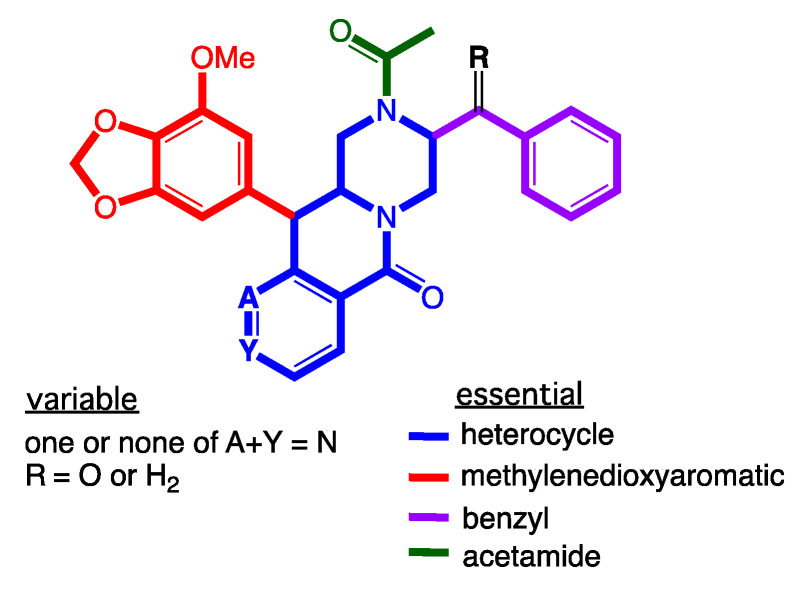
SAR analysis of the chrysosporazine P-gp inhibitory pharmacophore.

Consistent with all our earlier studies, **1**–**8** did not exhibit any growth inhibitory properties (IC_50_ > 30 µM) against the Gram-positive and Gram-negative bacteria, the fungus *Candida albicans* (Figure S49), and cytotoxicity (IC_50_ > 30 µM) against human colorectal carcinoma (SW620) or P-glycoprotein (P-gp) overexpressing multidrug resistant human colorectal carcinoma (SW620 Ad300) cells (Figure S50, Table S7), although selected examples did inhibit P-gp and effect a reversal of MDR. For example, where analogues lacking a C-3/C-3″ cyclization motif exhibited little or no reversal of doxorubicin resistance in SW620 Ad300 carcinoma cells, as evidenced by low fold increases (FI) for **2** (FI 0.25), **4** (FI 0.25), **6** (FI 0.36), **7** (FI 0.16), **8** (FI 0.13) (Table 3, Figure S50), a significant reversal of doxorubicin resistance was observed for **1** (FI 0.61), **3** (FI 0.80) and **5** (FI 1.22) (Table 3, Figure S50), albeit weaker than the previously reported regioisomers **9** (FI 2.28) and **11** (FI 2.63). A structure activity relationship (SAR) analysis based on these results suggests the regiochemistry of the methylenedioxy substituted aromatic ring is a key determinant for improved P-gp inhibition (Figure 8).

## 3. Materials and Methods

### 3.1. General Experimental Procedures

Chiroptical measurements ([α]_D_) were obtained on a JASCO P-1010 polarimeter (JASCO International Co. Ltd., Tokyo, Japan) in a 100 × 2 mm cell at specified temperatures. Electronic Circular Dichroism (ECD) measurements were obtained on a JASCO J-810 spectropolarimeter (JASCO International Co. Ltd., Tokyo, Japan) in a 0.1 cm path-length cell. Nuclear magnetic resonance (NMR) spectra were acquired on a Bruker Avance 600 MHz spectrometer (Bruker Pty. Ltd., Alexandria, NSW, Australia) with either a 5 mm PASEL ^1^H/D-^13^C Z-Gradient probe or 5 mm CPTCI ^1^H/^19^F-^13^C/^15^N/DZ-Gradient cryoprobe. In all cases, spectra were acquired at 25 °C deuterated solvents as indicated, with referencing to residual ^1^H or ^13^C signals. High-resolution ESIMS spectra were obtained on a Bruker micrOTOF mass spectrometer (Agilent Technologies Inc., Mulgrave, VIC, Australia) (by direct injection in MeOH at 3 μL/min using sodium formate clusters as an internal calibrant. Liquid chromatography-diode array-mass spectrometry (HPLC-DAD-MS) data were acquired either on an Agilent 1260 series (Agilent Technologies Inc., Mulgrave, VIC, Australia) separation module equipped with an Agilent G6125B series LC/MSD mass detector (Agilent Technologies Inc., Mulgrave, VIC, Australia) and diode array detector. Semi-preparative HPLCs were performed using Agilent 1100 series HPLC instruments with corresponding detectors, fraction collectors and software inclusively. UPLC chromatograms were obtained on an Agilent 1290 infinity UPLC system equipped with a diode array multiple wavelength detector (Agilent Technologies Inc., Mulgrave, VIC, Australia) (Zorbax C_8_ RRHD 1.8 μm, 50 × 2.1 mm column, 0.417 mL/min with a 2.50 min gradient from 90% H_2_O/MeCN to MeCN with a constant 0.01% TFA modifier). A UPLC-QTOF analysis was performed on a UPLC-QTOF instrument comprising an Agilent 1290 Infinity II UPLC (Agilent Technologies Inc., Mulgrave, VIC, Australia) (Zorbax C_8_ RRHD 1.8 μm, 50 × 2.1 mm column, eluting at 0.417 mL/min with a 2.50 min gradient elution from 90% H_2_O/MeCN to 100% MeCN with a constant 0.1% formic acid modifier) coupled to an Agilent 6545 Q-TOF. MS/MS analysis was performed on the same instrument for ions detected in the full scan at an intensity above 1000 counts at 10 scans/s, with an isolation width of 4 ~*m*/*z* using a fixed collision energy and a maximum of three selected precursors per cycle. Chemicals were purchased from Sigma-Aldrich or Merck, Australia unless otherwise specified. Analytical-grade solvents were used for solvent extractions. Chromatography solvents were of HPLC grade supplied by Labscan or Sigma-Aldrich, Australia and filtered/degassed through 0.45 μm polytetrafluoroethylene (PTFE) membrane prior to use. Deuterated solvents were purchased from Novochem, Australia. Microorganisms were manipulated under sterile conditions using a Laftech class II, Australia biological safety cabinet and incubated in either MMM Friocell incubators (Lomb Scientific, NSW, Australia) or an Innova 42R incubator shaker (John Morris, NSW, Australia).

### 3.2. Fungal Isolation

The fungi CMB-F661 and CMB-F455 were isolated from the gastrointestinal tract of a specimen of Mugil mullet fish, purchased from Australian fish market in Brisbane, that was cultured on a PDA solid phase at 26.5 °C for seven days (Figure S1).

### 3.3. Fungal Taxonomy

Genomic DNA for CMB-F661 and CMB-F455 were extracted from the mycelia using the DNeasy Plant Mini Kit (Qiagen, Brisbane, Australia) as per the manufacturers protocol. The 18s rRNA gene was amplified by PCR using the universal primers ITS-1 (5′-TCCGTAGGTGAACCTGCGG-3′) and ITS-4 (5′-TCCTCCGCTTATTGATATGC-3′) purchased from Sigma-Aldrich. The PCR mixture (50 μL) contained genomic DNA (2 μL, 20–40 ng), EmeraldAmpn GT PCR Master Mix (2X Premix) (25 μL), primer (0.2 μM, each), and H_2_O (up to 50 μL). A PCR was performed using the following conditions: initial denaturation at 95 °C for 2 min, 30 cycles in series of 95 °C for 20 s (denaturation), 56 °C for 20 s (annealing) and 72 °C for 30 s (extension), followed by one cycle at 72 °C for 5 min. PCR products were purified with a PCR purification kit (Qiagen, Victoria, Australia). Amplification products were examined by agarose gel electrophoresis. The DNA sequencing was performed by the Australian Genome Research Facility (AGRF) at The University of Queensland, Brisbane, Australia. A BLAST analysis (NCBI database) on amplified ITS gene sequences for the two strains allowed identification of CMB-F661 as *Aspergillus* sp. (99% homology, GenBank accession no. MT529315.1) and CMB-F455 as *Spiromastix* sp. (98% homology, GenBank accession no. KP119636.1). A phylogenetic tree was constructed through PhyML Maximum Likelihood analysis using 18S rRNA as queries of known chrysosporazine producing *Chrysosporium* spp. CMB-F214 and CMB-F294, hancockiamide producing *Aspergillus hancockii,* and brasiliamide producing *Neosartorya hirustake* and *Neosartorya pseudofischeri,* along with top similar 18S rRNA sequences displayed after BLAST on the Refseq RNA NCBI database (Figure S2).

### 3.4. Global Natural Product Social (GNPS) Molecular Networking

Aliquots (1 μL) of dried fraction (100 μg/mL in MeOH) were analysed on an Agilent 6545 Q-TOF LC/MS equipped with an Agilent 1290 Infinity II UPLC system (Agilent Technologies Inc., Mulgrave, VIC, Australia), utilising an Agilent SB-C8 1.8 μm, 2.1 × 50 mm column, eluting with 90% H_2_O/MeCN to MeCN at a 0.417 mL/min over 2.5 min with an isocratic 0.1% formic acid modifier. UPLC-QTOF-(+)MS/MS data acquired for all samples at collision energy of 10, 20 and 40 eV were converted from Agilent MassHunter (Agilent Technologies Inc., Mulgrave, VIC, Australia) data files (.d) to mzXML file format using MSConvert software, and transferred to the GNPS server (gnps.ucsd.edu). Molecular networking was performed using the GNPS data analysis workflow [21,22], using the spectral clustering algorithm with a cosine score of 0.6 and a minimum of five matched peaks. The resulting spectral network was imported into Cytoscape software (version 3.7.1) [23] and visualized using a ball-stick layout where nodes represent parent masses and the cosine score was reflected by edge thickness. Also, group abundances were set as pie charts, which reflected the intensity of MS signals.

### 3.5. Fractionation of a Scaled Up PDA Culture of Aspergillus sp. CMB-F661

The fungus CMB-F661 was inoculated on PDA agar (×100 plates) and incubated at 26.5 °C for 12 days, after which the combined agar and mycelia were harvested, extracted with EtOAc (4 × 600 mL), and the filtered organic phase concentrated in vacuo at 40 °C to yield an extract (800 mg). This extract was triturated with *n*-hexane to yield after in vacuo concentration *n*-hexane (90 mg) and MeOH (710 mg) solubles. With UPLC-QTOF analysis localising the target chemistry in the MeOH solubles, a portion (400 mg) was fractionated by preparative reversed phase HPLC (Phenomenex Luna-C_8_ 10 mm, 21.2 × 250 mm column, with a 20 min 20 mL/min gradient elution from 90% H_2_O/MeCN to MeCN with an isocratic 0.1% TFA/MeCN modifier) to further concentrate the target chemistry into two fractions. Fraction A (10.2 mg) was subjected to a semi-preparative reversed phase HPLC (ZORBAX SB-C_3_ 5 μm, 9.4 × 250 mm column, with a 33 min 3 mL/min isocratic elution at 78% H_2_O/MeCN inclusive of a 0.1% TFA/MeCN modifier) to yield chrysosporazine T (**1**) (1.8 mg, 0.4%). Fraction B (8.5 mg) was subjected to semi-preparative reversed phase HPLC (Agilent) ZORBAX SB-C_3_ 5 μm, 9.4 × 250 mm column, with a 37 min 3 mL/min isocratic elution at 72% H_2_O/MeCN inclusive of a 0.1% TFA/MeCN modifier) to yield chrysosporazine U (**2**) (2.0 mg, 0.44%) (Figure S3 and Scheme S1). Note that all yields were estimated as that present in the unfractionated EtOAc extract.

*Chrysosporazine T* (**1**). White powder; [α]_D_^22.2^ − 10.4 (*c* 0.075, MeOH); NMR (600 MHz, DMSO-*d_6_*) see Table 1, Table 2 and Table S3, Figure 3, Figure 5 and Figures S14–S21; ESI(+)MS *m*/*z* 507 [M + Na]+; HRESI(+)MS *m*/*z* 507.1892 [M + Na]+ (calcd for C_29_H_28_N_2_O_5_Na, 507.1890).

*Chrysosporazine U* (**2**). White powder; [α]_D_^22.2^ + 19.6 (*c* 0.166, MeOH); NMR (600 MHz, DMSO-*d*_6_) see Figure 3, Figures S22 and S23; ESI(+)MS *m*/*z* 509 [M + Na]+; HRESI(+)MS *m*/*z* 509.2043 [M + Na] + (calcd for C_29_H_30_N_2_O_5_Na, 509.2047).

### 3.6. Media MATRIX Cultivation Profiling of Aspergillus sp. CMB-F661

The fungus CMB-F661 was cultured in a 24-well microbioreactor format using ×11 different culture media, across both static and shaken broths (1.5 mL) and solid agar (1 g slant), and extracted in situ with EtOAc (2 mL) to provide ×33 culture extracts [22]. The culture extracts were individually decanted, filtered, dried under N_2_ and the resulting residue subjected to UPLC-DAD and UPLC-QTOF analysis with an internal calibrant, and GNPS analysis, to detect and quantify levels of metabolite production (Figures S4–S6 and Table S1).

### 3.7. Precursor-Directed Biosynthesis Cultivation Profiling of Aspergillus sp. CMB-F661

The fungus CMB-F661 was cultured on individual PDA plates, each of which were supplemented with 2 mg/mL of the sodium salts of either nicotinic, isonicotinic, benzoic or picolinic acids. After 10 days of incubation the agar and mycelia from each plate were harvested together, extracted with EtOAc (15 mL), and subjected to UPLC-DAD and UPLC-QTOF analysis. While supplementation with sodium benzoate enhanced the production of **1**–**2**, supplementation with sodium nicotinate facilitated access to the minor natural product azachrysosporazines **3**–**4**, and sodium isonicotinate to the unnatural product neochrysosporazines **5**–**6** (Figures S7 and S8).

### 3.8. Scaled-Up Cultivation of Aspergillus sp. CMB-F661 on PDA with Sodium Nicotinate

A stock solution of sodium nicotinate was prepared by dissolving nicotinic acid (5 g) in a saturated aqueous solution of NaHCO_3_ (41.5 mL) and H_2_O (41.5 mL) (pH~7). This stock solution was added to warm PDA agar media (final conc of sodium nicotinate 2 mg/mL) which was stirred for 5 min then dispensed to agar plates. These agar plates (×100) were inoculated with the fungus *Aspergillus* sp. CMB-F661 and incubated at 26.5 °C for 14 days, after which they were extracted with EtOAc (4 × 500 mL), and the combined organic phase was filtered and concentrated in vacuo at 40 °C, to yield an extract (482 mg). The extract was then triturated with *n*-hexane to yield, after *in vacuo* concentration, *n*-hexane (98 mg) and MeOH (380 mg) solubles. With UPLC-QTOF analysis localising the target chemistry in the MeOH solubles, these were fractionated by preparative reversed phase HPLC (Phenomenex Luna-C_8_ 10 μm, 21.2 × 250 mm column, with a 20 min 20 mL/min gradient elution from 90% H_2_O/MeCN to MeCN inclusive of an isocratic 0.1% TFA/MeCN modifier) to further concentrate the target chemistry into two fractions. Fraction A (5.8 mg) was subjected to semi-preparative reversed phase HPLC (ZORBAX SB-C_3_ 5 μm, 9.4 × 250 mm column, with a 33 min 3 mL/min isocratic elution at 70% H_2_O/MeCN inclusive of a 0.1% TFA/MeCN modifier) to yield azachrysosporazine T1 (**3**) (0.9 mg, 0.2%). Fraction B (6.2 mg) was subjected to semi-preparative reversed phase HPLC (ZORBAX SB-C_3_ 5 μm, 9.4 × 250 mm column, with a 37 min 3 mL/min isocratic elution at 75% H_2_O/MeCN inclusive of a 0.1% TFA/MeCN modifier) to yield azachrysosporazine U1 (**4**) (1.0 mg, 0.2%) (Scheme S2). Note that all yields were estimated as that present in the unfractionated EtOAc extract.

*Azachrysosporazine T1* (**3**). White powder; [α]_D_^22.2^–12.9 (*c* 0.066, MeOH); NMR (600 MHz, DMSO-*d_6_*) see Table 1, Table 2 and Table S4, Figure 3, Figure 5 and Figures S24–S30; ESI(+)MS *m*/*z* 508 [M + Na]+; HRESI(+)MS *m*/*z* 508.1855 [M + Na]+ (calcd for C_28_H_27_N_3_O_5_Na, 508.1843).

*Azachrysosporazine U1* (**4**). White powder; [α]_D_^22.2^ + 5.5 (*c* 0.091, MeOH); NMR (600 MHz, DMSO-*d*_6_) see Figure 3 and Figures S31–S32; ESI(+)MS *m*/*z* 510 [M + Na]+; HRESI(+)MS *m*/*z* 510.1999 [M + Na]+ (calcd for C_28_H_29_N_3_O_5_Na, 510.1993).

### 3.9. Scaled-Up Cultivation of Aspergillus sp. CMB-F661 on PDA with Sodium Isonicotinate

A stock solution of sodium isonicotinate was prepared by dissolving isonicotinic acid (7.5 g) in saturated aqueous solution of NaHCO_3_ (62.2 mL) and H_2_O (62.2 mL) (pH ~7). This stock solution was added to warm PDA agar media (final conc of sodium nicotinate 2 mg/mL), which was stirred for 5 min and then dispensed to agar plates. These agar plates (×150) were inoculated with the fungus CMB-F661 and incubated at 26.5 °C for 18 days, after which they were extracted with EtOAc (4 × 500 mL), and the combined organic phase was filtered and concentrated in vacuo at 40 °C, to yield an extract (680 mg). The extract was then triturated with *n*-hexane to yield, after in vacuo concentration, *n*-hexane (102 mg) and MeOH (570 mg) solubles. With UPLC-QTOF analysis localising the target chemistry in the MeOH solubles, a portion (500 mg) was fractionated by preparative reversed phase HPLC (Phenomenex Luna-C_8_ 10 μm, 21.2 × 250 mm column, with a 20 min 20 mL/min gradient elution from 90% H_2_O/MeCN to MeCN inclusive of an isocratic 0.1% TFA/MeCN modifier) to further concentrate the target chemistry in two fractions. Fraction A (5.2 mg) was subjected to semi-preparative reversed phase HPLC (ZORBAX SB-C_3_ 5 μm, 9.4 × 250 mm column, with a 33 min 3 mL/min isocratic elution at 70% H_2_O/MeCN inclusive of 0.1% TFA/MeCN modifier) to yield neochrysosporazine R (**5**) (1.0 mg, 0.17%). Fraction B (5.9 mg) was subjected to semi-preparative reversed phase HPLC (ZORBAX SB-C_3_ 5 μm, 9.4 × 250 mm column, with a 37 min 3 mL/min isocratic elution at 75% H_2_O/MeCN inclusive of 0.1% TFA/MeCN modifier) to yield neochrysosporazine S (**6**) (0.7 mg, 0.12%) (Scheme S3). Note that all yields were estimated as that present in the unfractionated EtOAc extract.

*Neochrysosporazine R* (**5**). White powder; [α]_D_^22.2^ − 21.7 (*c* 0.083, MeOH); NMR (600 MHz, DMSO-*d_6_*) see Table 1, Table 2 and Table S5, Figure 3, Figure 5 and Figures S33–S39; ESI(+)MS *m*/*z* 508 [M + Na]+; HRESI(+)MS *m*/*z* 508.1851 [M + Na]+ (calcd for C_28_H_27_N_3_O_5_Na, 508.1843).

*Neochrysosporazine S* (**6**). White powder; [α]_D_^22.2^ + 2.9 (*c* 0.066, MeOH); NMR (600 MHz, DMSO-*d*_6_) see Figure 3 and Figures S40–S41; ESI(+)MS *m*/*z* 510 [M+ Na]+; HRESI(+)MS *m*/*z* 510.2099[M + Na]+ (calcd for C_28_H_29_N_3_O_5_Na, 510.1999).

### 3.10. Fractionation of a Scaled Up M1 Culture of Spiromastix sp. CMB-F455

The fungus CMB-F455 was inoculated on M1 agar (×100 plates) and incubated at 26.5 °C for 10 days, after which the combined agar and mycelia were harvested, extracted with EtOAc (4 × 600 mL), and the filtered organic phase concentrated in vacuo at 40 °C to yield an extract (350 mg). This extract was triturated with *n*-hexane to yield after in vacuo concentration *n*-hexane (50 mg) and MeOH (290 mg) solubles. With UPLC-QTOF analysis localising the target chemistry in the MeOH solubles, a portion (280 mg) was fractionated by preparative reversed phase HPLC (Phenomenex Luna-C_8_ 10 mm, 21.2 × 250 mm column, with a 20 min 20 mL/min gradient elution from 90% H_2_O/MeCN to MeCN with an isocratic 0.1% TFA/MeCN modifier) to further concentrate the target chemistry into two fractions. Fraction A (5.2 mg) was subjected to semi-preparative reversed phase HPLC (ZORBAX SB-C_3_ 5 μm, 9.4 × 250 mm column, with a 35 min 3 mL/min isocratic elution at 75% H_2_O/MeCN inclusive of a 0.1% TFA/MeCN modifier) to yield brasiliamide A (**8**) (1.0 mg, 0.3%). Fraction B (4.5 mg) was subjected to semi-preparative reversed phase HPLC (ZORBAX SB-C_3_ 5 μm, 9.4 × 250 mm column, with a 40 min 3 mL/min isocratic elution at 70% H_2_O/MeCN inclusive of a 0.1% TFA/MeCN modifier) to yield chrysosporazine D (**7**) (1.2 mg, 0.35%) (Figure S9 and Scheme S4). Note that all yields were estimated as that present in the unfractionated EtOAc extract.

*Chrysosporazine D* (**7**). Yellow oil; [α]_D_^22.4^ + 19.7 (*c* 0.086, MeOH); NMR (600 MHz, DMSO-*d*_6_) see Figure 3 and Figure S42; HRESI(+)MS *m*/*z* 509.2043 [M + H]+ (calcd for C_29_H_30_N_2_O_5_Na, 509.2047).

*Brasiliamide A* (**8**). Yellow oil; [α]_D_^22.2^–32.4 (*c* 0.0416, MeOH); NMR (600 MHz, DMSO-*d*_6_) see Table S6, Figure 2, Figures S43 and S44; ESI(+)MS *m*/*z* 461 [M + Na]+; HRESI(+)MS *m*/*z* 461.1690 [M+ Na]+ (calcd for C_24_H_26_N_2_O_6_Na, 461.1683).

### 3.11. Media MATRIX Cultivation Profiling of Spiromastix sp. CMB-F455

The fungus CMB-F455 was cultured in a 24-well microbioreactor format [22] using ×11 different culture media, across both static and shaken broths (1.5 mL) and solid agar (1 g slant), and extracted in situ with EtOAc (2 mL) to provide ×33 culture extracts. The culture extracts were individually decanted, filtered, dried under N_2_ and the resulting residue subjected to UPLC-DAD and UPLC-QTOF with an internal calibrant, and GNPS analysis, to detect and quantify levels of metabolite production (Table S2 and Figures S10–S12).

### 3.12. Precursor-Directed Biosynthesis Cultivation Profiling of Spiromastix sp. CMB-F455

The fungus CMB-F455 was cultured on individual M1 plates, each supplemented with 1, 2 and 4 mg/mL of the sodium salts of either nicotinic, isonicotinic, benzoic, picolinic, cinnamic, caffeic and coumaric acids. After 14 days the agar and mycelia from each plate were harvested, extracted with EtOAc (15 mL), and subjected to UPLC-DAD and UPLC-QTOF analysis. None of these precursor-directed biosynthesis experiments lead to the detection of new *chrysosporazines* (Figure S13).

### 3.13. Acid Hydrolysis and Chemical Correlation of ***2***, ***4***, ***6*** and ***7*** to the Common Product ***10***

Individual aliquots (0.1 mg) of **2**, **4**, **6** and **7** in 1 M HCl (0.3 mL) were heated to 100 °C, and samples (50 μL) taken at 12, 24 and 36 h were analysed by UPLC-DAD and UPLC-QTOF to detect the partially hydrolysed deacetylated analogues **2a**, **4a**, **6a** and **7a** and a common fully hydrolysed piperazine **10** [5] (Figure 7 and Figures S45–S48).

### 3.14. Antibacterial Assay

The bacterium to be tested was streaked onto a tryptic soy agar plate and incubated at 37 °C for 24 h. One colony was then transferred to fresh tryptic soy broth (5 mL) and the cell density was adjusted to 10^4^–10^5^ CFU/mL. Analytes (compounds) to be tested were dissolved in DMSO and diluted with H_2_O to a stock solution (600 μM in 20% DMSO), which was serially diluted to give concentrations ranging from 600 μM to 0.2 μM in 20% DMSO. An aliquot (10 mL) of each dilution was transferred to a 96-well microtiter plate, and freshly prepared microbial broth (190 mL) was added to each well to give final analyte concentrations ranging from 30 to 0.01 μM in 1% DMSO. Assay plates were incubated at 37 °C for 24 h and the optical density of each well measured spectrophotometrically at 600 nm using POLARstar Omega plate (BMG LABTECH, Offenburg, Germany). Each analyte was screened against the Gram-negative bacteria *Escherichia coli* ATCC 11,775 and the Gram +ve clinical isolate *Bacillus subtilis* ATCC 6633. Rifampicin, ampicillin, and methicillin were used as a positive control (30 μM in 1% DMSO). The IC_50_ value was calculated as the concentration of the analyte/control required for 50% inhibition of the bacterial cells using Prism 7.0 (GraphPad Software Inc., La Jolla, CA, USA) (Figure S49).

### 3.15. Antifungal Assay

The fungus *Candida albicans* ATCC 10,231 was streaked onto a tryptic soy agar plate and incubated at 37 °C for 48 h. One colony was then transferred to fresh tryptic soy broth (5 mL) and the cell density was adjusted to 10^4^–10^5^ CFU/mL. Analytes (compounds) to be tested were dissolved in DMSO and diluted with H_2_O to give a stock solution (600 μM in 20% DMSO), which was serially diluted with 20% DMSO to give concentrations from 600 μM to 0.2 μM in 20% DMSO. An aliquot (10 mL) of each dilution was transferred to a 96-well microtiter plate and freshly prepared fungal broth (190 μL) was added to each well to give final analyte concentrations ranging from 30 to 0.01 μM in 1% DMSO. The plates were incubated at 37 °C for 24 h and the optical density of each well measured spectrophotometrically at 600 nm using a POLARstar Omega plate (BMG LABTECH, Offenburg, Germany). Ketoconazole B was used as a positive control (30 μM in 1% DMSO). Where relevant, IC_50_ value were calculated as the concentration of the compound or antifungal drug required for 50% inhibition of the fungal cells using Prism 8.0 (GraphPad Software Inc., La Jolla, CA, USA) (Figure S49).

### 3.16. Cytotoxicity (MTT) Assay

Adherent drug sensitive human colorectal carcinoma (SW620) cells were cultured in Roswell Park Memorial Institute (RPMI) 1640 medium. All cells were cultured as adherent mono layers in flasks supplemented with 10% foetal bovine serum, L–glutamine (2 mM), penicillin (100 unit/mL) and streptomycin (100 μg/mL), in a humidified 37 °C incubator supplied with 5% CO_2_. Briefly, cells were harvested with trypsin and dispensed into 96-well microtiter assay plates at 4000 cells/well, after which they were incubated for 18 h at 37 °C with 5% CO_2_ (to allow cells to attach as adherent mono layers). Analytes (compounds) to be tested were dissolved in 20% DMSO in PBS (*v/v*), and aliquots (10 μL) were applied to cells over a series of final concentrations ranging from 10 nM to 30 μM. After 48 h incubation at 37 °C with 5% CO_2_, an aliquot (10 μL) of 3-(4,5-dimethylthiazol-2-yl)-2,5-diphenyltetrazolium bromide (MTT) in phosphate buffered saline (PBS, 5 mg/mL) was added to each well (final concentration 0.5 mg/mL), and microtiter plates were incubated for a further 4 h at 37 °C with 5% CO_2_. After final incubation, the medium was aspirated, and precipitated formazan crystals were dissolved in DMSO (100 μL/well). The absorbance of each well was measured at 600 nm with a POLARstar Omega plate (BMG LABTECH, Offenburg, Germany). Where relevant, IC_50_ values were calculated using Prism 8.0, as the concentration of analyte required for 50% inhibition of cancer cell growth (compared to negative controls). The negative control was 1% aqueous DMSO, while the positive control was doxorubicin (30 μM). All experiments were performed in duplicate from two independent cultures (Table S7 and Figure S50).

### 3.17. P-Glycoprotein Mediated MDR Reversal Assay

The assay is similar to the cytotoxicity (MTT) assay shown above. However, instead of measuring the cytotoxicity of analytes, this assay measures the ability of analytes to reverse P-gp mediated resistance to doxorubicin in MDR P-gp overexpressing human colorectal carcinoma (SW620 Ad300) cells. SW620 Ad300 cells were cultivated in flasks as adherent monolayers in RPMI medium supplemented with 10% foetal bovine serum, L–glutamine (2 mM), penicillin (100 unit/mL), streptomycin (100 μg/mL) and doxorubicin (300 ng/mL) in a humidified 37 °C incubator supplied with 5% CO_2_. The cells were passaged 5 times and were maintained in 300 ng/mL doxorubicin to confirm drug resistance status. On the day of the experiment, SW620 Ad300 cells were harvested with trypsin and dispensed into 96-well microtiter assay plates at 4000 cells/well in 190 μL medium per well, after which they were incubated for 48 h at 37 °C with 5% CO_2_. Following incubation, analytes (2.5 μM) were added to wells containing serial dilutions of doxorubicin (30 to 0.01 μM). After a further 48 h incubation at 37 °C with 5% CO_2_, a solution of 3-(4,5-dimethylthiazol-2-yl)-2,5-diphenyltetrazolium bromide (MTT) in phosphate buffered saline (PBS, 5 mg/mL) was added to each well (final concentration 0.5 mg/mL), and microtiter plates were incubated for a further 4 h at 37 °C with 5% CO_2_. After the media was carefully aspirated, the precipitated formazan crystals were dissolved in DMSO (100 μL) and the absorbance was measured at 600 nm with a POLARstar Omega plate (BMG LABTECH, Offenburg, Germany). Verapamil (2.5 μM) and DMSO served as positive and negative controls, respectively. All experiments were performed in duplicate from two independent cultures. During the course of study, each round of assay was validated against independent positive and negative controls, allowing for measures of fold of resistance (FR) and gain in sensitivity (GS). To compare results between separate rounds of assays, the fold increase (FI) was calculated as the ratio of the (analyte + doxorubicin GS)/(verapamil + doxorubicin GS) (Table 3 and Table S7 and Figure S50).

## Figures and Tables

**Table 1:** ^1^H NMR (DMSO-*d*_6_) data for **1**, **3** and **5**.

Position	(1) δ_H_, Multi (*J* in Hz)	(3) δ_H_, Multi (*J* in Hz)	(5) δ_H_, Multi (*J* in Hz)
1	*a.* 4.14, dd (13.8, 3.9)	*a.* 4.25, dd (13.3, 3.8)	*a.* 4.19, dd (14.5, 4.0)
	*b.* 2.94, m	*b.* 3.03, m	*b.* 2.98, m
2	3.84, ddd (10.6, 10.6, 3.9)	3.87, ddd (11.2, 8.8, 3.8)	3.92, ddd (14.5, 10.3, 3.7)
3	4.47, d (10.6)	4.56, d (8.8)	4.56, d (10.3)
5/9	7.35, m	7.26, m	7.40, m
6/8	7.44, m	7.35, m	7.46, m
7	7.37, m	7.29, m	7.40, m
1′	*a.* 4.57, dd (13.3, 1.2)	*a.* 4.56, m	*a.* 4.55, dd (13.4, 1.3)
	*b*. 2.95, m	*b.* 3.01, m	*b.* 2.99, m
2′	4.21, m	4.21, m	4.23, m
3′	*a.* 2.90, dd (13.5, 8.1)	*a.* 2.88, dd (13.5, 8.3)	*a.* 2.90, dd (13.4, 8.6)
	*b.* 2.86, dd (13.5, 6.6)	*b*. 2.83, dd (13.5, 6.3)	*b.* 2.85, dd (13.4, 6.4)
4′	-	-	-
5′	6.54, d (1.2)	6.54, d (1.3)	6.54, d (1.4)
9′	6.55, d (1.2)	6.55, d (1.3)	6.55, d (1.4)
4″	6.60, d (7.8)	-	7.89, s
5″	7.44, m	8.57, dd (4.7, 1.8)	-
6″	7.39, m	7.44, ddd (7.8, 4.7)	8.64, d (4.7)
7″	8.04, dd (7.7, 1.4)	8.34, dd (7.8, 1.8)	7.88, d (4.7)
1-NCOCH_3_	-	-	-
1-NCOCH_3_	1.70, s	1.68, s	1.70, s
6′-OCH_2_	5.94/5.93, ABq	5.95/5.93, ABq	5.95/5.94, ABq
8′-OCH_3_	3.79, s	3.78, s	3.78, s

**Table 2:** ^13^C NMR (DMSO-*d*_6_) data for **1**, **3** and **5**.

Position	(1) δ_C_, Type	(3) δ_C_, Type	(5) δ_C_, Type
1	40.1, CH_2_	40.4, CH_2_	40.1, CH_2_
2	57.8, CH	58.6, CH	58.2, CH
3	46.3, CH	48.8, CH	43.7, CH
4	140.4 ^A^, C	140.7, C	139.4, C
5/9	129.3, CH	129.3, CH	129.2^A^, CH
6/8	129.1, CH	128.6, CH	129.1^A^, CH
7	127.6, CH	127.1, CH	127.9, CH
1′	44.6, CH_2_	45.0, CH_2_	44.8, CH_2_
2′	54.6, CH	54.9, CH	54.6, CH
3′	34.9, CH_2_	35.0, CH_2_	34.8, CH_2_
4′	132.5, C	132.6, C	132.6, C
5′	103.3, CH	103.4, CH	103.3, CH
6′	148.3, C	148.2, C	148.3, C
7′	133.0, C	133.4, C	133.3, C
8′	143.0, C	143.1, C	143.0, C
9′	109.0, CH	109.1, CH	109.0, CH
1″	163.9, C	163.2, C	162.3, C
2″	127.4, C	122.9 ^A^, C	134.4, C
3″	140.3 ^A^, C	158.4, C	133.7, C
4″	126.9 ^B^, CH	-	148.6, CH
5″	132.3, CH	152.6, CH	-
6″	127.0 ^B^, CH	122.8 ^A^, CH	148.3, CH
7″	127.6, CH	135.6, CH	120.2, CH
1-NCOCH_3_	168.4, C	168.6, C	168.5, C
1-NCOCH_3_	20.8, CH_3_	20.8, CH_3_	20.8, CH_3_
6′-OCH_2_	101.0, CH_2_	101.0, CH_2_	101.0, CH_2_
8′-OCH_3_	56.2, CH_3_	56.3, CH_3_	56.2, CH_3_

^A,B^ assignments with the same superscript within a column are interchangeable.

**Table 3:** Ability of analytes **1**–**9** and **11**–**14** to reverse doxorubicin resistance in P-gp over-expressing human colorectal carcinoma cells (SW600 Ad300).

SW620 Ad300				
Treatment	IC_50_ (μM)	FR	GS	FI
doxorubicin	5.75	57.5	1.0	0.12
verapamil	>30	ND	ND	-
+ verapamil (2.5 μM)	0.71	7.1	8.1	1.00
+ chrysosporazine T (**1**)	0.97	9.7	5.9	0.61
+ chrysosporazine C (**9**)	0.31	3.1	18.5	2.28
+ chrysosporazine U (**2**)	2.76	27.6	2.0	0.25
+ chrysosporazine D (**7**)	4.36	43.6	1.32	0.16
+ azachrysosporazine T1 (**3**)	0.89	8.9	6.4	0.80
+ azachrysosporazine C1 (**11**)	0.27	2.7	21.3	2.63
+ azachrysosporazine U1 (**4**)	2.78	27.8	2.0	0.25
+ azachrysosporazine D1 (**12**)	3.55	35.5	1.6	0.20
+ neochrysosporazine R (**5**)	0.58	5.8	9.9	1.22
+ neochrysosporazine I (**13**)	1.01	10.1	5.7	0.70
+ neochrysosporazine S (**6**)	1.95	19.5	2.9	0.36
+ neochrysosporazine J (**14**)	6.18	61.8	0.9	0.11
+ brasiliamide A (**8**)	5.27	52.7	1.1	0.13

+ = doxorubicin supplemented with 2.5 μM analyte; FR = fold resistance determined as (analyte IC50 SW620 Ad300)/(analyte IC50 SW620); GS = gain in sensitivity ratio relative to doxorubicin control; FI = fold increase ratio as (analyte + doxorubicin GS)/(verapamil + doxorubicin GS).

## Data Availability

Data is contained within the article or Supplementary Materials, or from the authors on request.

## Supplementary Materials

The following supporting information can be downloaded at: https://www.mdpi.com/article/10.3390/molecules27103172/s1, and consists of fully tabulated 1D and 2D NMR data and spectra along with chemical degradation and biological activity data: Figures S1–S50, Tables S1–S7, Schemes S1–S4.

## Sample Availability

Samples of selected compounds may be available from the authors on request, subject to an approved Materials Transfer Agreement.
