# Supplementary material for: Chrysosporazines Revisited: Regioisomeric Phenylpropanoid Piperazine P-Glycoprotein Inhibitors from Australian Marine Fish-Derived Fungi
Source: Molecules. 2022 May 16;27(10):3172. doi: 10.3390/molecules27103172 (PMC9146134; doi:10.3390/molecules27103172)
Supplement: Supplementary file 1 [file molecules-27-03172-s001.zip › molecules-1719586-supplementary.pdf]

Supplementary Material

Chrysosporazines Revisited: Regioisomeric phenylpropanoid piperazine P-glycoprotein inhibitors from Australian marine fish-derived fungi.

Amila Agampodi Dewa, Zeinab G. Khalil, Ahmed elbanna and Robert J. Capon*

Institute for Molecular Bioscience, The University of Queensland, St Lucia, QLD, Australia

*** Correspondence:** Professor Robert Capon

Tel +61 7 3346 2979. Facsimile +61 7 3346 2090. Email: r.capon@uq.edu.au

Table of content

Table of content ... ii

List of figures ... iv

List of Tables ... vii

List of Schemes ... viii

1 Fungal Isolation and phylogenetic tree ... 1

2 Cultivation of *Aspergillus* sp. CMB-F661, production and isolation of chrysosporazine T and U (1-2) ... 3

3 *Aspergillus* sp. CMB-F661 media MATRIX study ... 4

4 Analytical scale precursor-directed feeding studies for CMB-F455 ... 7

5 Scaled-up cultivation of CMB-F661 with sodium nicotinate ... 7

6 Scaled-up cultivation of CMB-F661 with sodium isonicotinate ... 8

7 Identification of natural azachrysosporazine T1 and U1 ... 8

8 Chemical analysis of *Spiromastix* sp. CMB-F455 ... 9

9 MATRIX study for *Spiromastix* sp. CMB-F455 ... 10

10 Precursor directed biosynthesis for *Spiromastix* sp. CMB-F455 ... 12

11 Chrysosporazine T (1) ... 13

12 Chrysosporazine U (2) ... 18

13 Azachrysosporazine T1 (3) ... 19

14 Azachrysosporazine U1 (4) ... 24

15 Neochrysosporazine R (5) ... 25

16 Neochrysosporazine S (6) ... 30

17 Chrysosporazine D (7) ... 31

18 Brasiliamide A (8) ... 32

19 Acid hydrolysis of chrysosporazine U (2) ... 34

20 Acid hydrolysis of azachrysosporazine U1 (4) ... 34

21 Acid hydrolysis of neochrysosporazine S (6) 35
22 Antimicrobial Activity 36
23 Cytotoxicity and P-gp activity 36

List of figures

Figure S1. (a) CMB-F661, (b) CMB-F455 fungal strains cultured in PD agar plate 1

Figure S2. Phylogenetic tree by PhyML Maximum Likehood analysis of 18s rRNA sequences showing the relationship of CMB-F214, CMB-F294, CMB-F661 and CMB-F455 among selected other reference strains 1

Figure S3. HPLC-DAD profile at 210 nm of CMB-F661 PDA extract and UV-vis spectra of metabolites 3

Figure S4. CMB-F661 on different culture conditions (MATRIX) (a) liquid shaking; (b) liquid static and (c) solid agar 4

Figure S5. UPLC-DAD (210 nm) chromatograms for cultivation of CMB-F661 on different culture conditions (MATRIX) and production of chrysosporazines T–U (1–2) (red peaks), across different media: (A) M1; (B) M2; (C) ISP-2; (D) IMA; (E) CG; (F) TS; (G) YEME; (H) YES; (I) PD; (J) PYG; (K) SD, each media under different conditions: (i) solid agar; (ii) static broth; (iii) shaken broth, (iv) media blank, *internal calibrant. 5

Figure S6: GNPS molecular networking analysis of selected *Aspergillus* sp. CMB-F661media MATRIX cultivation extracts in solid agar condition; CGA and/or IMA (pink); YEME and/or YES (orange); M1 and/or M2 (green); PDA and/or PYG (purple); and ISP2 and/or SDA (blue). 6

Figure S7. UPLC-DAD (210 nm) of CMB-F661 cultivated in PDA media in the presence of (a) sodium nicotinate (2mg/mL); (b) sodium isonicotinate (2mg/mL); (c) sodium benzoate (2mg/mL); (d) sodium picolinate (2mg/mL); (e) CMB-F214 control (red peaks represent the predicted new unnatural chrysosporazines, blue peaks represent the chrysosporazine T (1) and U (2).. 7

Figure S8: UPLC-QTOF-SIE analysis of an PDA agar culture of CMB-F661 (without sodium nicotinate feeding), (a) CMB-F661 PDA agar culture; (b) SIE at *m/z* 486; (c) purified azachrysosporazine T1 (3); (d) purified neochrysosporazine R (5); (e) purified chrysosporazine T (1); (f) SIE at *m/z* 488; (g) purified azachrysosporazine U1 (4); (h) purified neochrysosporazine S (6); (i) purified chrysosporazine U (2) 8

Figure S9. GNPS molecular networking and HPLC-DAD (210 nm) analysis of a M1 solid phase cultivation of *Spiromastix* sp. CMB-F455.. 9

Figure S10. Global natural Product Social (GNPS) molecular networking cluster for chrysosporazines; CMB-F214 pink nodes, CMB-F294 orange nodes, CMB-F661 green nodes, CMB-F455 purple nodes 10

Figure S11. CMB-F455 on different culture conditions (MATRIX) (a) liquid shaking; (b) liquid static and (c) solid agar 10

Figure S12. UPLC-DAD (210 nm) chromatograms for cultivation of CMB-F455 on different culture conditions (MATRIX) and production of brasiliamide A (8) (red peak), across different media: (A) M1; (B) M2; (C) ISP-2; (D) IMA; (E) CG; (F) TS; (G) YEME; (H) YES; (I) PD; (J) PYG; (K) SD, each media under different conditions: (i) solid agar; (ii) static broth; (iii) shaken broth, (iv) media blank, *internal calibrant 11

Figure S13. UPLC-DAD (210 nm) of CMB-F455 cultivated in M1 media in the presence and absence of sodium salts of different acids at 2mg/mL (i) benzoic acid derivatives (a) CMB-F455 control; (b) picolinic acid; (c) isonicotinic acid; (d) nicotinic acid; (e) benzoic acid; (f) M1 media blank; (ii) cinnamic acid derivatives (a) CMB-F455 control; (g) coumaric acid; (h) caffeic acid; (i) cinnamic acid; (f) M1 media blank; *internal calibrant 12

Figure S14. ^{1}H NMR (600 MHz, DMSO-d_6) spectrum for chrysosporazine T (1) 14

Figure S15. Comparison of ^{1}H NMR (600 MHz, DMSO-d_6) spectra of (a) chrysosporazine C (7) and (b) chrysosporazine T (1) 14

Figure S16. ^{13}C NMR (150 MHz, DMSO-d_6) spectrum for chrysosporazine T (1) 15

Figure S17. Expanded HSQC NMR (600 MHz, DMSO-d_6) spectrum (part 1) for chrysosporazine T (1), major rotamer (labelled black); minor rotamer (labelled green) 15

Figure S18. Expanded HSQC NMR (600 MHz, DMSO-d_6) spectrum (part 2) for chrysosporazine T (1), major rotamer (labelled black); minor rotamer (labelled green) 16

FigureS19. HMBC NMR (600 MHz, DMSO-d_6) spectrum for chrysosporazine T (1) 16

Figure S20. COSY NMR (600 MHz, DMSO-d_6) spectrum for chrysosporazine T (1) 17

Figure S21. ROESY NMR (600 MHz, DMSO-d_6) spectrum for chrysosporazine T (1), major rotamer (labelled black); minor rotamer (labelled green) 17

Figure S22. ^{1}H NMR (DMSO-d_6) spectrum for chrysosporazine U (2) 18

Figure S23. ^{1}H NMR (600 MHz, DMSO-d_6) spectrum for a) chrysosporazine T (1), b) chrysospirazine D (7) and chrysosporazine U (2) 18

Figure S24. ^{1}H NMR (600 MHz, DMSO-d_6) spectrum for azachrysosporazine T1 (3) 20

Figure S25. ^{13}C NMR (150 MHz, DMSO-d_6) spectrum for azachrysosporazine T1 (3) 20

Figure S26. Expanded HSQC NMR (600 MHz, DMSO-d_6) spectrum (part 1) for azachrysosporazine T1 (3), major rotamer (labelled black); minor rotamer (labelled green). 21

Figure S27. Expanded HSQC NMR (600 MHz, DMSO-d_6) spectrum (part 2) for azachrysosporazine T1 (3), major rotamer (labelled black); minor rotamer (labelled green). 21

Figure S28. HMBC NMR (600 MHz, DMSO-d_6) spectrum for azachrysosporazine T1 (3) .. 22

Figure S29. COSY NMR (600 MHz, DMSO-d_6) spectrum for azachrysosporazine T1 (3) ... 22

Figure S30. ROESY NMR (600 MHz, DMSO-d_6) spectrum for azachrysosporazine T1 (3), major rotamer (labelled black); minor rotamer (labelled green) 23

Figure S31. ^{1}H NMR (DMSO-d_6) spectrum for azachrysosporazine U1 (4) 24

Figure S32. ^{1}H NMR (600 MHz, DMSO-d_6) spectrum for a) chrysosporazine U (2), b) azachrysospirazine U1 (4) 24

Figure S33. ^{1}H NMR (600 MHz, DMSO-d_6) spectrum for neochrysosporazine R (5).......... 26

Figure S34. ^{13}C NMR (150 MHz, DMSO-d_6) spectrum for neochrysosporazine R (5).......... 26

Figure S35. Expanded HSQC NMR (600 MHz, DMSO-d_6) spectrum (part 1) for neochrysosporazine R (5), major rotamer (labelled black); minor rotamer (labelled green) .. 27

Figure S36. Expanded HSQC NMR (600 MHz, DMSO-d_6) spectrum (part 2) for neochrysosporazine R (5), major rotamer (labelled black); minor rotamer (labelled green) .. 27

Figure S37. HMBC NMR (600 MHz, DMSO-d_6) spectrum for neochrysosporazine R (5) ... 28

Figure S38. COSY NMR (600 MHz, DMSO-d_6) spectrum for neochrysosporazine R (5) 28

Figure S39. ROESY NMR (600 MHz, DMSO-d_6) spectrum for neochrysosporazine R (5), major rotamer (labelled black); minor rotamer (labelled green) 29

Figure S40. ^{1}H NMR (DMSO-d_6) spectrum for neochrysosporazine S (6) 30

Figure S41. ^{1}H NMR (600 MHz, DMSO-d_6) spectrum for a) chrysosporazine U (2), b) neochrysosporazine S (6).. 30

Figure S42. ^{1}H NMR (600 MHz, DMSO-d_6) spectrum for (a) known chrysosporazine D (7), (b) CMB-F455 isolated (7) 31

Figure S43. ^{1}H NMR ($CDCl_3$) spectrum for brasiliamide A (8) 33

Figure S44. ^{13}C NMR ($CDCl_3$) spectrum for brasiliamide A (8) 33

Figure S45. UPLC-DAD (210 nm) of acid hydrolysis of chrysosporazine U (2). (a) purified 2; acid hydrolysis of 2 at (b) 12 hr, (c) 24 hr and (d) 36 hr; (e) purified 10 34

Figure S46. UPLC-DAD (210 nm) of acid hydrolysis of azachrysosporazine U1 (4). (a) purified 4; acid hydrolysis of 4 at (b) 12 hr, (c) 24 hr and (d) 36 hr; (e) purified 10 34

Figure S47. UPLC-DAD (210 nm) of acid hydrolysis of neochrysosporazine S (6). (a) purified 6; acid hydrolysis of 6 at (b) 12 hr, (c) 24 hr and (d) purified 10 35

Figure S48: Comparison of UPLC-DAD retention times for 12hr hydrolysis products of (a) chrysosporazine U (2); (b) chrysosporazine D (7); (c) azachrysosporazine U1 (4); (d) azachrysosporazine D1 (12); (e) neochrysosporazine S (6); (f) neochrysosporazine J (14) ... 35

Figure S49. Growth inhibitory activity of chrysosporazines 1–9 36

Figure S50. (A) cytotoxicity of chryspsporazines (1–14), doxorubicin and verapamil against SW620. (B) effect of chrysosporazines or verapamil (2.5 μM) on the sensitivity of P-gp overexpressing SW620 Ad300 cancer cells to doxorubicin. 48 h MTT cytotoxicity assay was performed with a series of concentrations of doxorubicin (30 – 0.01 μM) on P-gp overexpressing SW620 Ad300 in the presence and absence of verapamil (2.5 μM) or 2.5 μM chrysosporazines (1–4). Data points are the means of ± SEM of duplicate determination from two independent cultures. 36

List of Tables

Table S1. Compositions of solid and liquid-based media used in MATRIX study 4

Table S2. Compounds corresponds to the cluster nodes in GNPS molecular networking for CMB-F455 10

Table S3. 1D and 2D NMR (600 MHz, DMSO-d_6) data for chrysosporazine T (1) (major rotamer) 13

Table S4. 1D and 2D NMR (600 MHz, DMSO-d_6) data for azachrysosporazine T1 (3) (major rotamer) 19

Table S5. 1D and 2D NMR (600 MHz, DMSO-d_6) data for neochrysosporazine R (5) (major rotamer) 25

Table S6. 1D and 2D NMR (600 MHz, $CDCl_3$) data for brasiliamide A (8) 32

Table S7. Effect of chrysosporazines (1–14) on inhibition of P-gp mediated resistance to doxorubicin in SW620 Ad300 and cytotoxicity against susceptible SW620 37

List of Schemes

Scheme S1. Isolation scheme of chrysosporazine T and U (1–2): a) trituration of crude extract with *n*-hexane (-1) and DCM (-2); b) preparative HPLC fractionation for 400 mg; c) semi-preparative HPLC purification 3

Scheme S2: Isolation scheme of azachrysosporazine T1 and U1(3–4): a) trituration of crude extract with *n*-hexane (-1) and DCM (-2); b) preparative HPLC fractionation for 380 mg; c) semi-preparative HPLC purification 7

Scheme S3: Isolation scheme of neochrysosporazine R–S (5–6): a) trituration of crude extract with *n*-hexane (-1) and DCM (-2); b) preparative HPLC fractionation for 500 mg; c) semi-preparative HPLC purification 8

Scheme S4. Isolation scheme of chrysosporazine D (7) and brasiliamide A (8): a) trituration of crude extract with *n*-hexane (-1) and MeOH (-2); b) preparative HPLC fractionation for 280 mg; c) semi-preparative purification 9

1 Fungal Isolation and phylogenetic tree

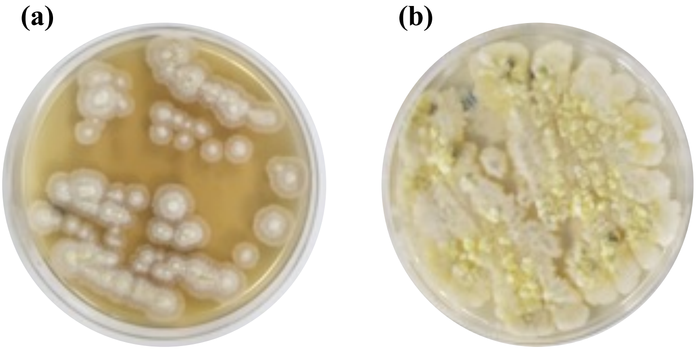

Figure S1. (a) CMB-F661, (b) CMB-F455 fungal strains cultured in PD agar plate

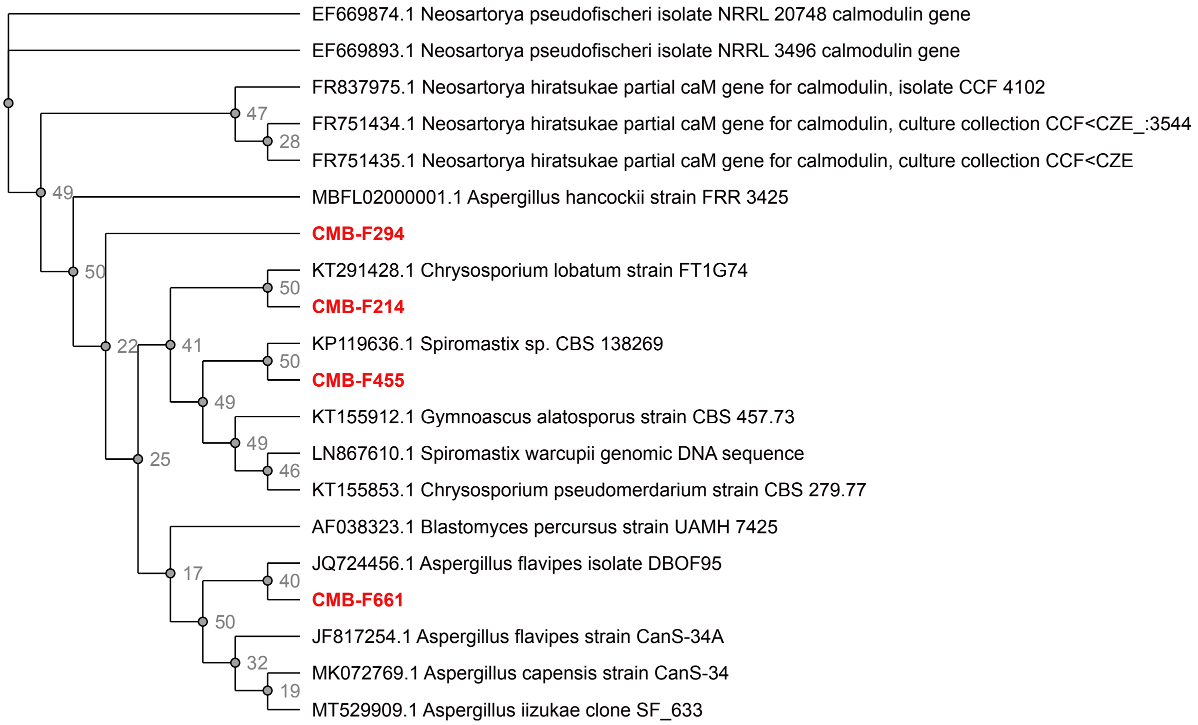

Figure S2. Phylogenetic tree by PhyML Maximum Likehood analysis of 18s rRNA sequences showing the relationship of CMB-F214, CMB-F294, CMB-F661 and CMB-F455 among selected other reference strains

ITS gene sequence for CMB-F661

GGGTCTCGTGGCCCAACCTCCCACCCGTGACTACTGTACCACTGTTGCTTCGGCG
GGCCCGCCAGCGTCCGCTGGCCGCCGGGGGGCTTCTGCCCCCGGGCCCGTGCCC
GCCGGAGACCCCAACACGAACACTGTTTCTGAAAGCCTGTATGAATCCGATTCTT
TGTAATCAGTTAAAACTTTCAACAATGGATCTCTTGGTTCCGGCATCGATGAAGA
ACGCAGCGAAATGCGATAACTAATGTGAATTGCAGAATTCAGTGAATCATCGAG
TCTTTGAACGCACATTGCGCCCCCTGGTATTCCGGGGGGCATGCCTGTCCGAGCG
TCATTACTGCCCTCAAGCCCGGCTTGTATTGGGTCCTCGTCCCCCTCCCCGGGGG
ACGGGCCCGAAAGGCAGCGGCGGCACCGCGTCCGGTCCTCGAGCGTATGGGGCT
TTGTCACCCGCTCTGTAGGCCCGGCCGGCGCCAGCCCACGCAACACCTTTTTTTT
CAGGTTGACCTCGGATCAGGTAGGGATACCCGCTGAACTTAAGCATATCAATAA
GGCGGAGGAA

ITS gene sequence of CMB-F455

GCGCGGTCGCCGGCGGCTCCCTCTCCGGGGGGTTCGTTTCGGCGCCGCGTCCGGC
CCAACCGTGTCTATCTGTACCTGTTGCTTCGGCGGGCCTGCGGGCCTCGCTCGCT
GCCGGGGGCCCCCTGGGGCTCCGGGCTCGTGCCCGCCGGAGACACCTGGAACTA
CTGTCGAAGTTGGCGGTCTGAGTAAACTTGATAATCATCAAAACTTTCAACAACG
GATCTCTTGGTTCCGGCATCGATGAAGAACGCAGCGAAATGCGATAAGTAATGT
GAATTGCAGAATTCCGTGAATCATCGAATCTTTGAACGCACATTGCGCCCCCTGG
TATTCCGGGGGGCATGCCTGTCCGAGCGTCATTGCAACCCCTTCAAGCCCGGCTT
GTGTGTTGGGCGTCGTCCCCGCTGGACGCGCCCGAAAGGCAGTGGCGGCTCCGT
GTCCGGTGCCCGAGCGTATGGGCTTTATCACCCGCTCCAGAGGCCCGGCCGGCGC
TGGCCCCGCGAGCCTTGACTGAACTCCAGTTAAGGTCTCAACTAAAACTTTTCGT
GGTTGACCTCGGATCAGGTAGGGATACCCGCTGAACTTAAGCATATCAATAA

2 Cultivation of *Aspergillus* sp. CMB-F661, production and isolation of chrysosporazine T and U (1-2)

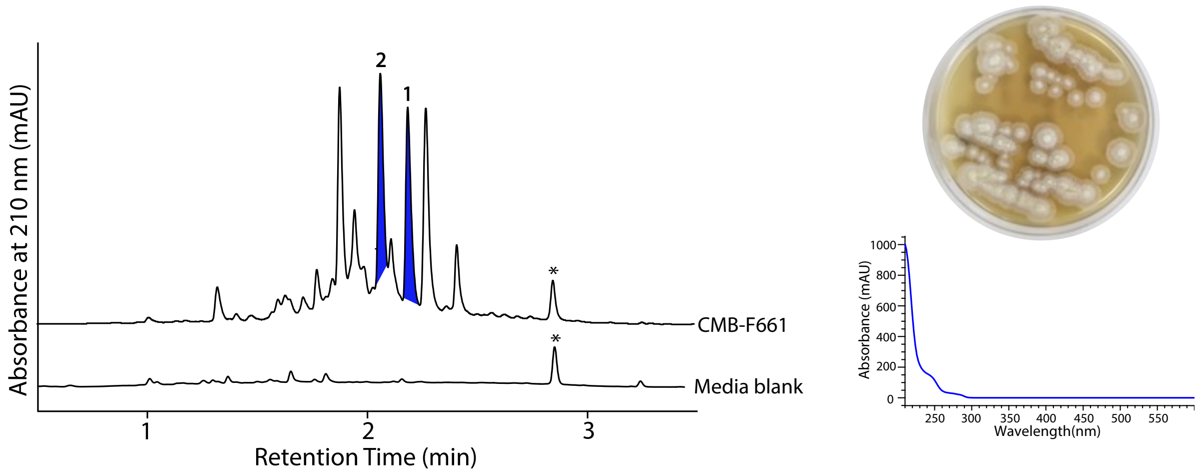

Figure S3. HPLC-DAD profile at 210 nm of CMB-F661 PDA extract and UV-vis spectra of metabolites

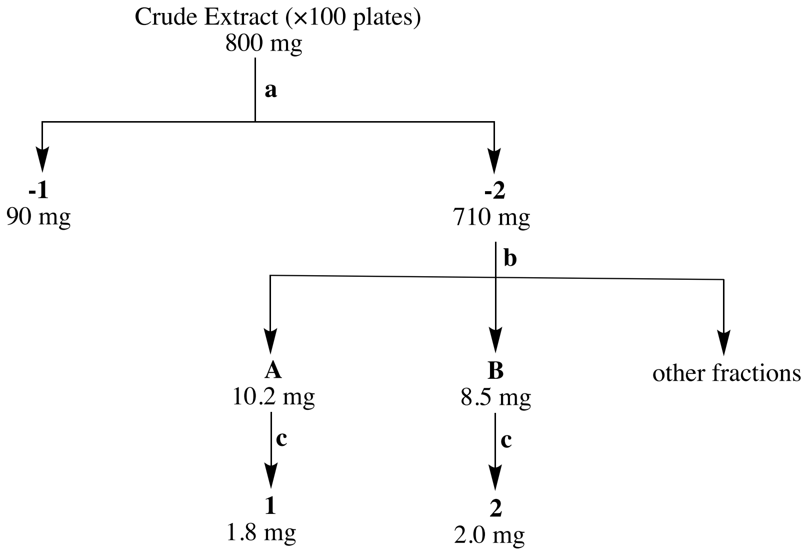

Scheme S1. Isolation scheme of chrysosporazine T and U (**1**–**2**): a) trituration of crude extract with *n*-hexane (-1) and DCM (-2); b) preparative HPLC fractionation for 400 mg; c) semi-preparative HPLC purification

3 *Aspergillus* sp. CMB-F661 media MATRIX study

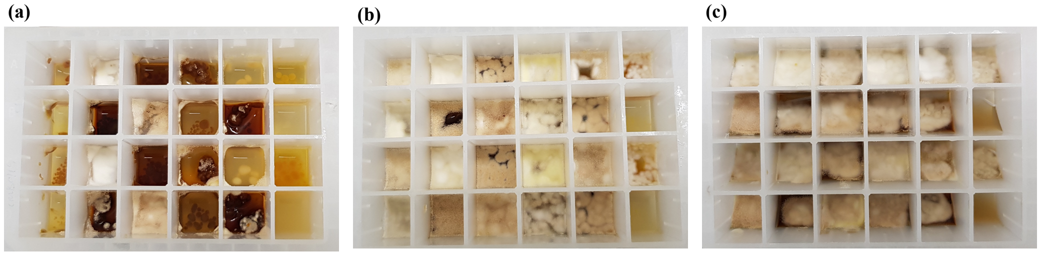

Figure S4. CMB-F661 on different culture conditions (MATRIX) (a) liquid shaking; (b) liquid static and (c) solid agar

Table S1. Compositions of solid and liquid-based media used in MATRIX study

Media	Composition (per Litre)
M1	Peptone (2.0 g), Yeast extract (4.0 g), Starch (10.0 g)
M2	Mannitol (40.0 g), Maltose (40.0 g), Yeast extract (10.0 g), K_2HPO_4 (2.0 g), $MgSO_4$. $7H_2O$ (0.5 g), $FeSO_4.7H_2O$ (0.01 g)
ISP-2	Yeast extract (4.0 g), Malt extract (10.0 g), Glucose (4.0 g)
IMA	Yeast extract (4 g), Malt extract (10 g), Glucose (4 g), Mannitol (40 g)
CGA	Glycerol (30 g), Casein peptone (Amyl) (2 g), K_2HPO_4 (1 g), K_2HPO_4 (1 g), NaCl (1 g), $MgSO_4.7H_2O$ (0.5 g), Trace element solution (5 mL)*<br>*Trace element solution,<br>$CaCl_2.2H_2O$ (3 g), $FeC_6O_7H_5$ (1 g), $MnSO_4$ (0.2 g), $ZnCl_2$ (0.1 g), $CuSO4.5H_2O$ (0.025 g), $Na_2B_4O_7.10H_2O$ (0.02 g), $CoCl_2$ (0.004 g), $Na_2MoO_4.2H_2O$ (0.01 g), Distilled H_2O (1000 mL), Filter sterilize
YEME	Yeast extract (3 g), Peptone (5 g), Malt extract (3 g), Glucose (10 g), Sucrose (170 g)
YES	Sucrose (150 g), Yeast extract (20 g), MgSO4.7H2O (0.5 g), ZnSO4.7H2O (0.01 g), CuSO4.5H2O (0.005 g)
PDA	Potato extract (4.0 g), Dextrose (20.0 g)
TSA	Pancreatic digest of casein (15.0 g), Peptic digest of soybean (5.0 g), NaCl (5.0 g)
SDA	Peptic digest of animal tissue (5.0 g), Pancreatic digest of casein (5.0 g), Dextrose (40.0 g)
PYG	Peptone (10 g), Yeast Extract (5 g) Dextrose (20 g)
ISP-4	Soluble starch (10.0 g), $CaCO_3$ (2.0 g), $(NH_4)_2SO_4$ (2.0 g), K_2HPO_4 (1.0 g), $MgSO_4.7H_2O$ (1.0 g), NaCl (1.0 g), $FeSO_4.7H_2O$ (1 mg), $MnCl_2.7H_2O$ (1.0 mg), $ZnSO_4.7H_2O$ (1.0 mg)

For solid agar 18 g/L of agar was added in each case

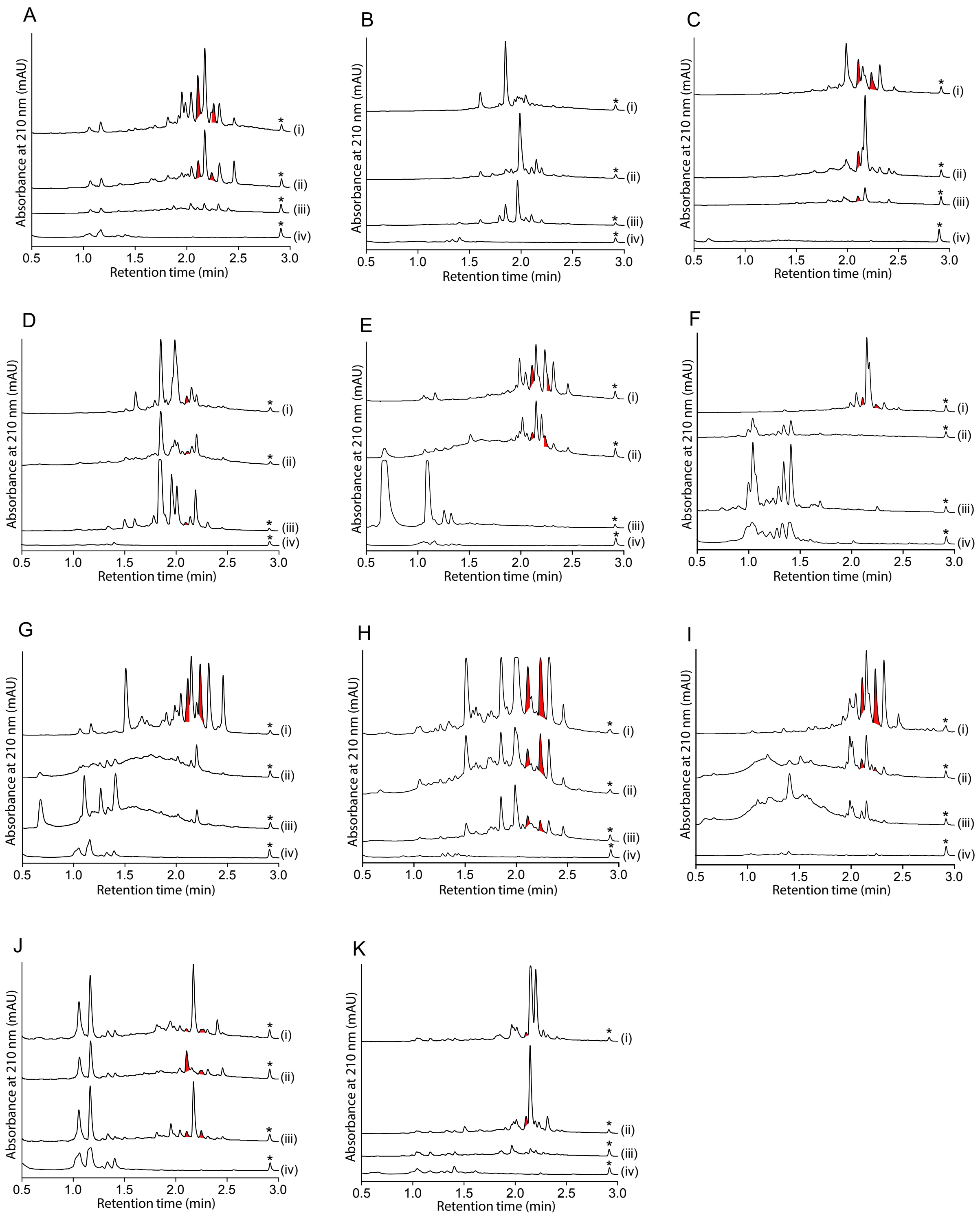

Figure S5. UPLC-DAD (210 nm) chromatograms for cultivation of CMB-F661 on different culture conditions (MATRIX) and production of chrysosporazines T–U (**1**–**2**) (red peaks), across different media: (A) M1; (B) M2; (C) ISP-2; (D) IMA; (E) CG; (F) TS; (G) YEME; (H) YES; (I) PD; (J) PYG; (K) SD, each media under different conditions: (i) solid agar; (ii) static broth; (iii) shaken broth, (iv) media blank, *internal calibrant.

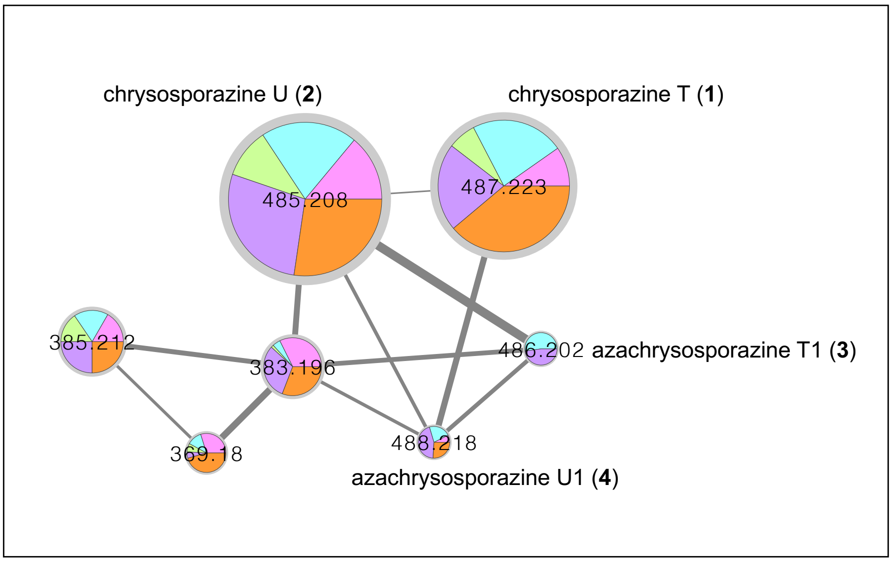

Figure S6: GNPS molecular networking analysis of selected *Aspergillus* sp. CMB-F661media MATRIX cultivation extracts in solid agar condition; CGA and/or IMA (pink); YEME and/or YES (orange); M1 and/or M2 (green); PDA and/or PYG (purple); and ISP2 and/or SDA (blue).

4 Analytical scale precursor-directed feeding studies for CMB-F455

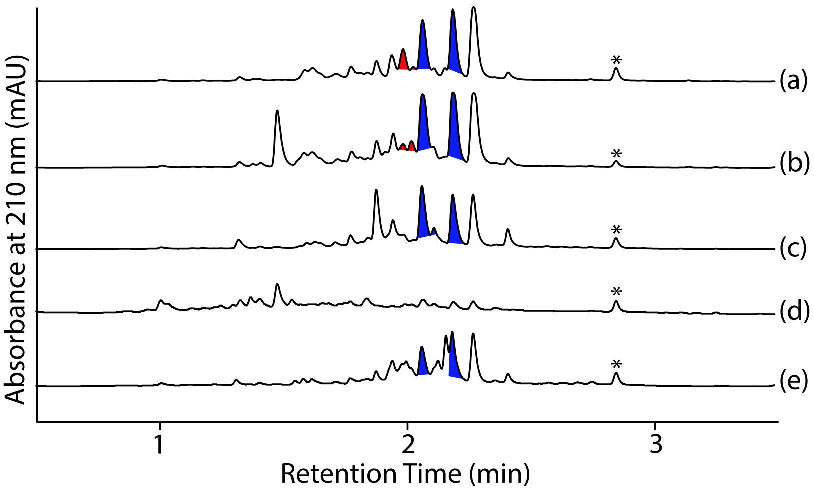

Figure S7. UPLC-DAD (210 nm) of CMB-F661 cultivated in PDA media in the presence of (a) sodium nicotinate (2mg/mL); (b) sodium isonicotinate (2mg/mL); (c) sodium benzoate (2mg/mL); (d) sodium picolinate (2mg/mL); (e) CMB-F214 control (red peaks represent the predicted new unnatural chrysosporazines, blue peaks represent the chrysosporazine T (**1**) and U (**2**)

5 Scaled-up cultivation of CMB-F661 with sodium nicotinate

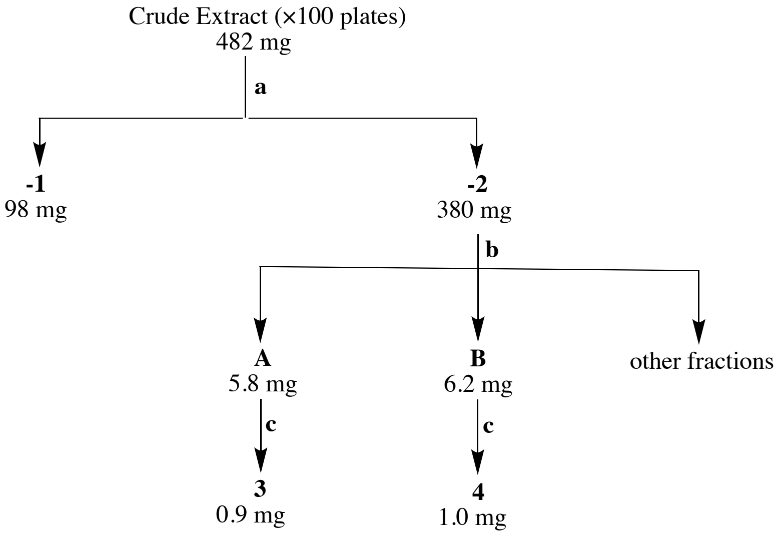

Scheme S2: Isolation scheme of azachrysosporazine T1 and U1(**3**–**4**): a) trituration of crude extract with *n*-hexane (-1) and DCM (-2); b) preparative HPLC fractionation for 380 mg; c) semi-preparative HPLC purification

6 Scaled-up cultivation of CMB-F661 with sodium isonicotinate

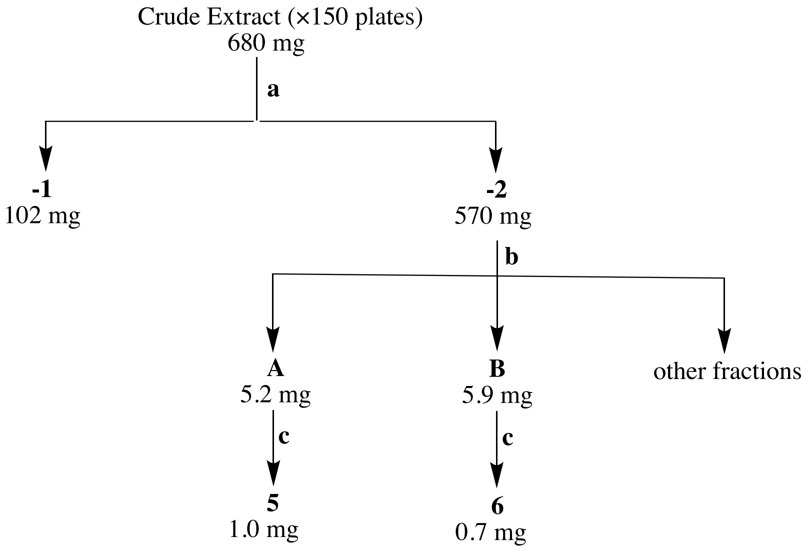

Scheme S3: Isolation scheme of neochrysosporazine R–S (**5**–**6**): a) trituration of crude extract with *n*-hexane (-1) and DCM (-2); b) preparative HPLC fractionation for 500 mg; c) semi-preparative HPLC purification

7 Identification of natural azachrysosporazine T1 and U1

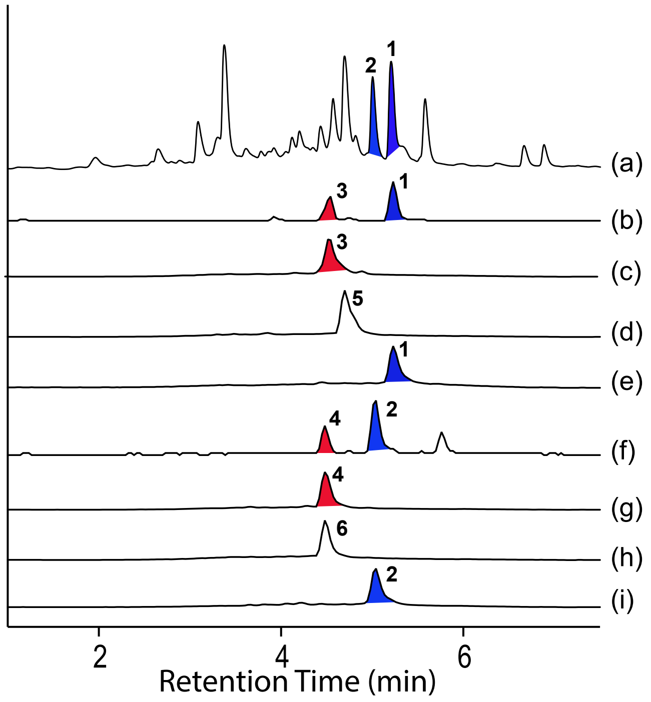

Figure S8: UPLC-QTOF-SIE analysis of an PDA agar culture of CMB-F661 (without sodium nicotinate feeding), (a) CMB-F661 PDA agar culture; (b) SIE at *m/z* 486; (c) purified azachrysosporazine T1 (**3**); (d) purified neochrysosporazine R (**5**); (e) purified chrysosporazine T (**1**); (f) SIE at *m/z* 488; (g) purified azachrysosporazine U1 (**4**); (h) purified neochrysosporazine S (**6**); (i) purified chrysosporazine U (**2**)

8 Chemical analysis of *Spiromastix* sp. CMB-F455

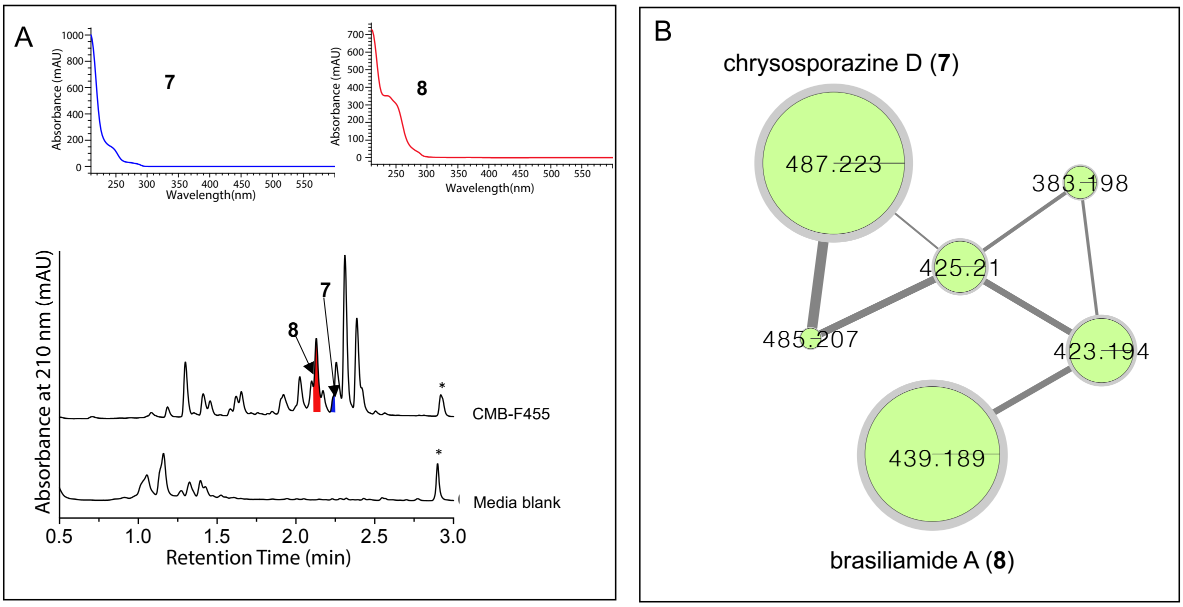

Figure S9. GNPS molecular networking and HPLC-DAD (210 nm) analysis of a M1 solid phase cultivation of *Spiromastix* sp. CMB-F455

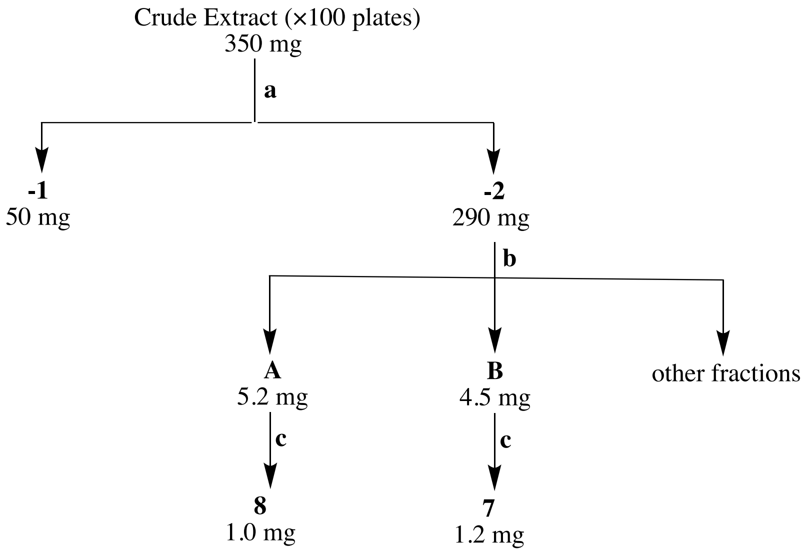

Scheme S4. Isolation scheme of chrysosporazine D (**7**) and brasiliamide A (**8**): a) trituration of crude extract with *n*-hexane (-1) and MeOH (-2); b) preparative HPLC fractionation for 280 mg; c) semi-preparative purification

9 MATRIX study for *Spiromastix* sp. CMB-F455

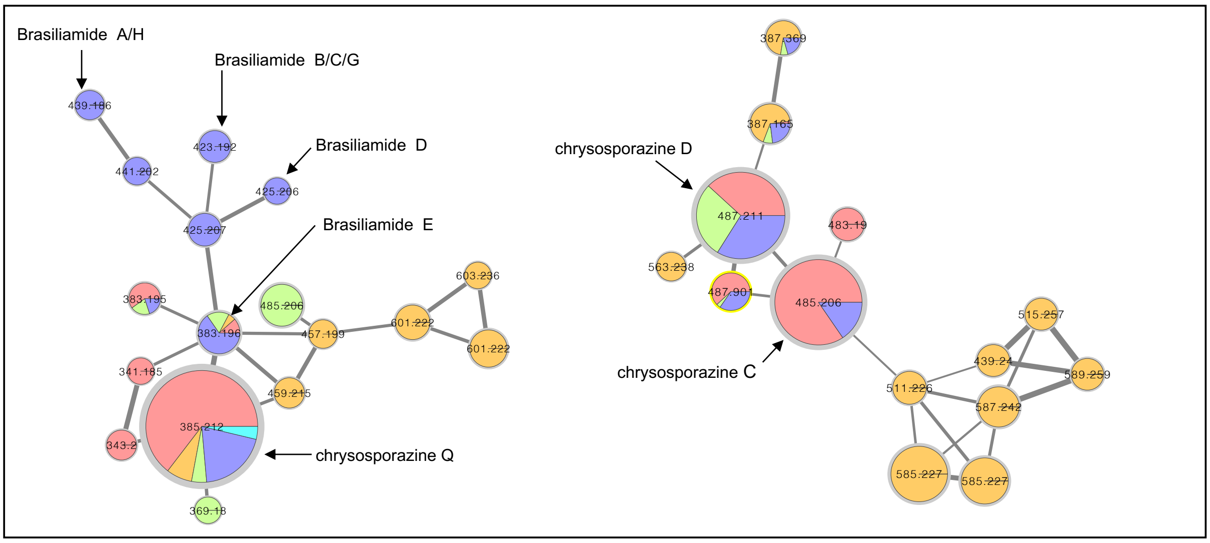

Figure S10. Global natural Product Social (GNPS) molecular networking cluster for chrysosporazines; CMB-F214 pink nodes, CMB-F294 orange nodes, CMB-F661 green nodes, CMB-F455 purple nodes

Table S2. Compounds corresponds to the cluster nodes in GNPS molecular networking for CMB-F455

HRESI *m/z* $(M+H)^+$	Suggested formulae	DBE	ΔmDa	Possible fungal hits
439.1815	$C_{24}H_{26}N_2O_6$	13	+2.31	brasiliamide A, brasiliamide H
423.1894	$C_{24}H_{26}N_2O_5$	13	+3.34	brasiliamide B, brasiliamide C, brasiliamide G
425.1998	$C_{24}H_{28}N_2O_5$	12	–2.15	brasiliamide D
383.1998	$C_{22}H_{26}N_2O_4$	11	+2.01	brasiliamide E
485.2011	$C_{29}H_{28}N_2O_5$	17	+3.12	chrysosporazine C
487.2099	$C_{29}H_{30}N_2O_5$	16	+2.22	chrysosporazine D
385.2099	$C_{22}H_{28}N_2O_4$	10	+3.41	chrysosporazine Q

(a) (b) (c)

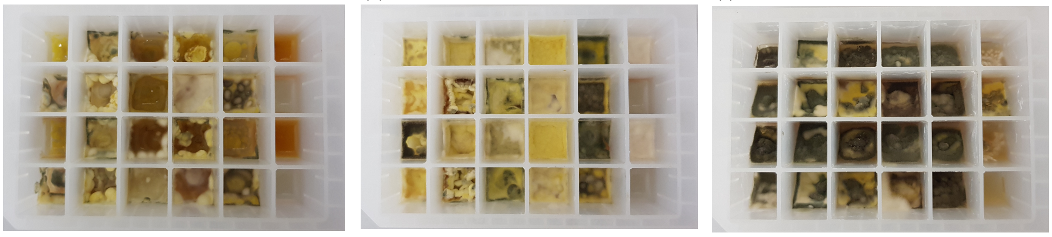

Figure S11. CMB-F455 on different culture conditions (MATRIX) (a) liquid shaking; (b) liquid static and (c) solid agar

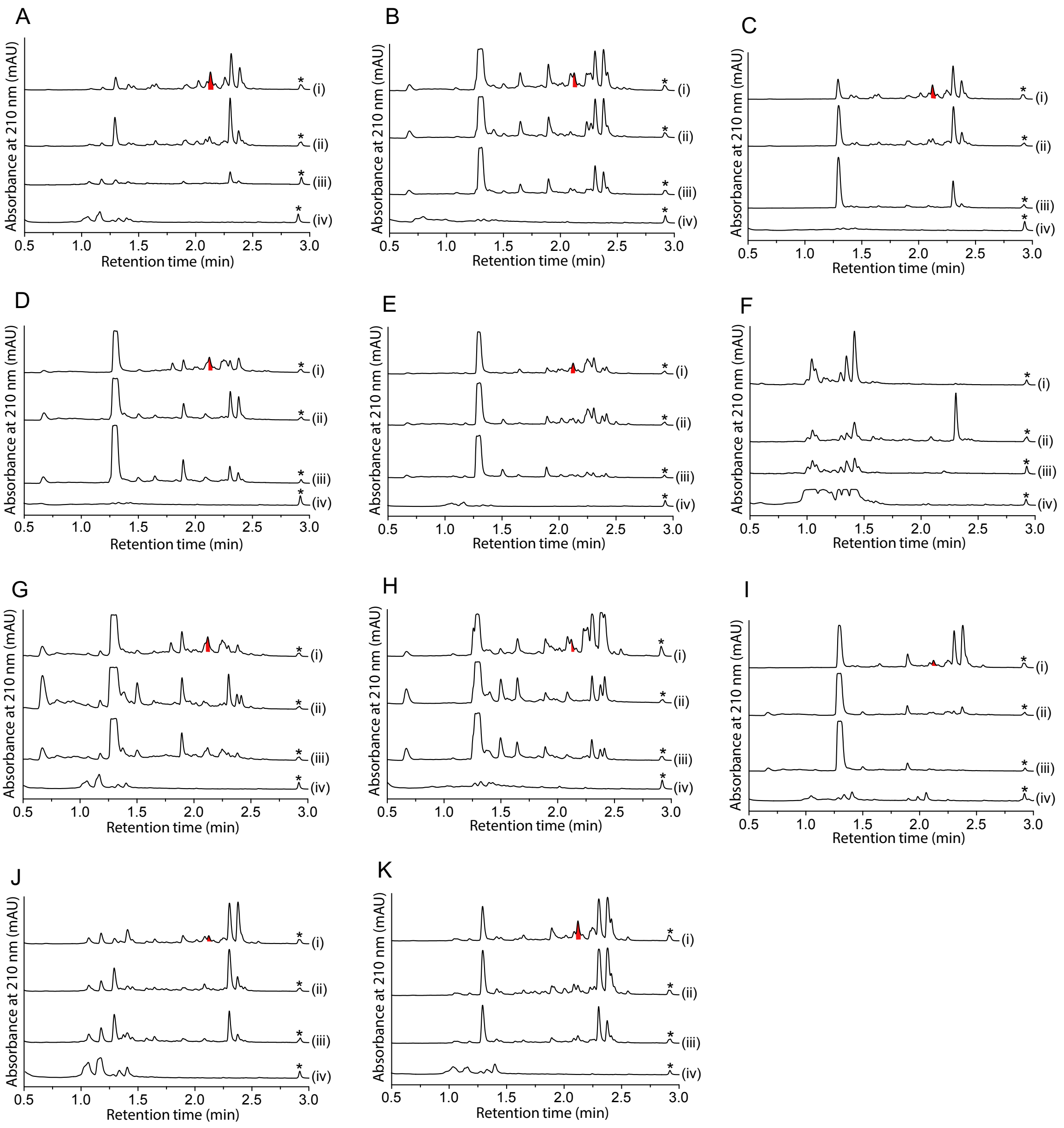

Figure S12. UPLC-DAD (210 nm) chromatograms for cultivation of CMB-F455 on different culture conditions (MATRIX) and production of brasiliamide A (**8**) (red peak), across different media: (A) M1; (B) M2; (C) ISP-2; (D) IMA; (E) CG; (F) TS; (G) YEME; (H) YES; (I) PD; (J) PYG; (K) SD, each media under different conditions: (i) solid agar; (ii) static broth; (iii) shaken broth, (iv) media blank, *internal calibrant

10 Precursor directed biosynthesis for *Spiromastix* sp. CMB-F455

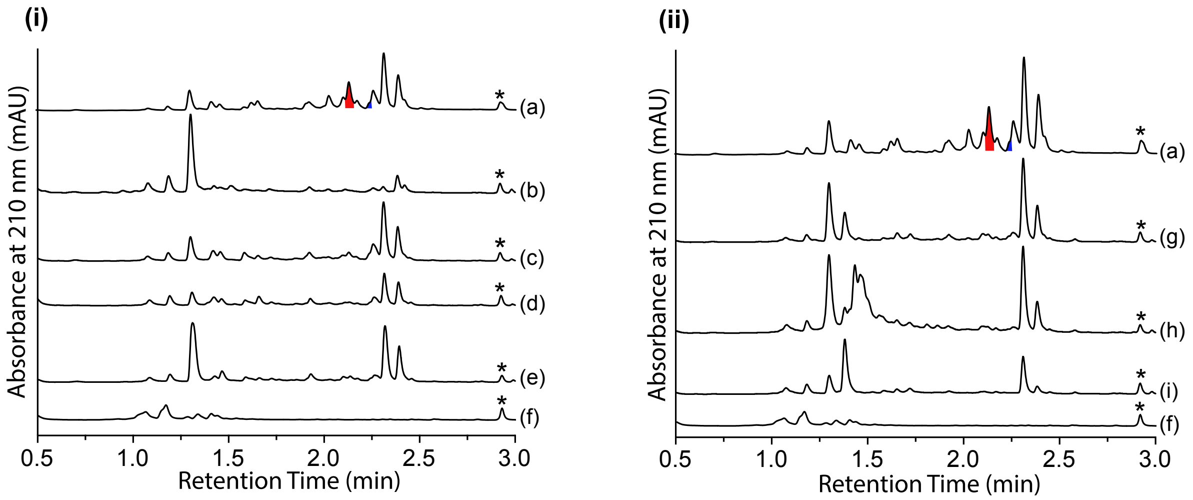

Figure S13. UPLC-DAD (210 nm) of CMB-F455 cultivated in M1 media in the presence and absence of sodium salts of different acids at 2mg/mL **(i)** benzoic acid derivatives (a) CMB-F455 control; (b) picolinic acid; (c) isonicotinic acid; (d) nicotinic acid; (e) benzoic acid; (f) M1 media blank; **(ii)** cinnamic acid derivatives (a) CMB-F455 control; (g) coumaric acid; (h) caffeic acid; (i) cinnamic acid; (f) M1 media blank; *internal calibrant

11 Chrysosporazine T (1)

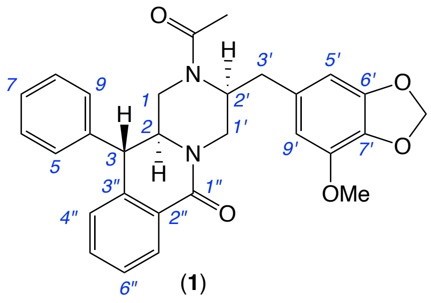

(1)

Table S3. 1D and 2D NMR (600 MHz, DMSO-d_6) data for chrysosporazine T (**1**) (major rotamer)

Position	δ_H, multi (*J* in Hz)	δ_C	COSY	^{1}H-^{13}C HMBC	ROSEY
1	*a.* 4.14, dd (*13.8, 3.9*)	40.1	1b, 2	2', 2, 1-NCO	1b, 2
	b. 2.94, m		1a, 2	2', 2, 3, 1-NCO	1a, 2, 3
2	3.84, ddd (*10.6, 10.6, 3.9*)	57.8	1a, 1b, 3	1, 3, 4, 3"	1a, 1'b, 5/9
3	4.47, d (*10.6*)	46.3	2	1, 2, 4, 5/9, 2", 3", 4"	1b, 5/9, 4"
4	-	140.4^A	-	-	-
5/9	7.35, m	129.3	6/8	3, 7, 5/9	2, 3
6/8	7.44, m	129.1	5/9	4, 6/8	-
7	7.37, m	127.6	6/8	5/9	-
1'	*a.* 4.57, dd (*13.3, 1.2*)	44.6	1'b, 2'	2, 1", 2', 3'	1'b, 2', 3'b
	b. 2.95, m		1'a, 2'	3'	1'a, 2', 2
2'	4.21, m	54.6	1'b, 3'ab	1, 3', 1-NCO	1'a, 1'b, 5', 9', 1-NCOCH_3
3'	*a.* 2.90, dd (*13.5, 8.1*)	34.9	2'	1', 2', 4', 5', 9'	3'b, 5', 9'
	b. 2.86, dd (*13.5, 6.6*)		2'	1', 2', 4', 5', 9'	3'a, 1'a, 5', 9'
4'	-	132.5	-	-	-
5'	6.54, d (*1.2*)	103.3	9'	3', 9', 7', 6'	1'ab, 2', 3'ab, 1-NCOCH_3
6'	-	148.3	-	-	-
7'	-	133.0	-	-	-
8'	-	143.0	-	-	-
9'	6.55, d (*1.2*)	109.0	5'	3', 5', 7', 8'	1', 2', 3'ab, 8'-OCH_3, 1-NCOCH_3
1"	-	163.9	-	-	-
2"	-	127.4	-	-	-
3"	-	140.3^A	-	-	-
4"	6.60, d (*7.8*)	126.9^B	5"	3, 2", 6"	3
5"	7.44, m	132.3	4",6"	3", 7"	-
6"	7.39, m	127.0^B	5",7"	2", 4", 7"	-
7"	8.04, dd (*7.7, 1.4*)	127.6	6"	1", 3", 5", 6"	-
1-NCO	-	168.4	-	-	-
1-NCOCH_3	1.70, s	20.8	-	2', 1-NCO	2', 5', 9'
6'-OCH_2	5.94/5.93, AB_q	101.0	-	6', 7'	-
8'-OCH_3	3.79, s	56.2	-	8'	9'

A, B assignments with the same superscript within a column are interchangeable

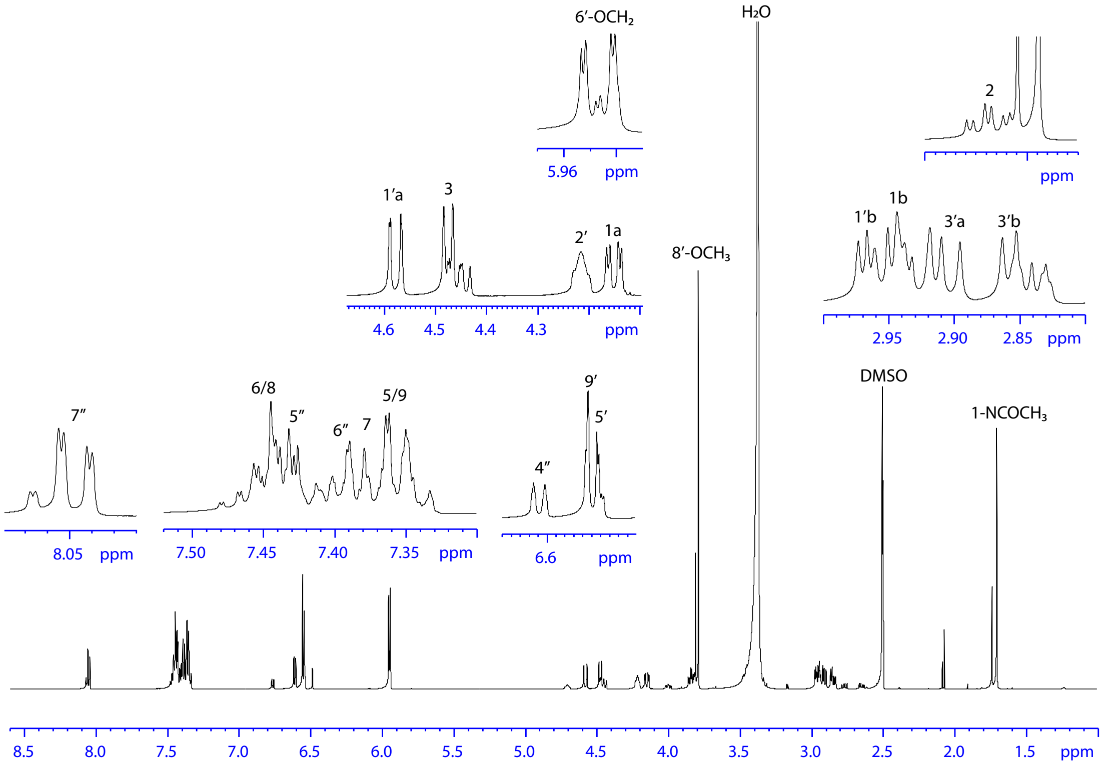

Figure S14. ^{1}H NMR (600 MHz, DMSO-d_6) spectrum for chrysosporazine T (**1**)

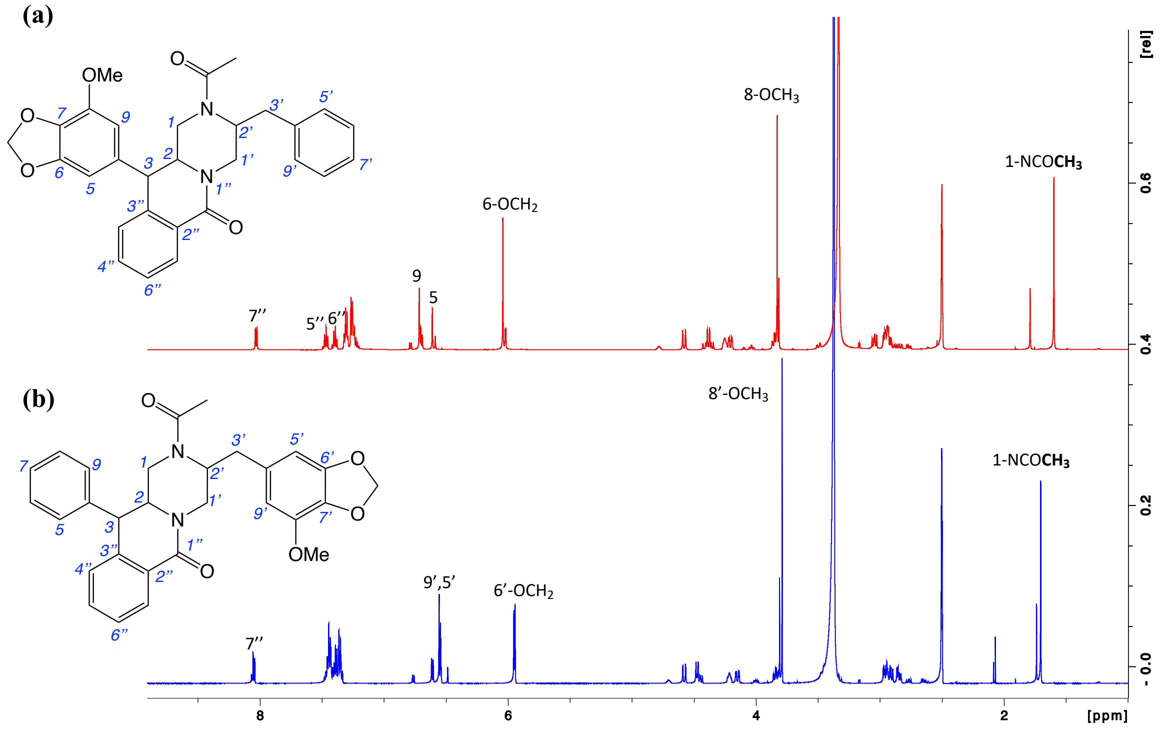

Figure S15. Comparison of ^{1}H NMR (600 MHz, DMSO-d_6) spectra of (a) chrysosporazine C (**7**) and (b) chrysosporazine T (**1**)

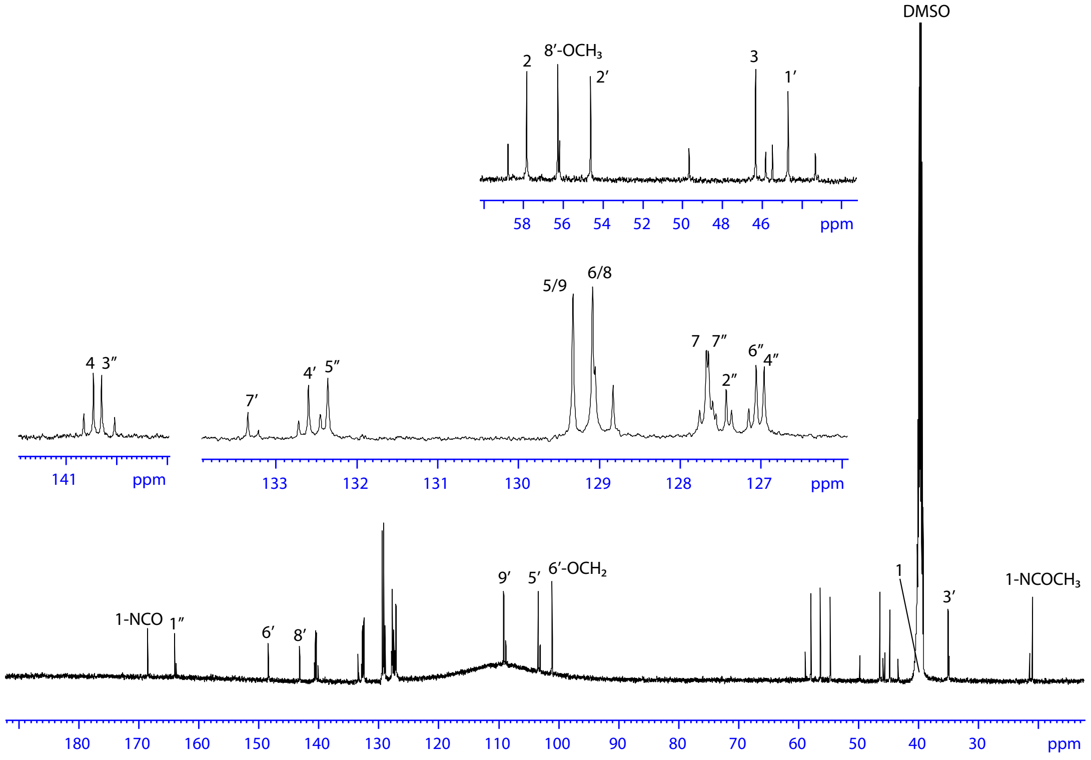

Figure S16. ^{13}C NMR (150 MHz, DMSO-d_6) spectrum for chrysosporazine T (**1**)

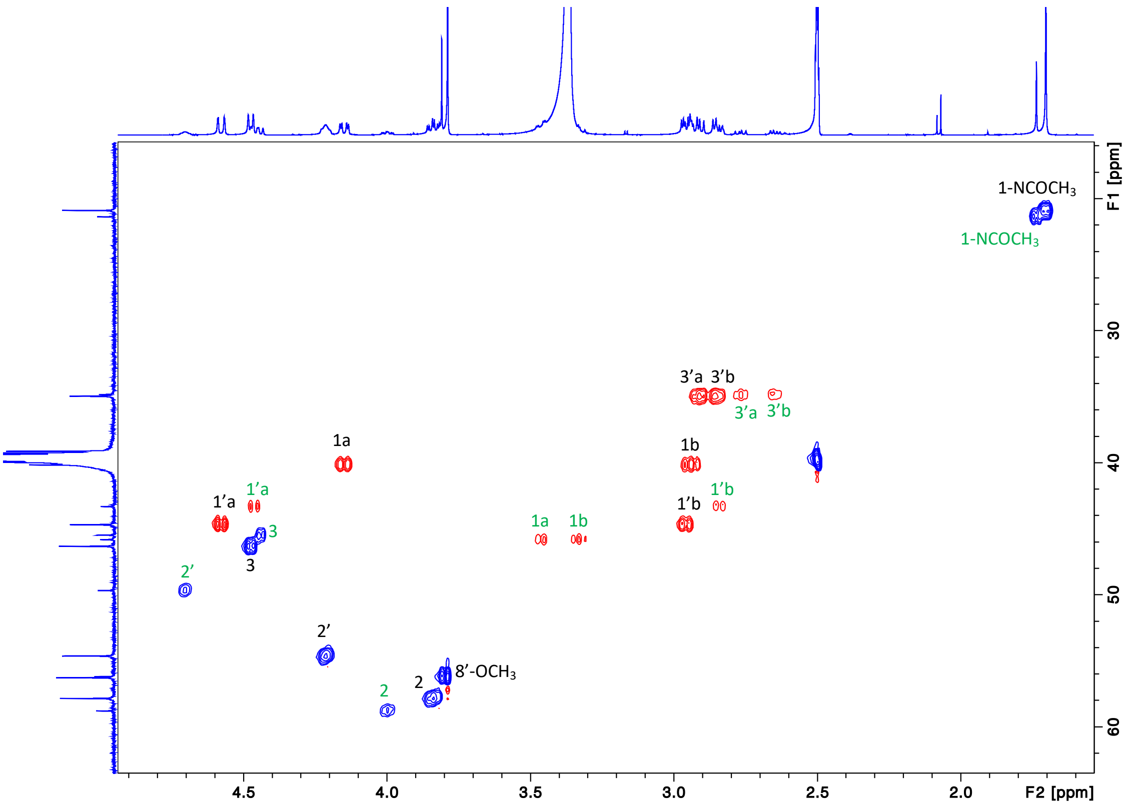

Figure S17. Expanded HSQC NMR (600 MHz, DMSO-d_6) spectrum (part 1) for chrysosporazine T (**1**), major rotamer (labelled black); minor rotamer (labelled green)

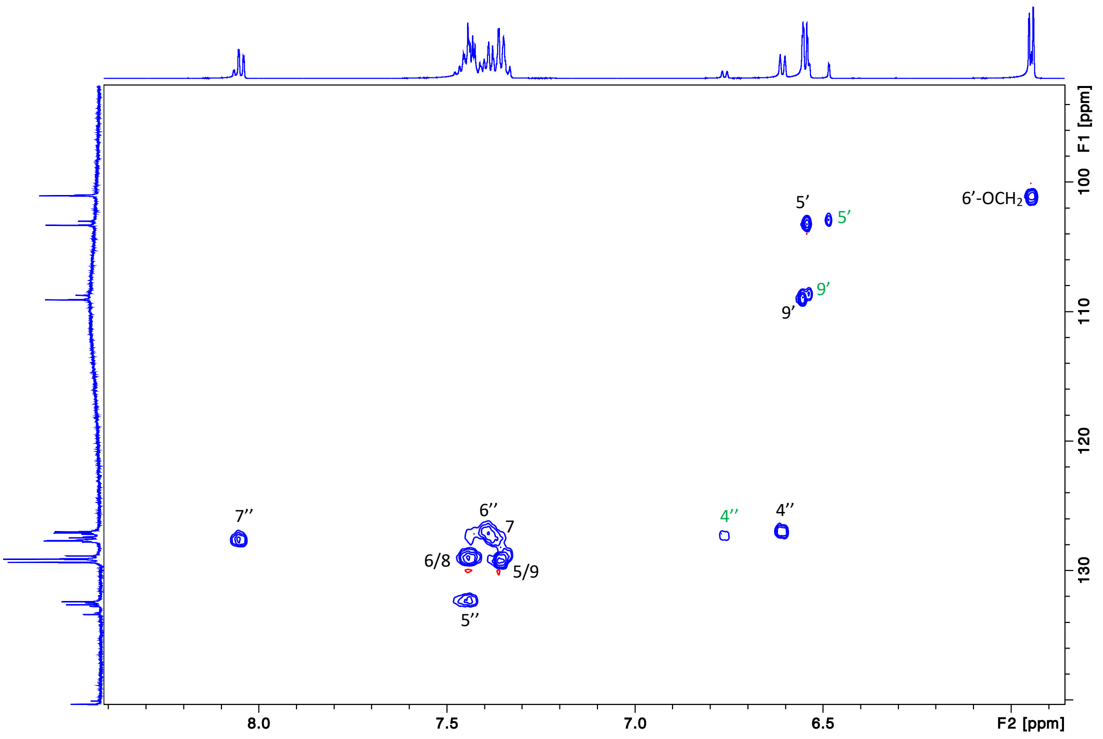

Figure S18. Expanded HSQC NMR (600 MHz, DMSO-d_6) spectrum (part 2) for chrysosporazine T (**1**), major rotamer (labelled black); minor rotamer (labelled green)

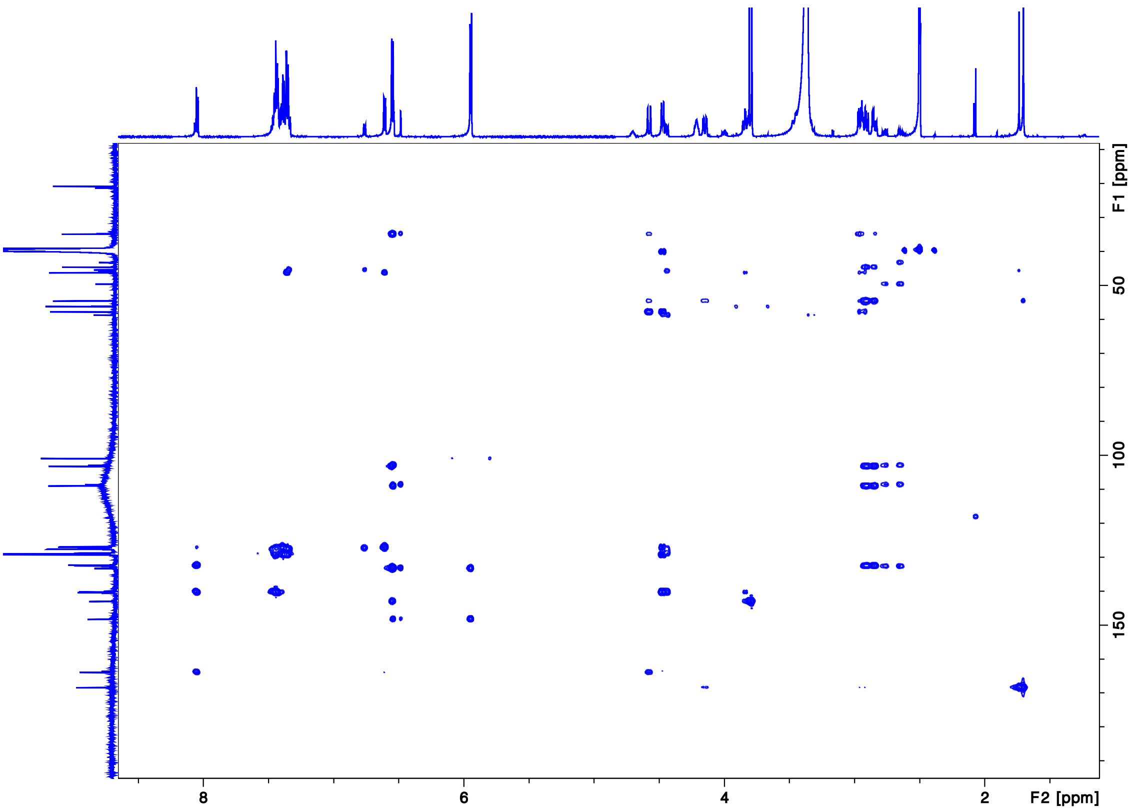

FigureS19. HMBC NMR (600 MHz, DMSO-d_6) spectrum for chrysosporazine T (**1**)

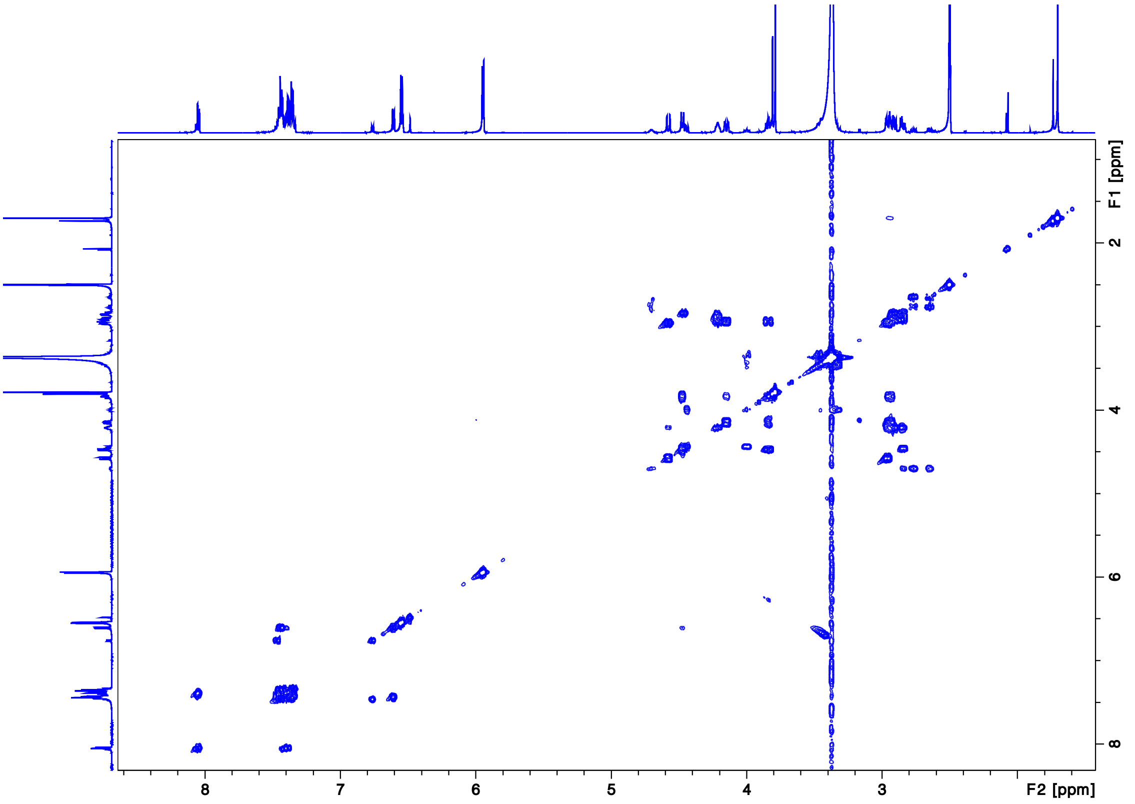

Figure S20. COSY NMR (600 MHz, DMSO-d_6) spectrum for chrysosporazine T (**1**)

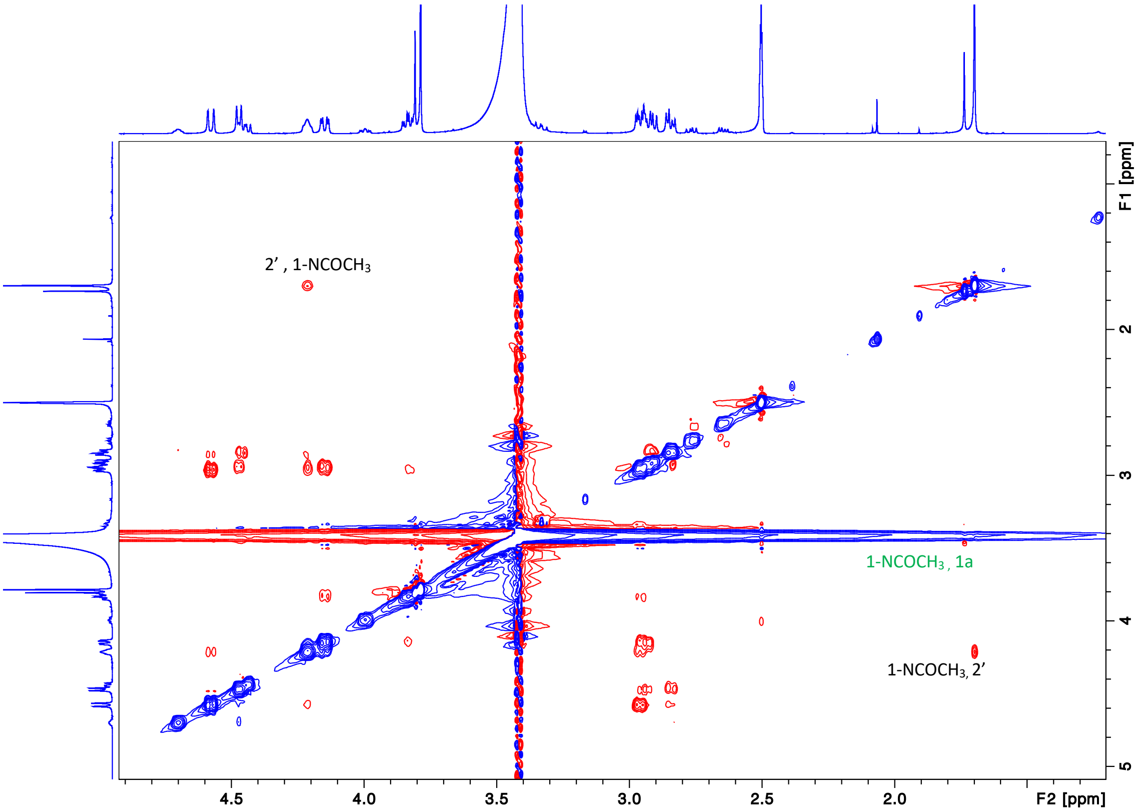

Figure S21. ROESY NMR (600 MHz, DMSO-d_6) spectrum for chrysosporazine T (**1**), major rotamer (labelled black); minor rotamer (labelled green)

12 Chrysosporazine U (2)

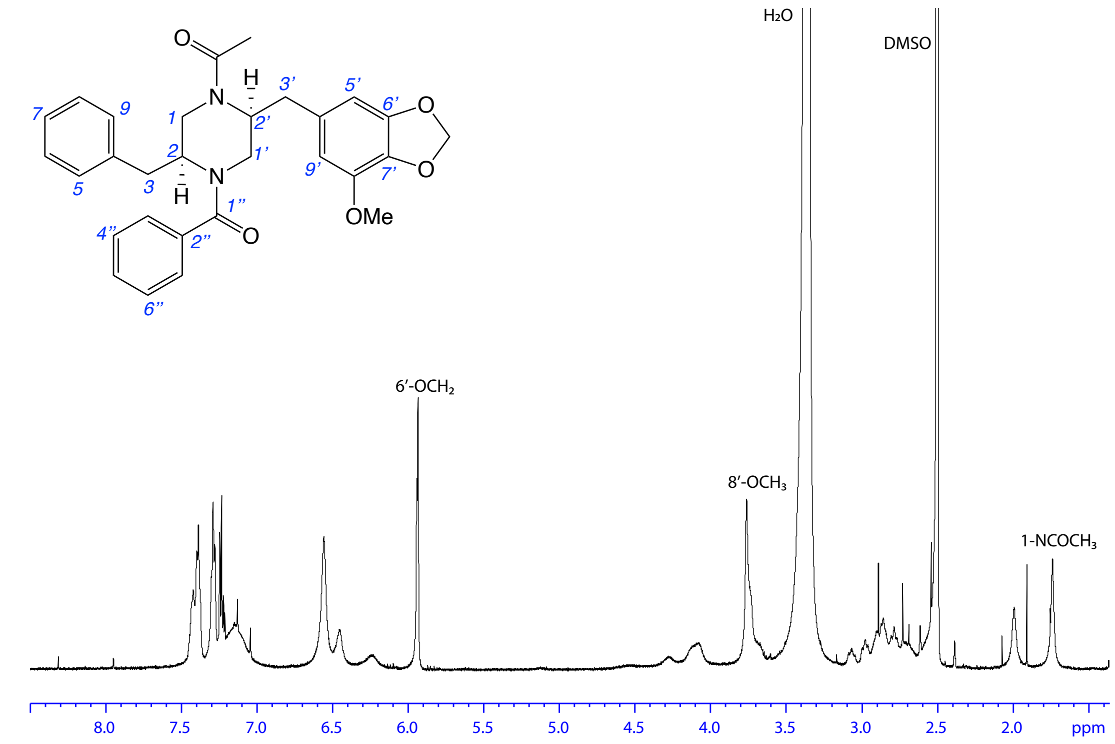

Figure S22. ^{1}H NMR (DMSO-*d*$_6$) spectrum for chrysosporazine U (**2**)

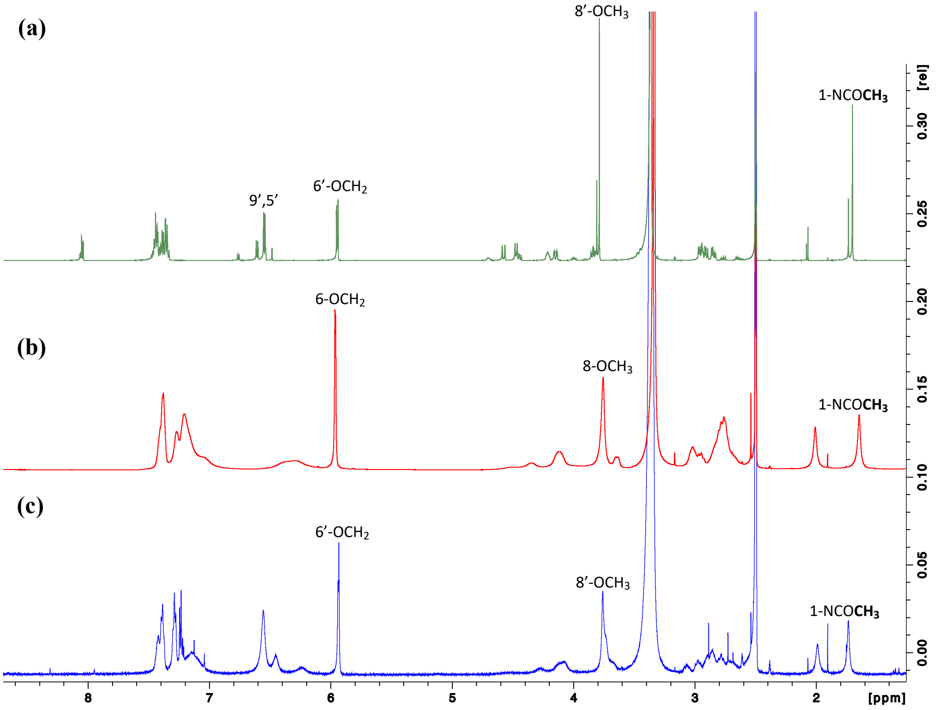

Figure S23. ^{1}H NMR (600 MHz, DMSO-*d*$_6$) spectrum for a) chrysosporazine T (**1**), b) chrysospirazine D (**7**) and chrysosporazine U (**2**)

13 Azachrysosporazine T1 (3)

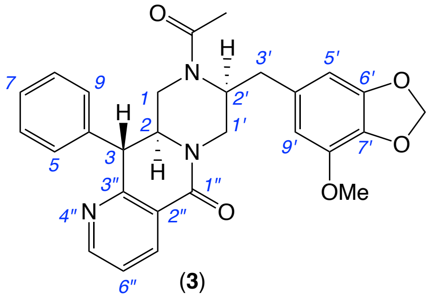

(**3**)

Table S4. 1D and 2D NMR (600 MHz, DMSO-d_6) data for azachrysosporazine T1 (**3**) (major rotamer)

Position	δ_H, multi (*J* in Hz)	δc	COSY	^{1}H-^{13}C HMBC	ROSEY
1	*a.* 4.25, dd (*13.3, 3.8*)	40.4	1b, 2	-	1b, 2
	b. 3.03, m		1a, 2	-	1a
2	3.87, ddd (*11.2, 8.8, 3.8*)	58.6	1a, 1b, 3	-	1a, 2', 5/9
3	4.56, d (*8.8*)	48.8	2	1, 2, 4, 5/9, 2", 3"	1b, 5/9
4	-	140.7	-	-	-
5/9	7.26, m	129.3	6/8	3, 7, 5/9	2, 3, 1b
6/8	7.35, m	128.6	5/9	4, 6/8	-
7	7.29, m	127.1	6/8	5/9	-
1'	*a.* 4.56, m	45.0	1'b, 2'	2	1'b, 2', 3'ab, 5', 9'
	b. 3.01, m		1'a, 2'	2	1'a, 2', 5', 9'
2'	4.21, m	54.9	1'b, 3'ab	-	1'a, 1'b, 5', 9', 2, 1-NCOCH3
3'	*a.* 2.88, dd (*13.5*, *8.3*)	35.0	3'b, 2'	1', 2', 4', 5', 9'	1'a, 5', 9'
	b. 2.83, dd (*13.5*, *6.3*)		3'a, 2'	2', 4', 5', 9'	1'a, 5', 9'
4'	-	132.6	-	-	-
5'	6.54, d (*1.3*)	103.4	9'	3', 9', 7', 6'	1', 2', 3'ab, 1-NCOCH3
6'	-	148.2	-	-	-
7'	-	133.4	-	-	-
8'	-	143.1	-	-	-
9'	6.55, d (*1.3*)	109.1	5'	3', 5', 7', 8'	1', 2', 3'ab, 8'-OCH3, 1-NCOCH3
1"	-	163.2	-	-	-
2"	-	122.9[A]	-	-	-
3"	-	158.4	-	-	-
N					
5"	8.57, dd (*4.7, 1.8*)	152.6	6"	ND	-
6"	7.44, ddd (*7.8, 4.7*)	122.8[A]	5",7"	ND	-
7"	8.34, dd (*7.8, 1.8*)	135.6	6"	1", 3", 5"	-
1-NCO	-	168.6	-	-	-
1-NCOCH3	1.68, s	20.8	-	1-NCO	2', 5', 9'
6'-OCH2	5.95/5.93, AB_q	101.0	-	6', 7'	-
8'-OCH3	3.78, s	56.3	-	8'	9'

[A] assignments with the same superscript within a column are interchangeable

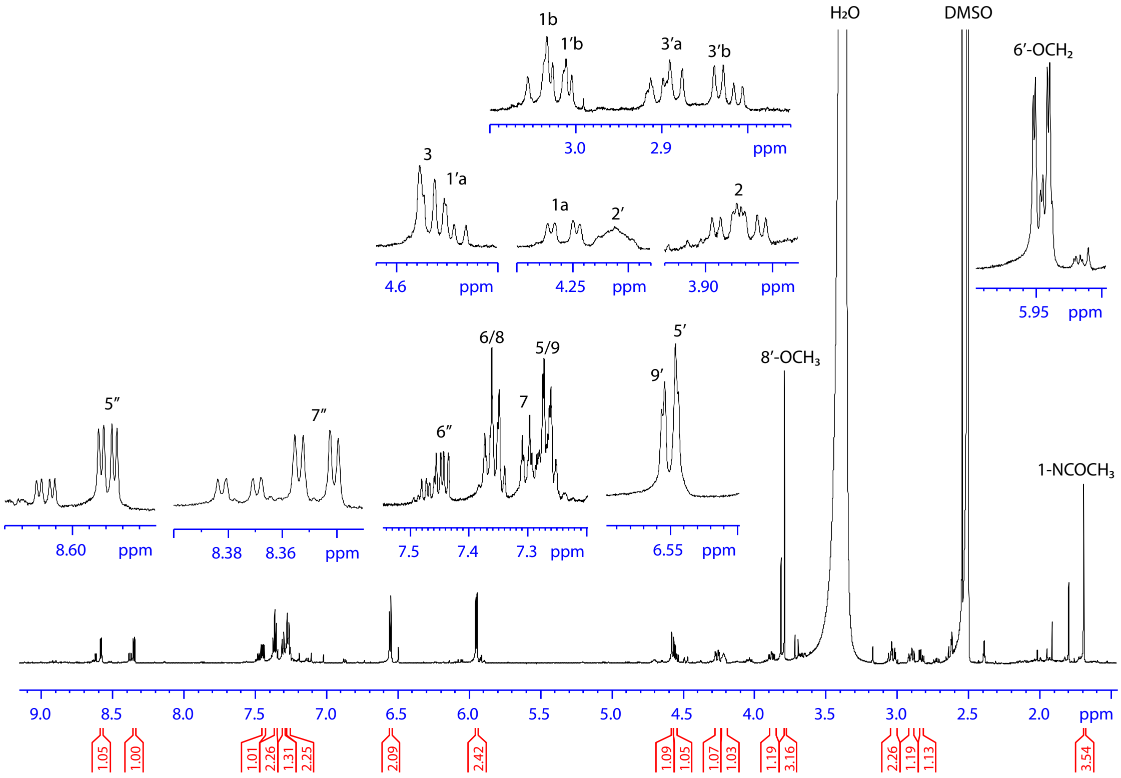

Figure S24. ^{1}H NMR (600 MHz, DMSO-d_6) spectrum for azachrysosporazine T1 (**3**)

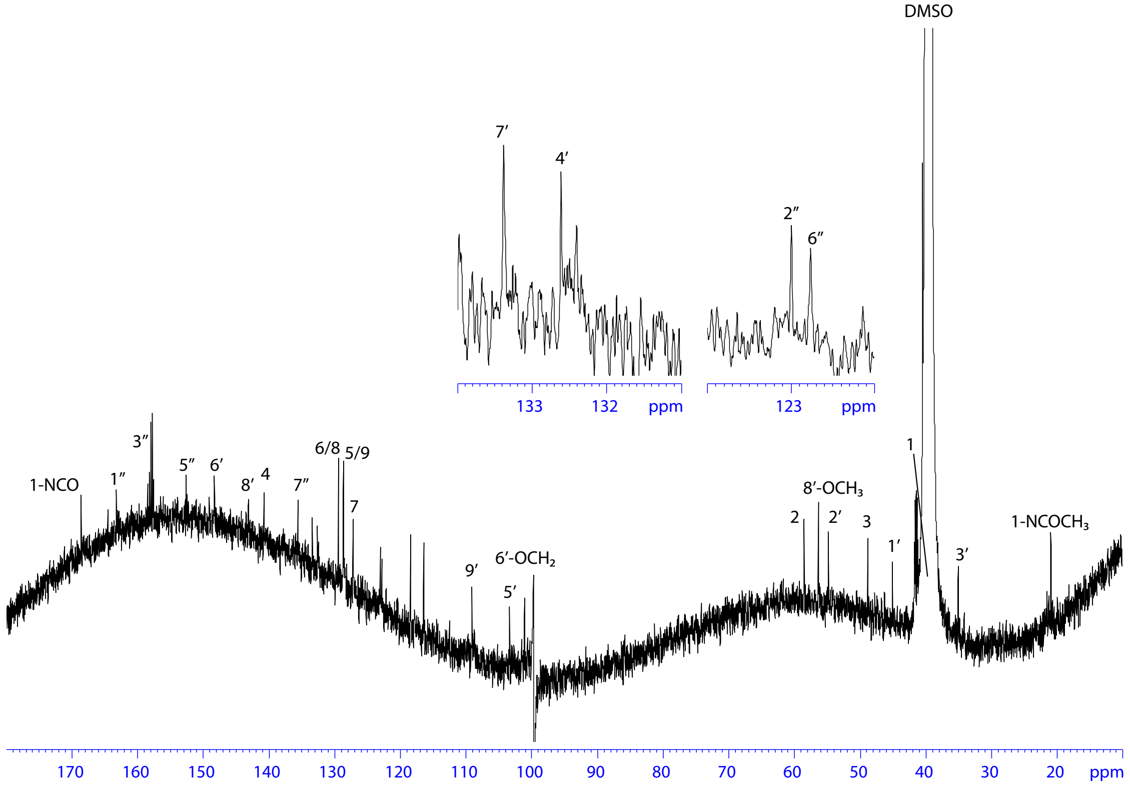

Figure S25. ^{13}C NMR (150 MHz, DMSO-d_6) spectrum for azachrysosporazine T1 (**3**)

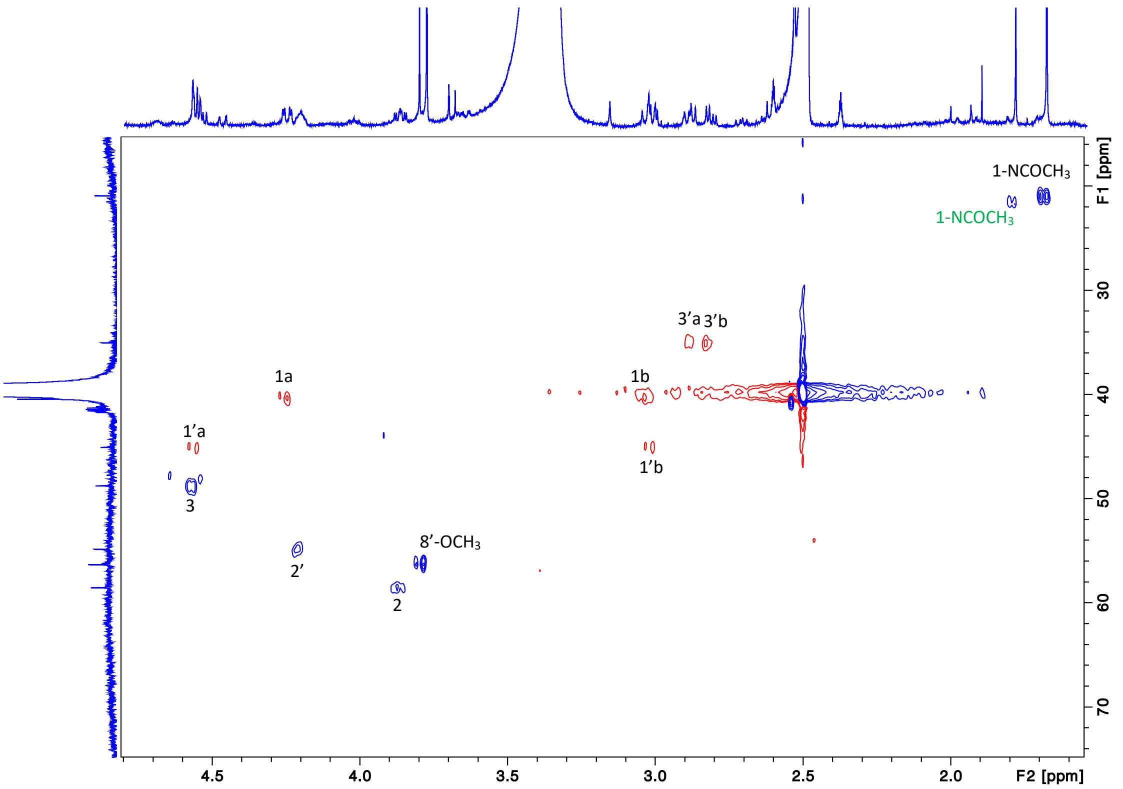

Figure S26. Expanded HSQC NMR (600 MHz, DMSO-d_6) spectrum (part 1) for azachrysosporazine T1 (**3**), major rotamer (labelled black); minor rotamer (labelled green)

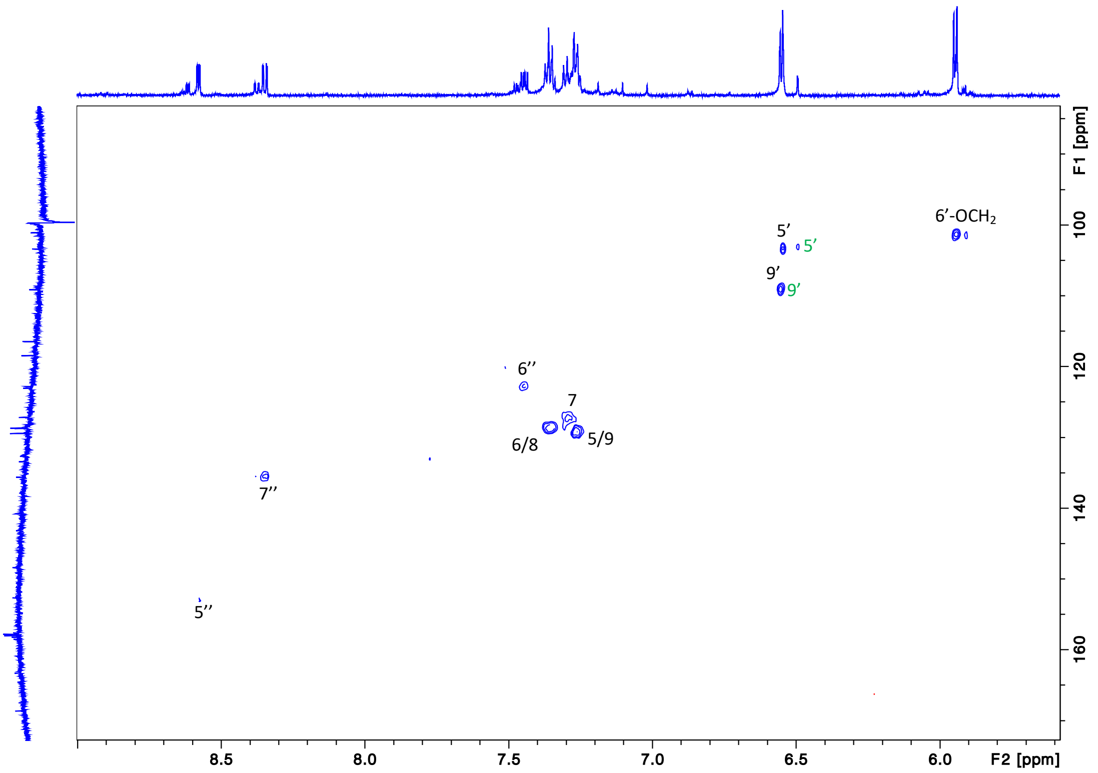

Figure S27. Expanded HSQC NMR (600 MHz, DMSO-d_6) spectrum (part 2) for azachrysosporazine T1 (**3**), major rotamer (labelled black); minor rotamer (labelled green)

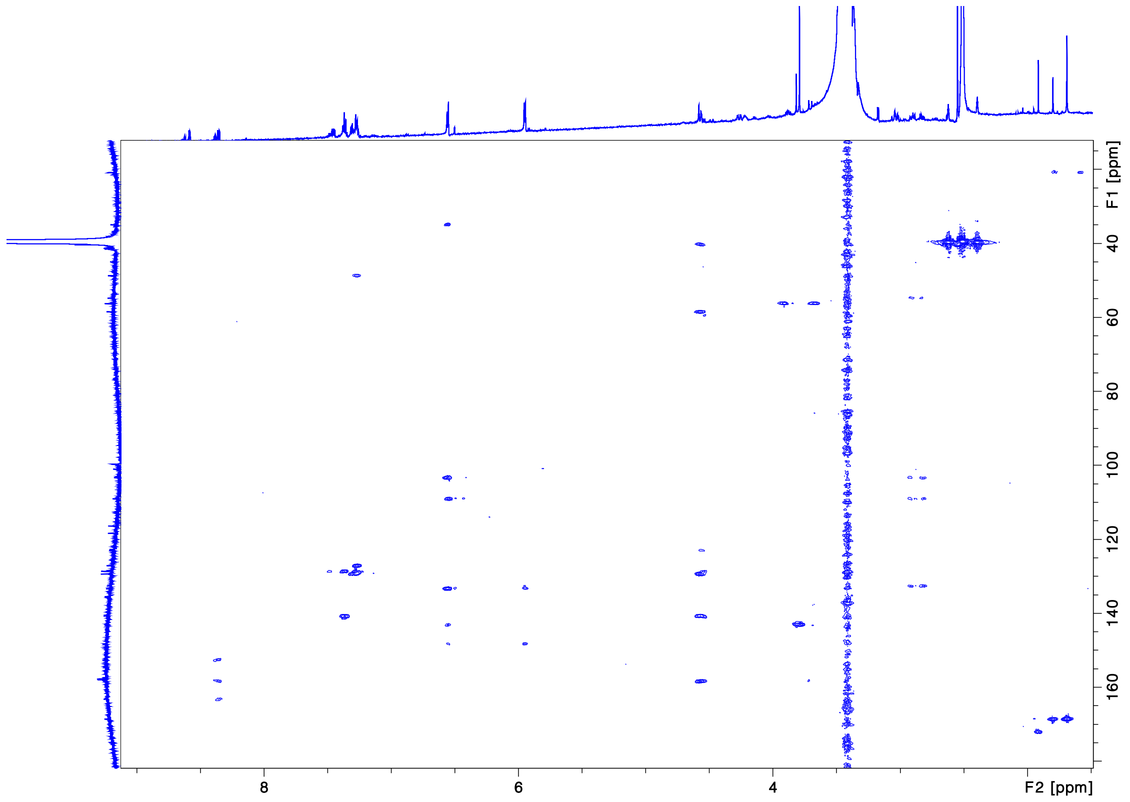

Figure S28. HMBC NMR (600 MHz, DMSO-d_6) spectrum for azachrysosporazine T1 (**3**)

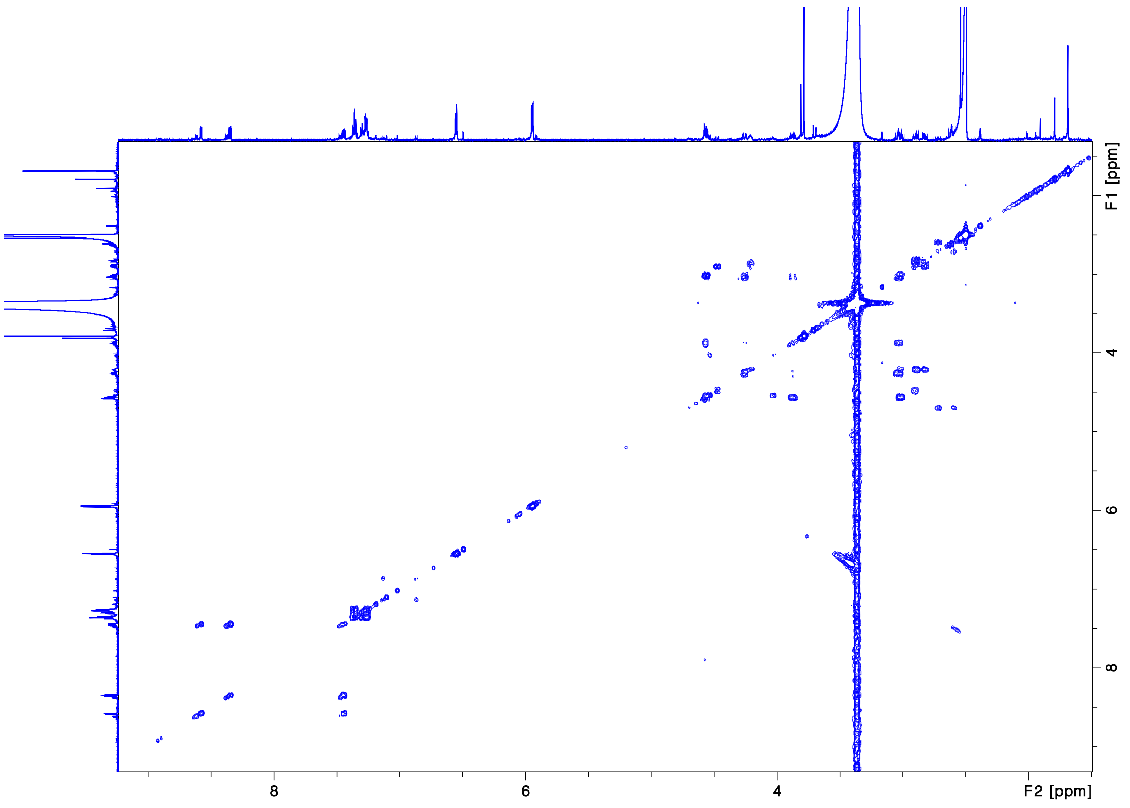

Figure S29. COSY NMR (600 MHz, DMSO-d_6) spectrum for azachrysosporazine T1 (**3**)

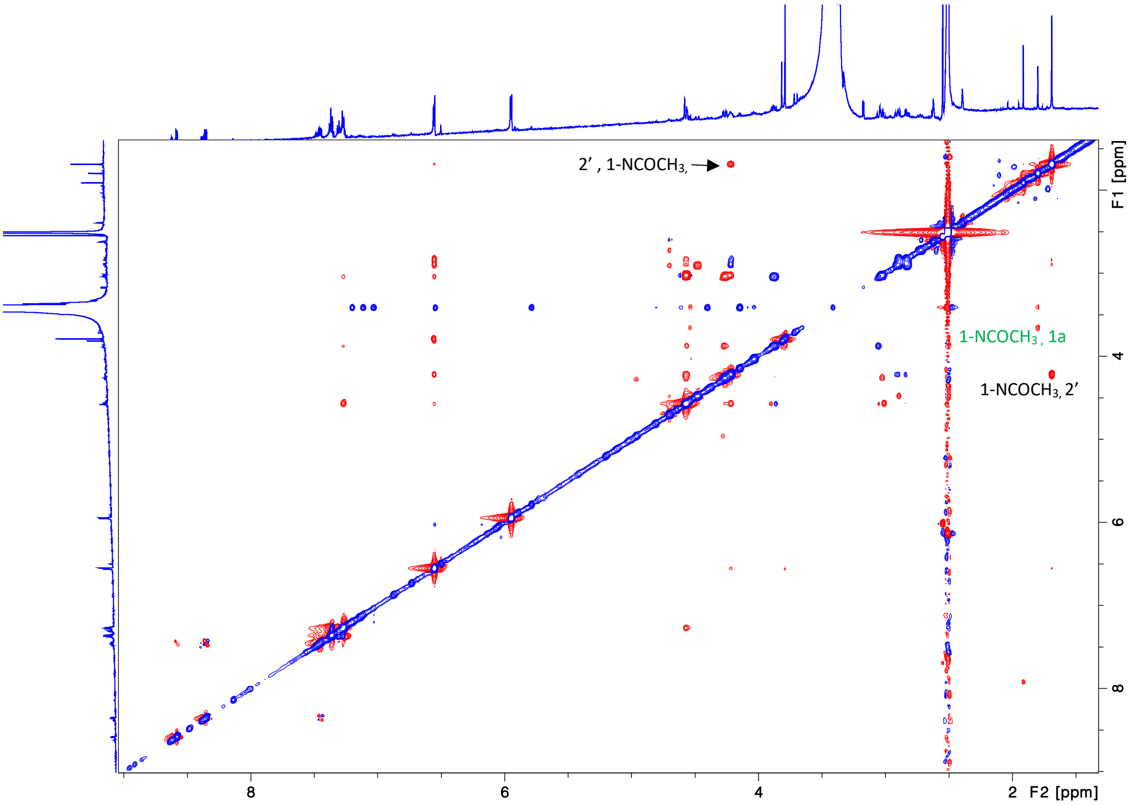

Figure S30. ROESY NMR (600 MHz, DMSO-d_6) spectrum for azachrysosporazine T1 (**3**), major rotamer (labelled black); minor rotamer (labelled green)

14 Azachrysosporazine U1 (4)

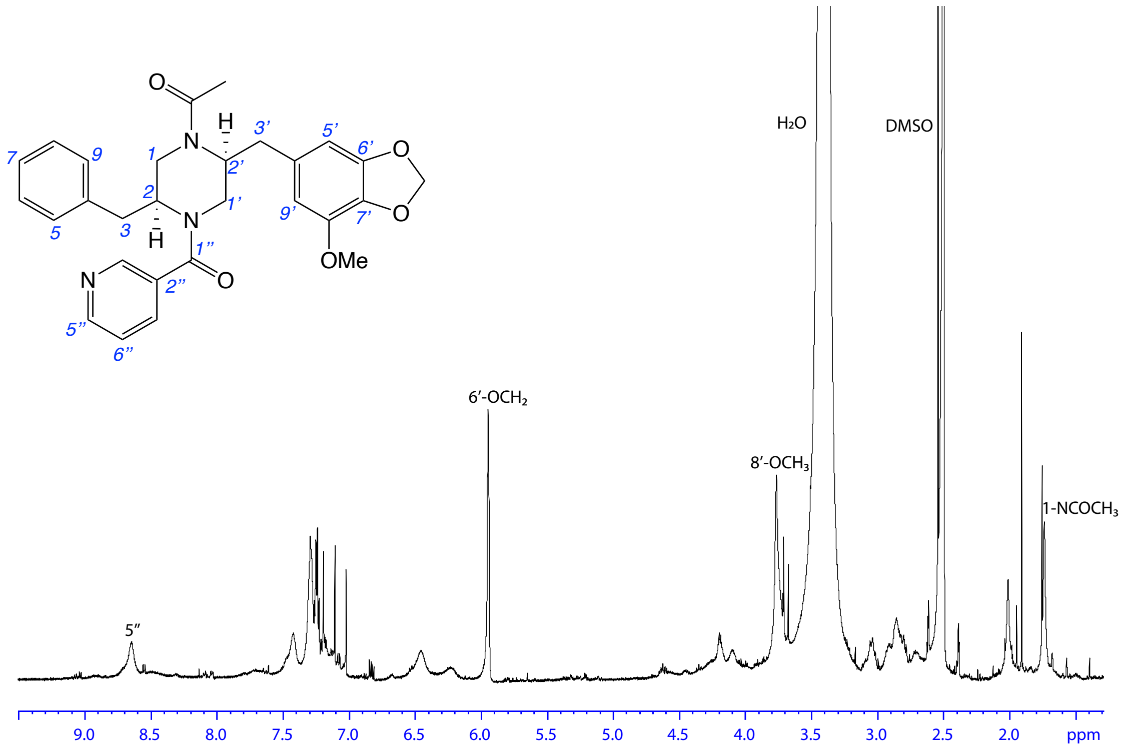

Figure S31. ^{1}H NMR (DMSO-d_6) spectrum for azachrysosporazine U1 (**4**)

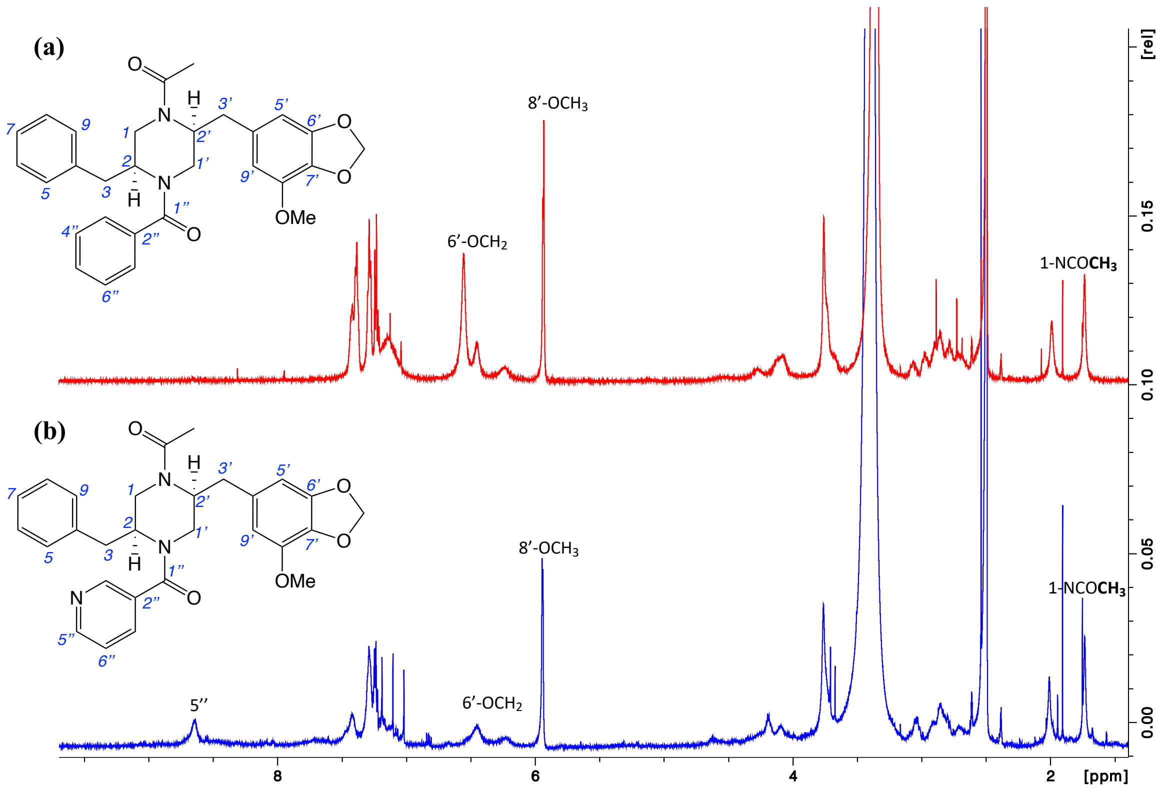

Figure S32. ^{1}H NMR (600 MHz, DMSO-d_6) spectrum for a) chrysosporazine U (**2**), b) azachrysospirazine U1 (**4**)

15 Neochrysosporazine R (5)

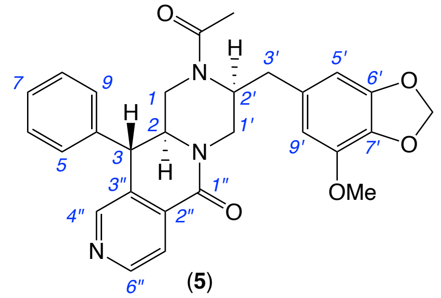

(5)

Table S5. 1D and 2D NMR (600 MHz, DMSO-d_6) data for neochrysosporazine R (**5**) (major rotamer)

Position	δ_H, multi (*J* in Hz)	δ_C	COSY	^{1}H-^{13}C HMBC	ROSEY
1	*a.* 4.19, dd (*14.5, 4.0*)	40.1	1b, 2	-	1b, 2, 5/9
	b. 2.98, m		1a, 2	-	1a, 2
2	3.92, ddd (*14.5, 10.3, 3.7*)	58.2	1a, 1b, 3	-	1a, 1b, 5/9
3	4.56, d (*10.3*)	43.7	2, 4"	1, 2, 4, 5/9, 2", 3", 4"	1b, 5/9
4	-	139.4	-	-	-
5/9	7.40, m	129.2^A	6/8	3, 7, 5/9	1b, 2, 3
6/8	7.46, m	129.1^A	5/9	4, 6/8	-
7	7.40, m	127.9	6/8	5/9	-
1'	*a.* 4.55, dd (*13.4, 1.3*)	44.8	1'b	2, 1"	1'b, 2', 3'a, 3'b, 5', 9'
	b. 2.99, m		1'a, 2'	-	1'a, 2', 5', 9'
2'	4.23, m	54.6	1b, 3'a, 3'b	-	1'a, 1'b, 5', 9', 1-NCOCH$_3$
3'	*a.* 2.90, dd (*13.4, 8.6*)	34.8	2'	1', 2', 4', 5', 9'	1'a, 5', 9'
	b. 2.85, dd (*13.4, 6.4*)		2'	2', 4', 5', 9'	1'a, 5', 9'
4'	-	132.6	-	-	-
5'	6.54, d (*1.4*)	103.3	9'	3', 9', 7', 6'	1', 2', 3', 1-NCOCH$_3$
6'	-	148.3	-	-	-
7'	-	133.3	-	-	-
8'	-	143.0	-	-	-
9'	6.55, d (*1.4*)	109.0	5'	3', 5', 7', 8'	1', 2', 3', 8'-OCH$_3$
1"	-	162.3	-	-	-
2"	-	134.4	-	-	-
3"	-	133.7	-	-	-
4"	7.89, s	148.6	3	3, 2"	-
N					
6"	8.64, d (*4.7*)	148.3	7"	ND	-
7"	7.88, d (*4.7*)	120.2	6"	1", 2", 6"	-
1-NCO	-	168.5	-	-	-
1-NCOCH$_3$	1.70, s	20.8	-	1-NCO	2', 5'
6'-OCH$_2$	5.95/5.94, AB$_q$	101.0	-	6', 7'	-
8'-OCH$_3$	3.78, s	56.2	-	8'	9'

^{A} assignments with the same superscript within a column are interchangeable

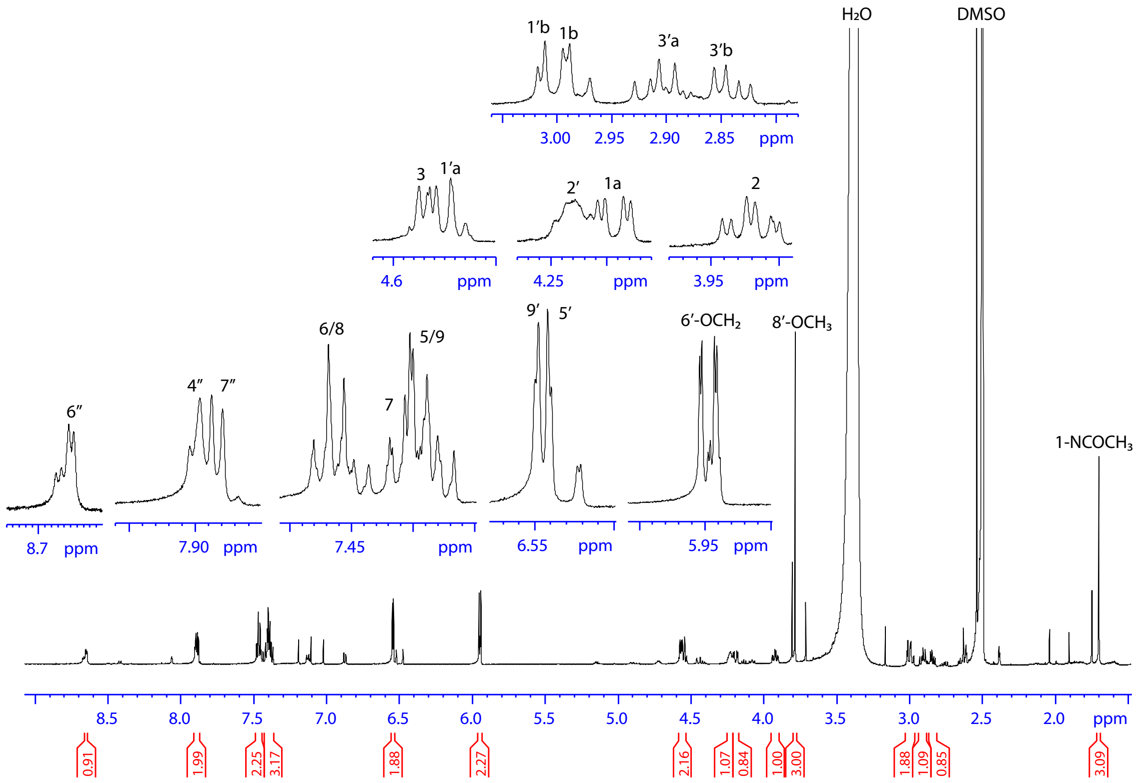

Figure S33. ^{1}H NMR (600 MHz, DMSO-d_6) spectrum for neochrysosporazine R (**5**)

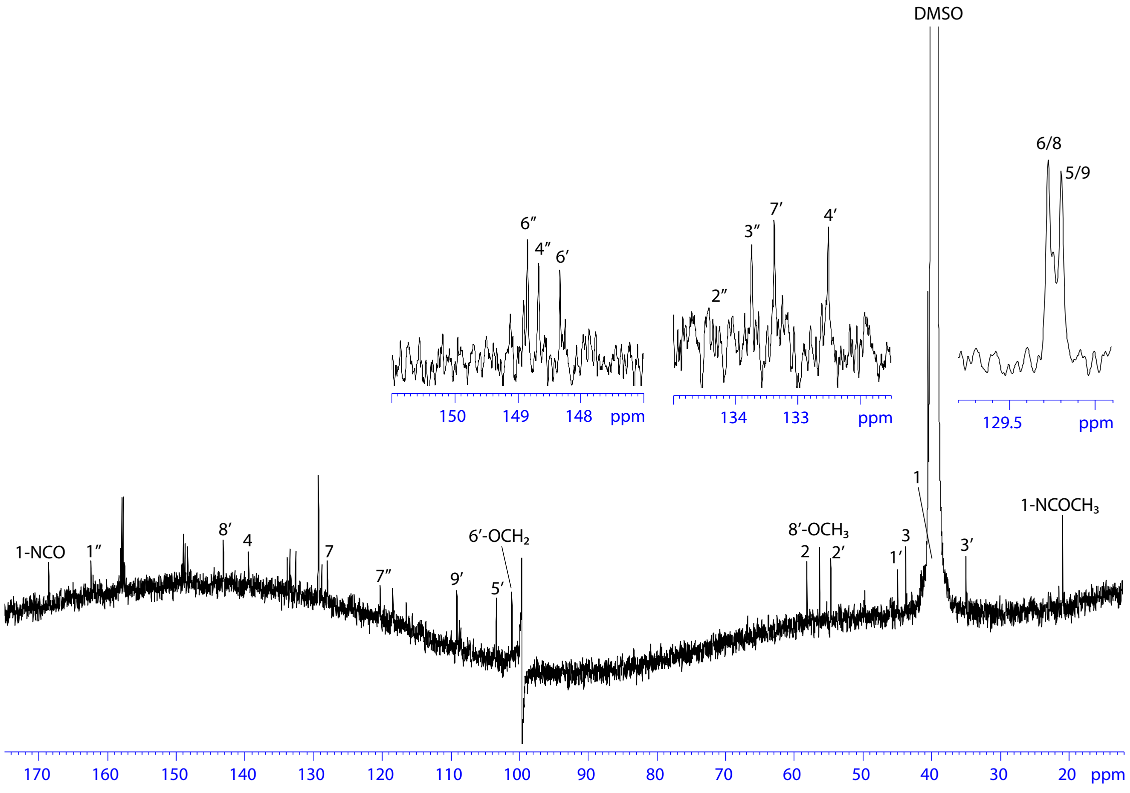

Figure S34. ^{13}C NMR (150 MHz, DMSO-d_6) spectrum for neochrysosporazine R (**5**)

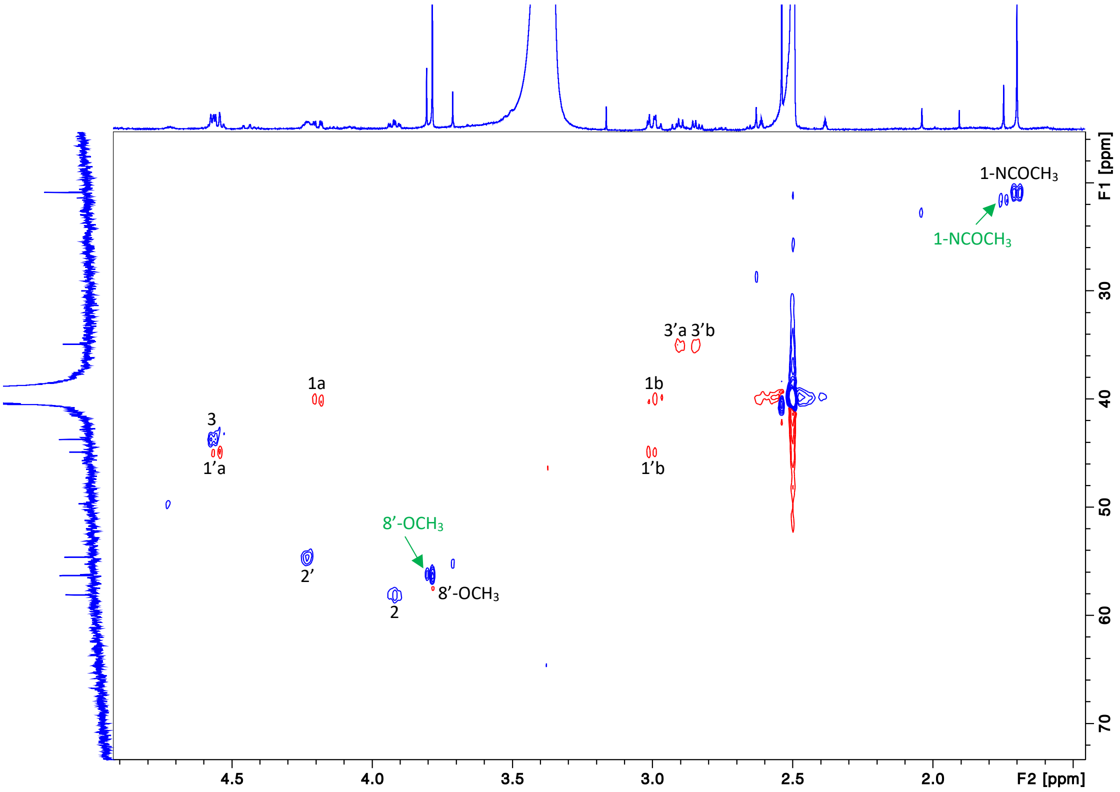

Figure S35. Expanded HSQC NMR (600 MHz, DMSO-d_6) spectrum (part 1) for neochrysosporazine R (**5**), major rotamer (labelled black); minor rotamer (labelled green)

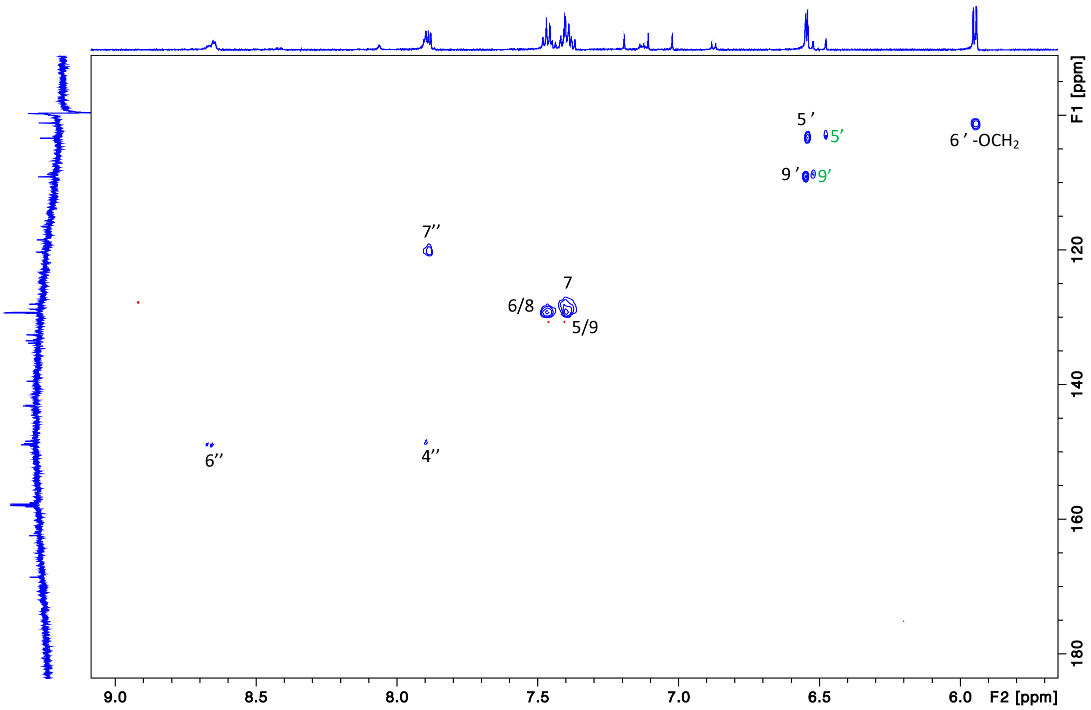

Figure S36. Expanded HSQC NMR (600 MHz, DMSO-d_6) spectrum (part 2) for neochrysosporazine R (**5**), major rotamer (labelled black); minor rotamer (labelled green)

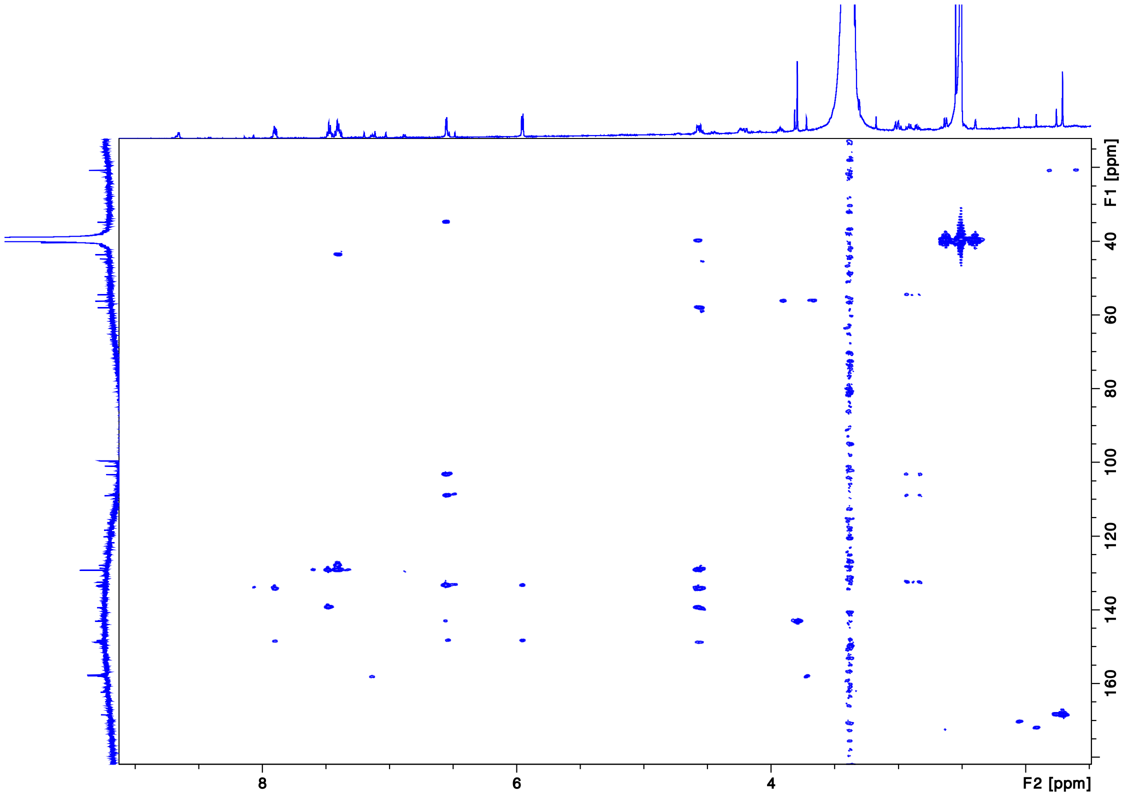

Figure S37. HMBC NMR (600 MHz, DMSO-d_6) spectrum for neochrysosporazine R (**5**)

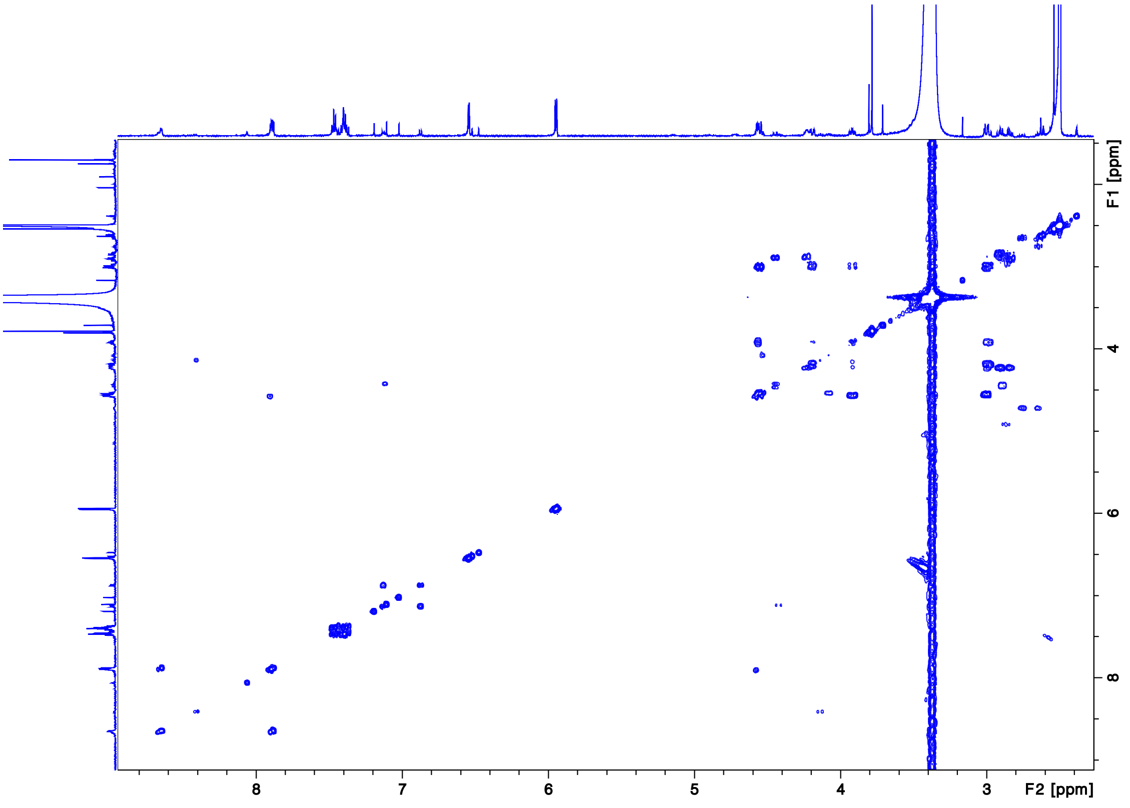

Figure S38. COSY NMR (600 MHz, DMSO-d_6) spectrum for neochrysosporazine R (**5**)

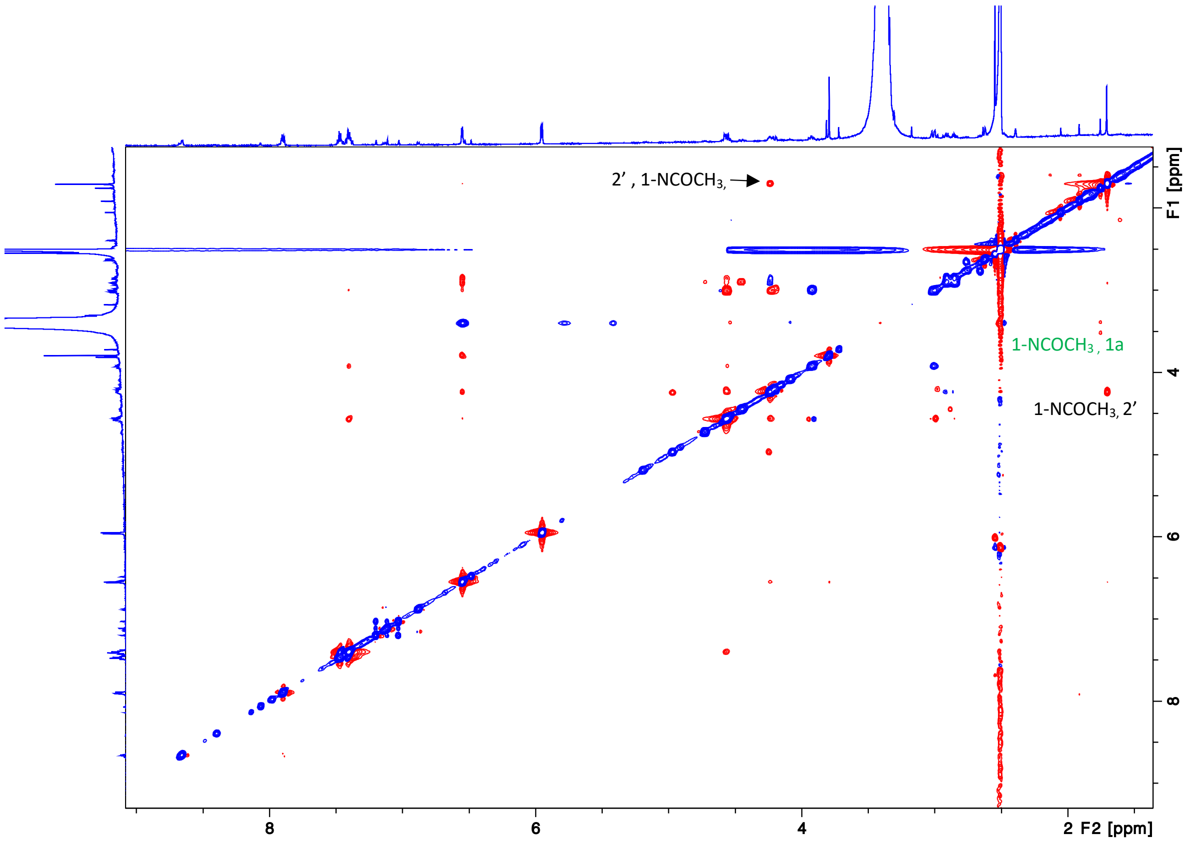

Figure S39. ROESY NMR (600 MHz, DMSO-d_6) spectrum for neochrysosporazine R (**5**), major rotamer (labelled black); minor rotamer (labelled green)

16 Neochrysosporazine S (6)

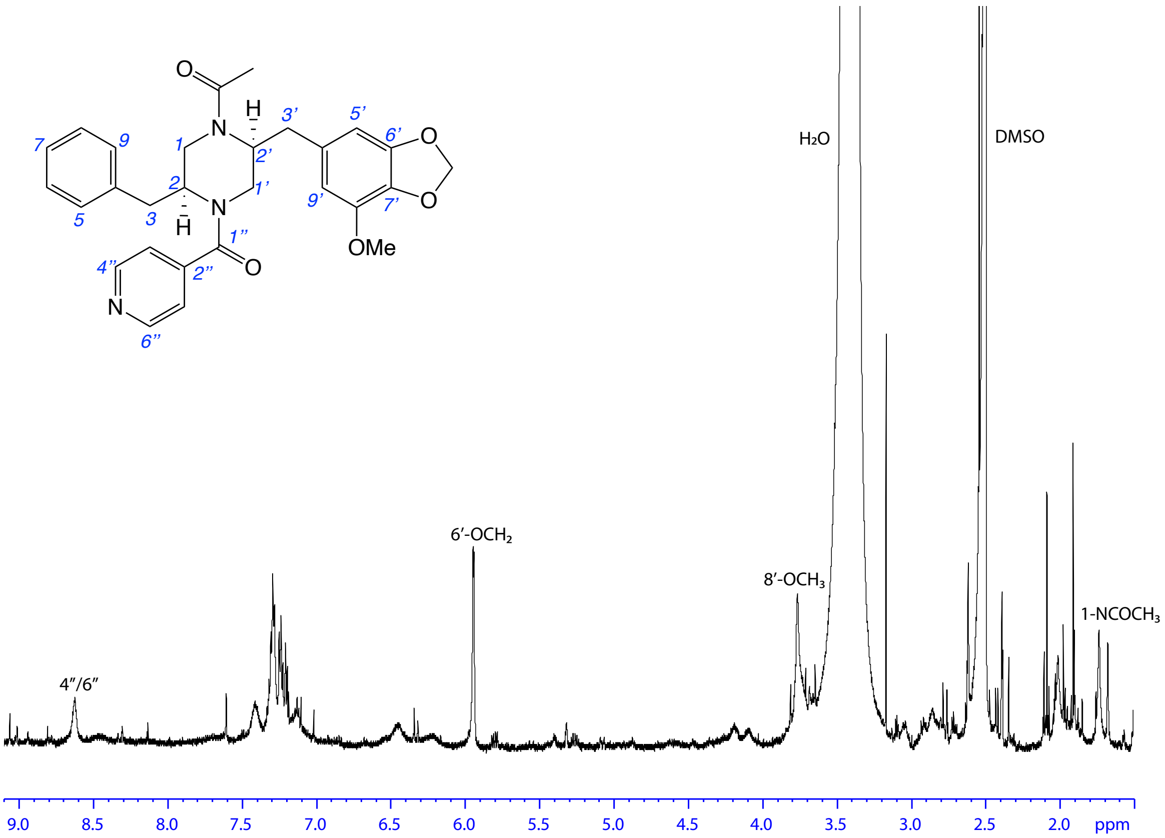

Figure S40. ^{1}H NMR (DMSO-*d*$_6$) spectrum for neochrysosporazine S (**6**)

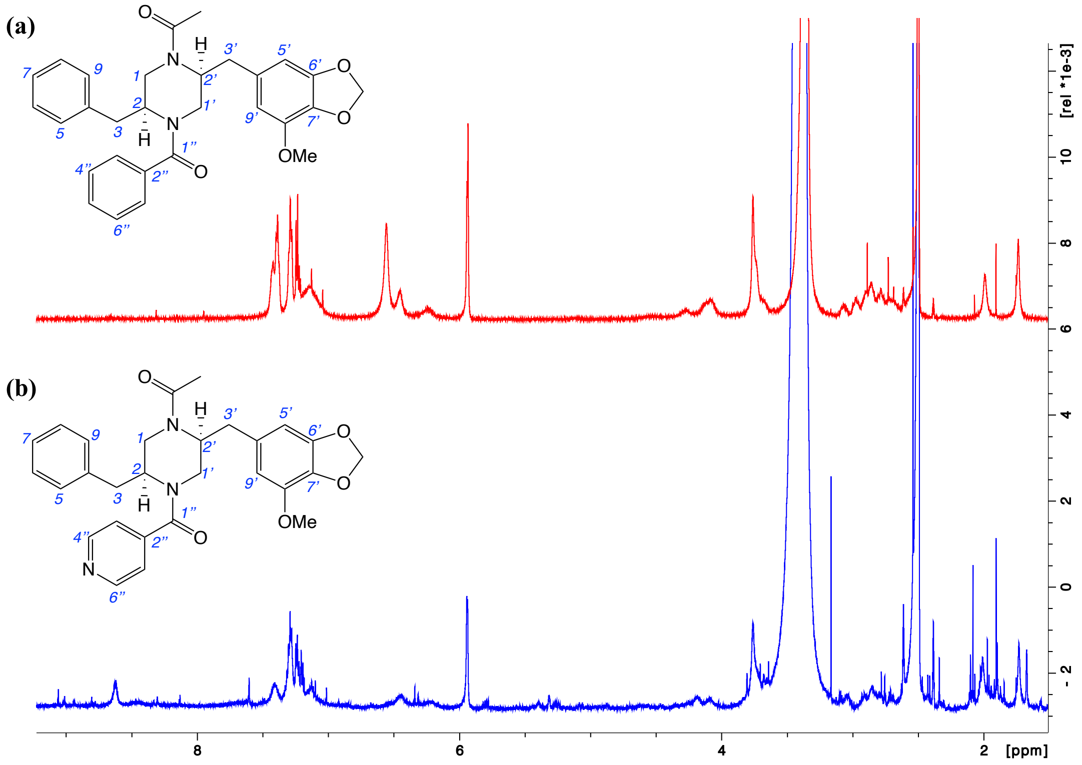

Figure S41. ^{1}H NMR (600 MHz, DMSO-*d*$_6$) spectrum for a) chrysosporazine U (**2**), b) neochrysosporazine S (**6**)

17 Chrysosporazine D (7)

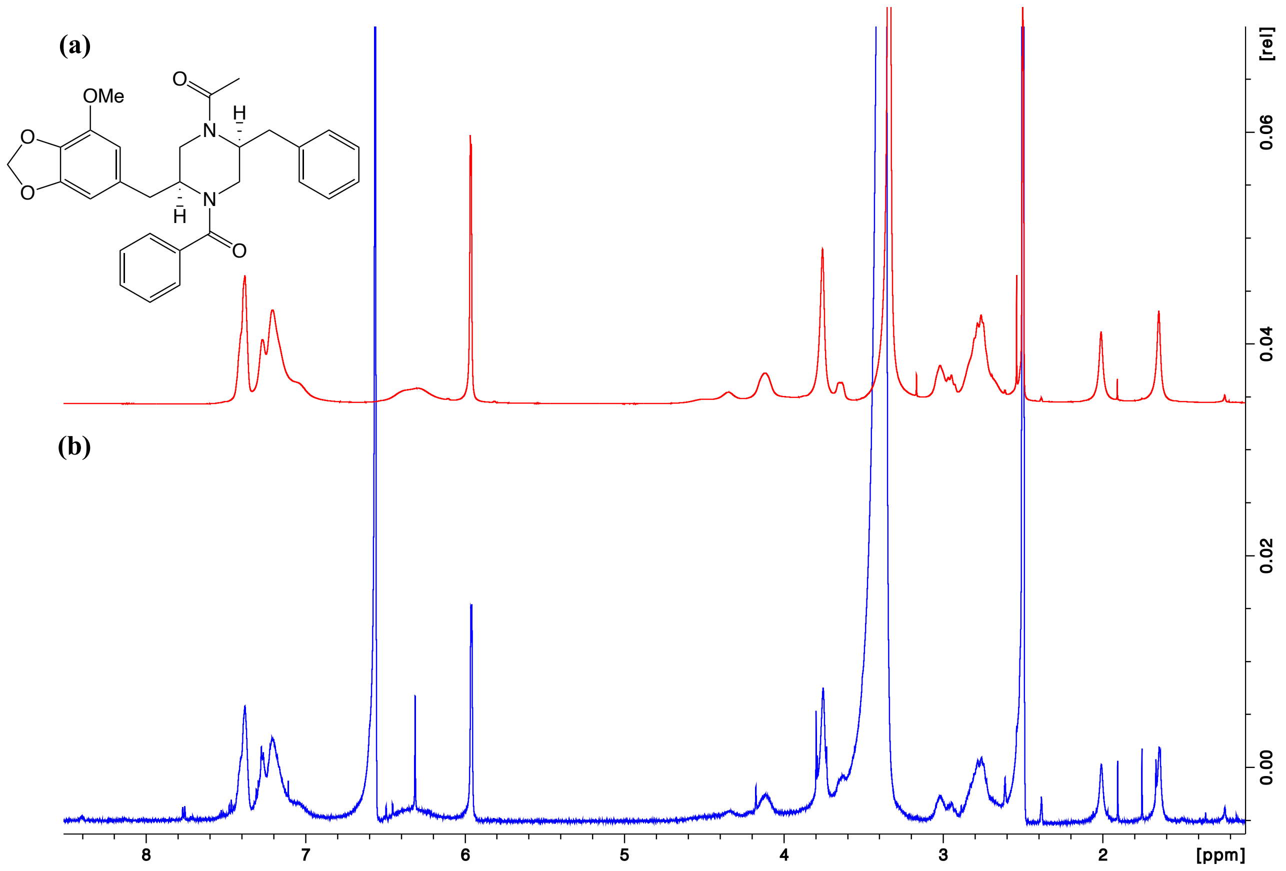

Figure S42. ^{1}H NMR (600 MHz, DMSO-d_6) spectrum for (a) known chrysosporazine D (**7**), (b) CMB-F455 isolated (**7**)

18 Brasiliamide A (8)

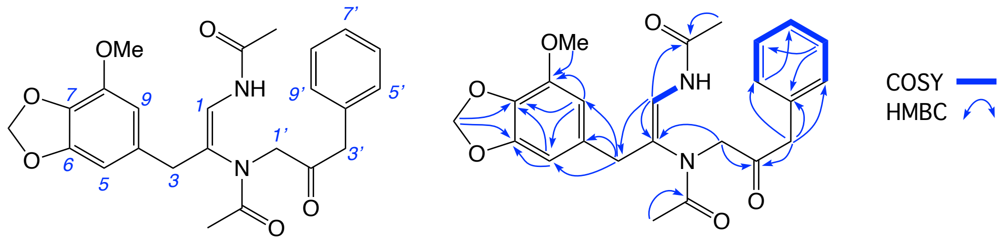

Table S6. 1D and 2D NMR (600 MHz, $CDCl_3$) data for brasiliamide A (**8**)

Position	δ_H, multi (*J* in Hz) for 8	δ_C	COSY	^1H-^{13}C HMBC	Lit δ_H[10]	Lit δ_C[10]
1	6.91, d (*10.3*)	122.2	1-NH	2, 3, 1-NHCO	6.92, d (*10.7*)	122.2
2	-	122.4	-	-	-	122.5
3	*a.* 3.29, d (*14.8*)<br>*b.* 3.18, d (*14.8*)	40.2	3b<br>3a	1, 4, 5, 9<br>1, 4, 5, 9	*a.* 3.29, d (*14.7*)<br>*b.* 3.19, d (*14.7*)	40.3
4	-	131.4	-	-	-	131.4
5	6.27, s	103.0	-	3, 9, 7, 6	6.28, d (*1.5*)	103.0
6	-	149.1	-	-	-	149.1
6-OCH_2	5.95/ 5.95, AB_q	101.5	-	6, 7	5.95, d (*1.5*)<br>5.95, d (*1.5*)	101.5
7	-	134.3	-	-	-	134.3
8	-	143.8	-	-	-	143.8
8-OCH_3	3.84, s	56.7	-	8	3.84, s	56.7
9	6.23, s	108.3	-	3, 5, 7, 8	6.23, d (*1.5*)	108.2
1-NH	9.62, d (*10.3*)	-	1	-	9.62, d (*10.7*)	
1-NHCO	-	168.3	-	-	-	168.3
1-NH$COCH_3$	2.11, s	22.3	-	1-NHCO	2.11, s	23.2
1'	*a.* 4.53, d (*17.1*)<br>*b.* 3.01, d (*17.1*)	56.6	1'b<br>1'a	2', 2<br>2', 1'-NCO, 2	*a.* 4.53, d (*17.4*)<br>*b.* 3.02, d (*17.4*)	56.6
2'	-	206.6	-	-	-	206.6
3'	*a.* 3.81, d (*16.0*)<br>*b.* 3.74, d (*16.0*)	47.9	3'b<br>3'a	2', 4', 5'/9'	*a.* 3.81, d (*16.2*)<br>*b.* 3.75, d (*16.2*)	47.9
4'	-	132.8	-	-	-	132.8
5'/9'	7.17, d (*7.4*)	129.4	6'/8'	3', 7', 5'/9'	7.17, m	129.5
6'/8'	7.33, dd (*7.4, 7.1*)	128.9	5'/9', 7'	4', 6'/8'	7.33, m	128.8
7'	7.29, d (*7.1*)	127.5	6'/8'	5'/9'	7.29, m	127.4
1'-NCO	-	170.8	-	-	-	170.8
1'-N$COCH_3$	1.87, s	20.1	-	1'-NCO	1.87, s	20.1

10. Fujita, T.; Makishima, D.; Akiyama, K.; Hayashi, H., New convulsive compounds, brasiliamides A and B, from Penicillium brasilianum batista JV-379. *Biosci Biotechnol Biochem* **2002,** *66*, 1697-705.

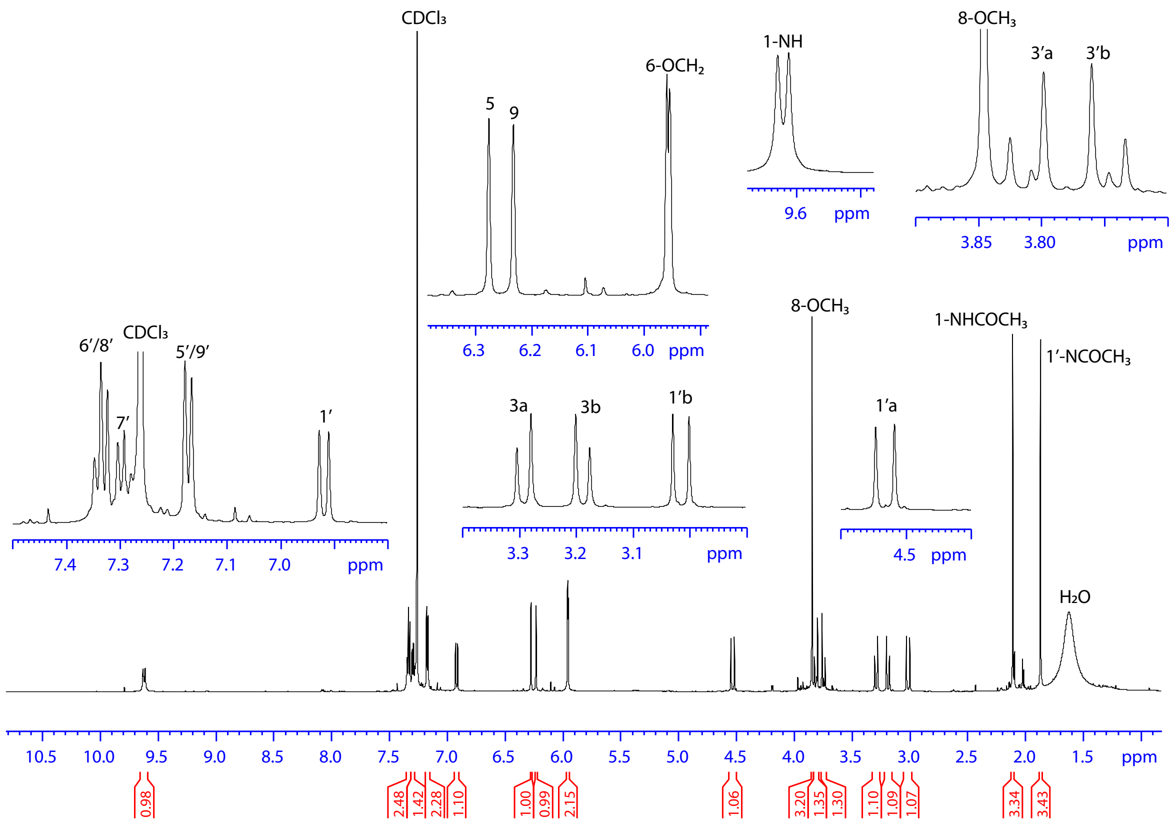

Figure S43. ^{1}H NMR ($CDCl_3$) spectrum for brasiliamide A (**8**)

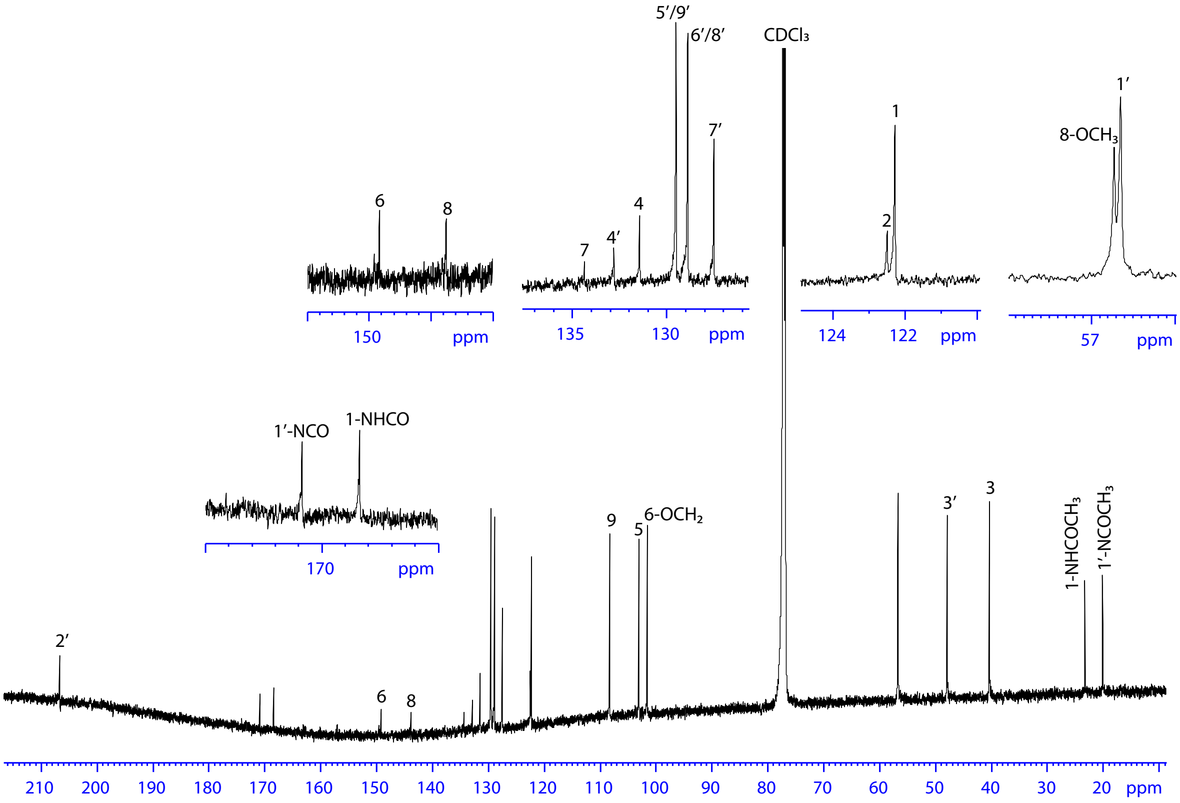

Figure S44. ^{13}C NMR ($CDCl_3$) spectrum for brasiliamide A (**8**)

19 Acid hydrolysis of chrysosporazine U (2)

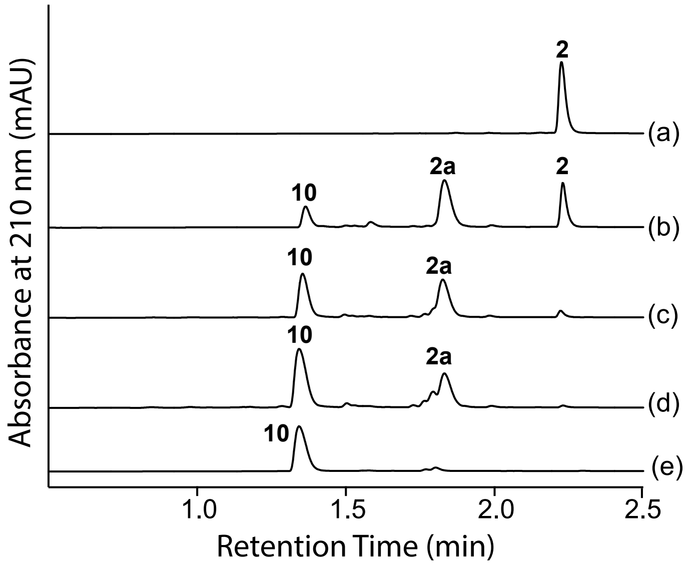

Figure S45. UPLC-DAD (210 nm) of acid hydrolysis of chrysosporazine U (**2**). (a) purified **2**; acid hydrolysis of **2** at (b) 12 hr, (c) 24 hr and (d) 36 hr; (e) purified **10**

20 Acid hydrolysis of azachrysosporazine U1 (4)

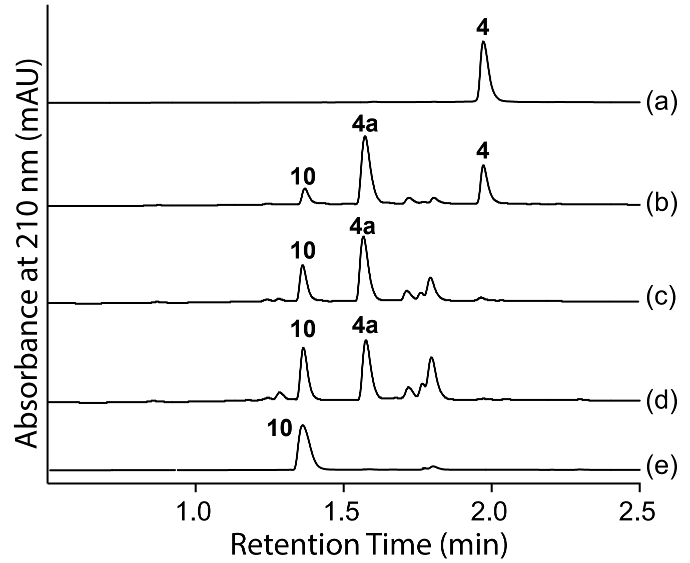

Figure S46. UPLC-DAD (210 nm) of acid hydrolysis of azachrysosporazine U1 (**4**). (a) purified **4**; acid hydrolysis of **4** at (b) 12 hr, (c) 24 hr and (d) 36 hr; (e) purified **10**

21 Acid hydrolysis of neochrysosporazine S (6)

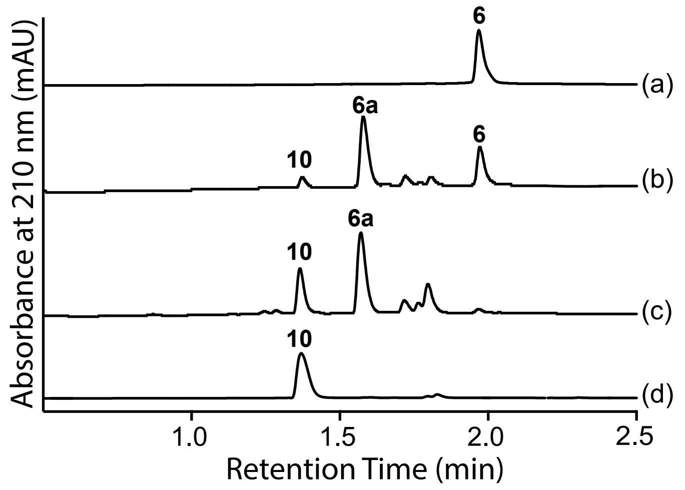

Figure S47. UPLC-DAD (210 nm) of acid hydrolysis of neochrysosporazine S (**6**). (a) purified **6**; acid hydrolysis of **6** at (b) 12 hr, (c) 24 hr and (d) purified **10**

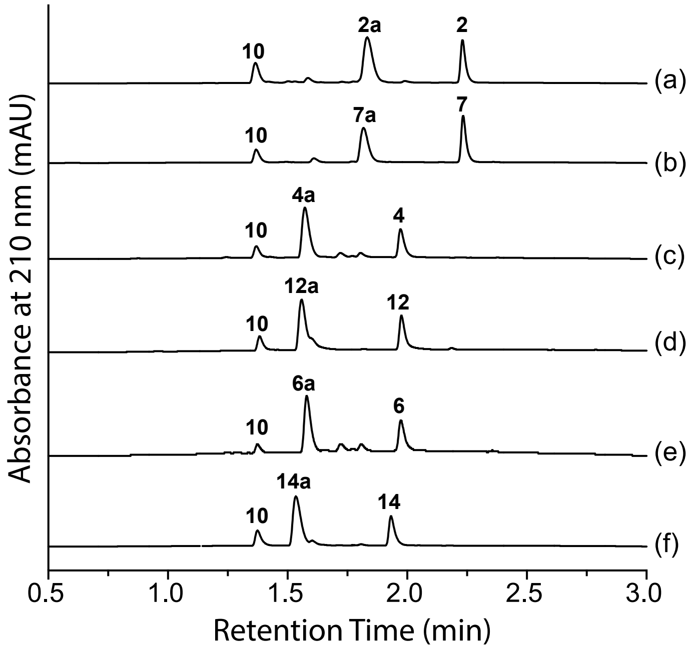

Figure S48: Comparison of UPLC-DAD retention times for 12hr hydrolysis products of (a) chrysosporazine U (**2**); (b) chrysosporazine D (**7**); (c) azachrysosporazine U1 (**4**); (d) azachrysosporazine D1 (**12**); (e) neochrysosporazine S (**6**); (f) neochrysosporazine J (**14**)

22 Antimicrobial Activity

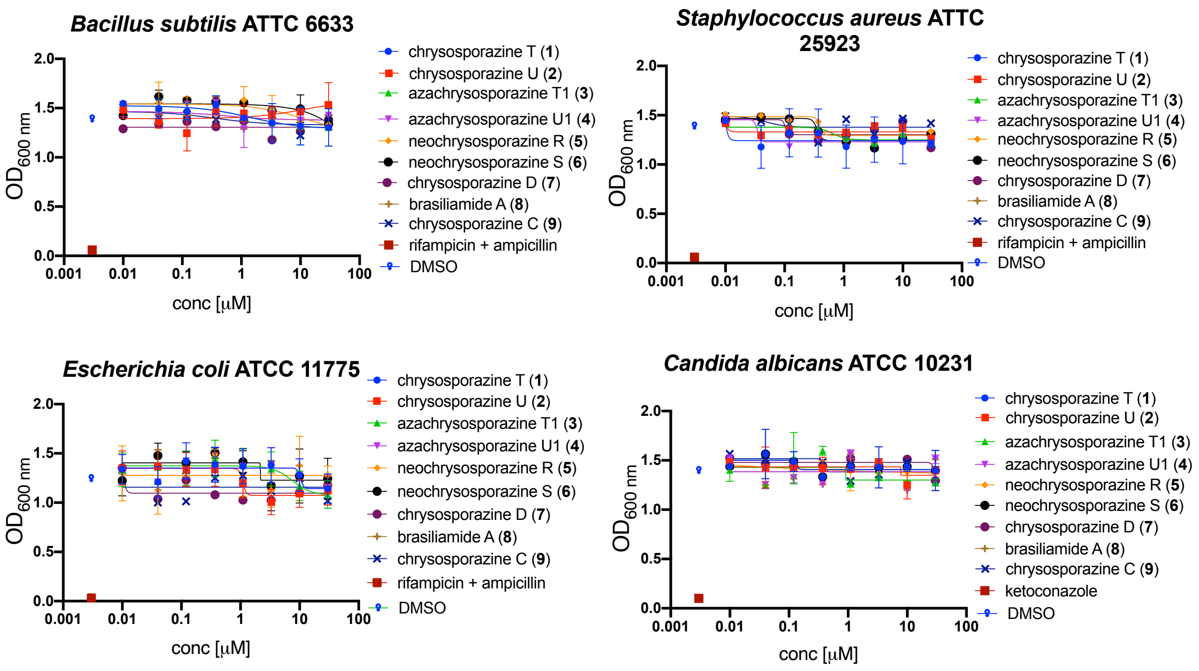

Figure S49. Growth inhibitory activity of chrysosporazines **1**–**9**

23 Cytotoxicity and P-gp activity

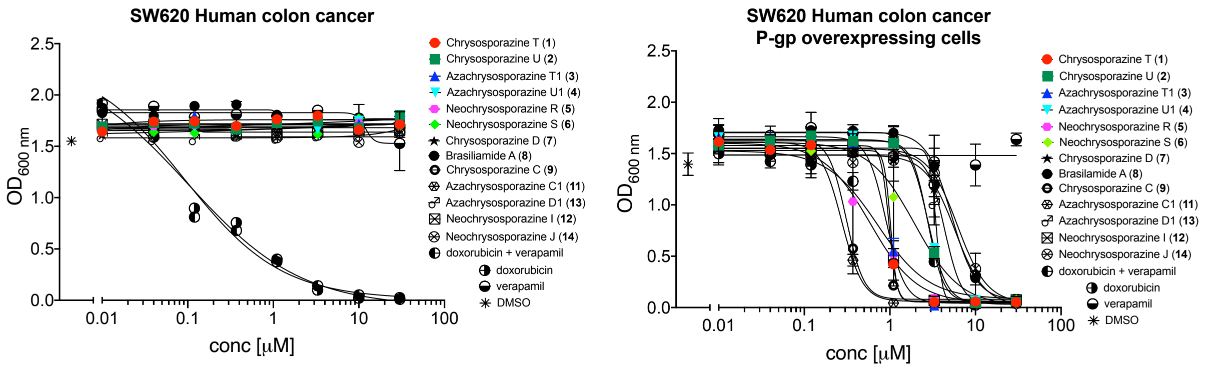

Figure S50. (A) cytotoxicity of chrypspsporazines (**1**–**14**), doxorubicin and verapamil against SW620. (B) effect of chrysosporazines or verapamil (2.5 μM) on the sensitivity of P-gp overexpressing SW620 Ad300 cancer cells to doxorubicin. 48 h MTT cytotoxicity assay was performed with a series of concentrations of doxorubicin (30 – 0.01 μM) on P-gp overexpressing SW620 Ad300 in the presence and absence of verapamil (2.5 μM) or 2.5 μM chrysosporazines (1–4). Data points are the means of ± SEM of duplicate determination from two independent cultures.

Table S7. Effect of chrysosporazines (**1–14**) on inhibition of P-gp mediated resistance to doxorubicin in SW620 Ad300 and cytotoxicity against susceptible SW620

SW620 Ad300				SW620	
**Treatment**	**IC_{50}[a] (μM)**	**FR[b]**	**GS[c]**	**Treatment**	**IC_{50}[a] (μM)**
doxorubicin	5.75	57.5	1.0	doxorubicin	0.10
+ chrysosporazine T (**1**)	0.97	9.7	5.9	chrysosporazine T (**1**)	>30
+ chrysosporazine U (**2**)	2.76	27.6	2.0	chrysosporazine U (**2**)	>30
+ azachrysosporazine T1 (**3**)	0.89	8.9	6.4	azachrysosporazine T1 (**3**)	>30
+ azachrysosporazine U1 (**4**)	2.78	27.8	2.0	azachrysosporazine U1 (**4**)	>30
+ neochrysosporazine R (**5**)	0.58	5.8	9.9	neochrysosporazine R (**5**)	>30
+ neochrysosporazine S (**6**)	1.95	19.5	2.9	neochrysosporazine S (**6**)	>30
+ chrysosporazine D (**7**)	4.36	43.6	1.3	chrysosporazine D (**7**)	>30
+ brasiliamide A (**8**)	5.27	40.5	1.1	brasiliamide A (**8**)	>30
+ chrysosporazine C (**9**)	0.31	3.1	18.5	chrysosporazine C (**9**)	>30
+ azachrysosporazine C1 (**11**)	0.27	2.7	21.3	azachrysosporazine C1 (**11**)	>30
+ azachrysosporazine D1 (**13**)	3.55	35.5	1.6	azachrysosporazine D1 (**13**)	>30
+ neochrysosporazine I (**12**)	1.01	10.1	5.7	neochrysosporazine I (**12**)	>30
+ neochrysosporazine J (**14**)	6.18	61.8	0.9	neochrysosporazine J (**14**)	>30
+ verapamil (2.5 μM)	0.71	7.1	8.1	doxorubicin + verapamil	0.092
verapamil	>30	ND	ND	verapamil	>30

[a]MTT assay showing data as means of ± SEM of two independent cultures.
[b]FR: fold-resistance was determined by dividing the IC50 value for doxorubicin for P-gp overexpressing cancer cells by the IC50 value for doxorubicin for sensitive cancer cells.
[c]GS: Gain in sensitivity was the ratio of IC50 value of doxorubicin against SW620 Ad300 without testing compound to IC50 value of doxorubicin against SW620 Ad300 with testing compound.

--: not calculated
